# Metabolite Profiling of *Allium hookeri* Leaves Using UHPLC-qTOF-MS/MS and the Senomorphic Activity of Phenolamides

**DOI:** 10.3390/nu15245109

**Published:** 2023-12-14

**Authors:** Thi-Phuong Doan, Mi Zhang, Jin-Pyo An, Jorge-Eduardo Ponce-Zea, Van-Hieu Mai, Byeol Ryu, Eun-Jin Park, Won-Keun Oh

**Affiliations:** Research Institute of Pharmaceutical Sciences, College of Pharmacy, Seoul National University, Seoul 08826, Republic of Korea; phuongdoan@snu.ac.kr (T.-P.D.); mintazhang@snu.ac.kr (M.Z.); ntopjp77@gmail.com (J.-P.A.); jepz210689@snu.ac.kr (J.-E.P.-Z.); maihieu@snu.ac.kr (V.-H.M.); estrella56@snu.ac.kr (B.R.); eunjin_p@snu.ac.kr (E.-J.P.)

**Keywords:** *Allium hookeri*, *N*-*trans*-feruloyltyramine, senomorphic effect, SASP inhibitor

## Abstract

The plant *Allium hookeri*, a member of the *Allium* genus, has a rich history of culinary and medicinal use. Recent studies have unveiled its potent antioxidant and anti-inflammatory properties. While research on *A. hookeri* has demonstrated its neuroprotective and anti-neuroinflammatory effects, the specific bioactive compounds responsible for these effects remain unidentified in prior research. This study utilized an untargeted metabolomic approach, employing HRESI-qTOF MS/MS-based molecular networking, to comprehensively profile the chemical composition of metabolites in *A. hookeri* and identify new compounds within the plant. As a result, ten compounds, comprising one novel flavonoid (**2**) and nine known compounds (**1** and **3**–**10**), were isolated and identified through NMR analysis. The inhibitory effects of all isolated compounds on the senescent cell-associated secretory phenotype (SASP), which is pivotal in neuroprotective actions, were evaluated. Biological activity testing revealed *N*-*trans*-feruloyltyramine (**7**) to be the most potent compound, effectively inhibiting SASP markers and contributing to the senomorphic activities of *A. hookeri*. These findings underscore the potential of phenolamides from *A. hookeri* as a promising source of bioactive compounds for mitigating senescence-associated diseases.

## 1. Introduction

The *Allium* genus, which is the largest within the Amaryllidaceae family, comprises over 900 species [1], including well-recognized vegetables like onion (*A. cepa*), garlic (*A. sativum*), chives (*A. schoenoprasum*), leeks (*A. ampeloprasum* var. *porrum*), and rakkyo (*A. chinense*) [2]. *Allium hookeri* Thwaites, commonly known as hooker chive or wide-leaf chives, represent a herbaceous plant indigenous to regions such as India, Sri Lanka, Myanmar, Bhutan, southwestern China [3,4], and Nepal [5]. Its recent expansion has extended into the southern areas of Korea beginning in 2012 [6,7,8]. Due to the distinctive flavor characteristic of the *Allium* genus, both the leaves and fleshy roots of *A. hookeri* are used as vegetables, holding particular significance for the locals and serving as spices or supplementary foods [5,9]. Traditionally, the roots have been utilized by local communities in India for treating coughs, colds, burns, injuries, eruptions of the skin, and wound healing [5,10]. The leaves of *A. hookeri* are also employed for medicinal purposes, including the treatment of stomach ulcers, fever reduction, and blood pressure management [5,10]. Extensive studies on the roots of *A. hookeri* have unveiled regulatory effects against asthmatic changes [11], as well as antioxidant, neuroprotective [8,12,13], antimicrobial [14], anti-diabetic [15,16], immunomodulatory [17], anti-inflammatory [18,19], and bone-forming activities [20]. Additionally, the leaves of *A. hoookeri* also exhibited anti-obesity [21], anti-inflammatory, and immunomodulating effects [17]. The phytochemical profile of *A. hookeri* includes organosulphur compounds [22], flavonoids [23], fatty acids, phenolic, and polyphenols [12,14,23,24], and essential oils [25]. Despite the numerous reported activities of *A. hookeri*, only a few studies have established a direct link between compounds and their biological activities, such as fatty acids, phenolic and polyphenolic compounds with antimicrobial activity [14] and phenolic compounds with antioxidant properties [12].

The aging of the global population is becoming an increasingly significant medical concern. While life expectancy has increased over the past century, the additional years gained have not necessarily translated into a state of good health, especially for individuals aged 60 years and older [26]. In response to the challenges posed by population aging, the World Health Organization (WHO) has formulated a definition of healthy aging, describing it as a process of developing and maintaining functional abilities that contribute to well-being in older age [27]. Furthermore, the United Nations has designated the period from 2021 to 2030 as the UN Decade of Healthy Ageing [26]. The “hallmarks” of aging refer to the accumulation of damage, including genomic instability, telomere attrition, epigenetic alterations, the loss of proteostasis, mitochondrial dysfunctions, deregulated nutrient sensing, cellular senescence, stem cell exhaustion, and altered intercellular communication [28]. Among these hallmarks, cellular senescence, characterized by irreversible cell cycle arrest, the expression of senescence-associated beta-galactosidase (SA-*β*-gal), and the senescence-associated secretory phenotype (SASP) [29], plays a significant role in complex biological processes related to aging and age-related disorders [30]. Cellular senescence also plays a crucial role in the wound-healing process, involving inflammation, new tissue formation, and tissue remodeling as a physiological response to tissue injury [31]. While senescent fibroblasts contribute positively to wound healing by limiting any excess collagen deposition through the SASP, which includes collagen-degrading matrix metalloproteinases (MMPs) [32], their persistent presence in chronic wounds impedes closure. This persistence is marked by senescence indicators, oxidative stress, and proinflammatory cytokines in fibroblasts, highlighting their role in chronic wound pathology [33,34]. As individuals age, senescent cells tend to accumulate in tissues and organs, thereby contributing to the development of age-related diseases or chronic wounds [30]. The removal of senescent cells from tissues and organs may represent a crucial strategy for preventing and treating age-related diseases, thereby increasing the health span [28]. Senotherapeutics achieve their effect by targeting the following two approaches: eliminating senescent cells (senolytics) or inhibiting senescent phenotypes such as SA-β-gal or SASP secretion (senomorphics) [28]. To date, numerous natural products, such as quercetin, fisetin, piperlongumine, and the curcumin analog, have been recognized as senolytic agents [35]. Additionally, natural senomorphics like rapamycin, resveratrol, kaempferol, apigenin, and epigallocatechin gallate (EGCG) have been documented [36], indicating the potential of natural products as a promising source for senotherapeutic drug discovery.

This study aimed to investigate the phytochemical constituents in *A. hookeri* leaves using an untargeted metabolomic approach, incorporating feature-based molecular networking (FBMN), along with the isolation and characterization of major nodes. Additionally, the research evaluated their senomorphic effects, potentially contributing to their ethnomedicinal use in wound healing and in vitro neuroprotection. This study also sought to identify the key compounds responsible for the senomorphic effects of *A. hookeri*, examining both lipopolysaccharides (LPS)-induced nitric oxide (NO) production and the bleomycin-induced senescence model.

## 2. Materials and Methods

### 2.1. Materials

Optical rotations were determined using a JASCO P-2000 polarimeter (JASCO International Co., Ltd., Tokyo, Japan). IR spectra were measured with a Nicolet 6700 FT-IR spectrometer (Thermo Electron Corp., Waltham, MA, USA). NMR data analysis was performed with a JNM-ECA 600 MHz spectrometer (Jeol Ltd., Tokyo, Japan) coupled with a 5 mm CPTCI cryoprobe (Bruker, Germany). For column chromatography, silica gel (Merck KGaA, Darmstadt, Germany, particle size 63–200 μm) and RP-18 (Merck KGaA, particle size 75 μm) were used. Sephadex LH-20 from Sigma-Aldrich (St. Louis, MO, USA) was also used. Thin-layer chromatography (TLC) was conducted using silica gel 60 F_254_ and RP-18 F_254_ plates from Merck KGaA, Darmstadt, Germany. High-performance liquid chromatography (HPLC) was carried out using a Gilson system with a UV detector (at 201 and 254 nm) and an Optima Pak C_18_ column (10 × 250 mm, particle size 5 μm, RS TECH Co., Ltd., Chungcheongbuk-do, Korea).

### 2.2. Plant Material

*A. hookeri* was purchased from the commercial stores in March 2019 and was authenticated by Professor W. K. Oh. The samples were dried at room temperature, and a voucher specimen (2019-SNU-12) was deposited in the herbarium at the College of Pharmacy, Seoul National University, Seoul, Republic of Korea.

### 2.3. UHPLC-qTOF-MS/MS Experiments

The chemical profiling of *A. hookeri* was performed using an Agilent 6530 Q-TOF mass spectrometer equipped with an Agilent 1260 Infinity UHPLC (Agilent Technologies, Santa Clara, CA, USA). The chromatographic analysis utilized a Waters Acquity UHPLC^®^ BEH C18 (100 mm × 2.1 mm, 1.7 μm) maintained at 40 °C, and LC-MS/MS analyses were conducted in the fast data-dependent acquisition (DDA) mode. The chromatographic separation was operated with the gradient of acetonitrile (contained 0.1% formic acid, B)/H_2_O (contained 0.1% formic acid, A) as follows: 0–20 min, 10–90% B; 20.1–22 min, 100% B; and 22.1–24.0 min, 10% B. Electrospray ionization (ESI) conditions included an untargeted MS scan from 100 to 1500 m/z in positive and negative ion modes. The instrumental parameters were set as follows: sheath gas temperature, 350 °C; gas flow, 10 L/min; nebulizer pressure, 30 psig. The scan source parameters encompassed the VCap (4000 V), nozzle voltage (1000 V), fragmentor (180 V), skimmer (65 V), and octopole RF peak voltages (750 V) with a collision energy of 50 eV.

### 2.4. Sample Preparation of A. hookeri for MS/MS Analysis

The fresh leaves of *A. hooker* (5 g) underwent sonication at room temperature for 1.5 h using 20 mL of 70% EtOH (3 times) at room temperature. The combined total extracts were then dried under a vacuum pump and then sequentially chromatographed using Sep-Pak^®^ Plus Short C18 (360 mg Sorbent per Cartridge, 55–105 µm, Waters Corporation, Milford, MA, USA), eluting with H_2_O, 25%, 50%, 75%, 90%, and 100% MeOH/H_2_O. All the samples were dried and diluted to a final concentration of 2.0 mg/mL with HPLC-grade MeOH for further analysis. Following drying, all samples were diluted at a concentration of 2.0 mg/mL using HPLC-grade MeOH for subsequent analysis. Prior to injection into the mass spectrometer, all samples underwent filtration through a 0.20 μm membrane filter (Advantec, Tokyo Roshi Kaisha, Japan), with each sample injection consisting of 2 μL.

### 2.5. Extraction and Isolation 

The air-dried leaves of *A. hookeri* were cut into small pieces and extracted with 70% EtOH using sonication for 90 min (three times) at room temperature. The resulting combined extract was then suspended in water and applied to a Diaion HP-20 column chromatography (CC). Elution was achieved using a MeOH/H_2_O gradient, resulting in the separation of the extract into the following three different fractions: H1 (50% MeOH), H2 (75% MeOH), H3 (100% MeOH). Fraction H3 was further subjected to normal-phase silica gel CC, eluting with an elution gradient of EtOAc/MeOH (2:1, 1:1, to 0:1) to obtain four fractions (N1-N4). Fraction N2, exhibiting the target peaks corresponding to flavones and amides, was further chromatographed on a reverse-phase C_18_ column with a mobile phase of 50–100% MeOH in water to provide 18 sub-fractions (R1-R18). Sub-fraction R2 was then processed through a Sephadex LH-20 column, with elution achieved using 100% MeOH and yielding ten fractions (L1-L10). Fraction L2 was subjected to purification via an HPLC system equipped with an RP C_18_ column, ultimately resulting in the isolation of compound **4** (5.6 mg); fraction L5 provided compound **2** (10.0 mg). Fraction L4 was further processed through an MPLC column, separating it into ten sub-fractions (M1-M10). After the purification of sub-fraction M3 through the HPLC system, compounds **1** (20.0 mg), **3** (5.0 mg), and **7** (3.8 mg) were obtained. Similarly, compounds **9** (4.3 mg) and **10** (8.5 mg) were yielded from sub-fraction M5 through the HPLC system, while sub-fraction M7 contained compounds **5** (12.5 mg), **6** (3.8 mg), and **8** (5.2 mg).

#### Physicochemical Properties of Isolated Compounds

Apigenin-7-*O*-glucuronide (**1**): pale yellow amorphous powder; ${\left[\alpha \right]}_{D}^{25}$ = −32.1 (*c* 0.1, MeOH); IR (KBr) *ν*_max_ 3298, 2908, 2818, 1736, 1662, 1607, 1498, 1443, 1349, 1299, 1250, 1175, 1105, 1086, 1061, 1026, 833, 763, 679, 629 cm^−1^; ^1^H NMR (pyridine-*d*_5_, 600 MHz) and ^13^C NMR (pyridine-*d*_5_, 150 MHz): Figures S14 and S15; HRESIMS found *m/z* 477.0899 [M + H]^+^ (calcd for C_21_H_19_O_11_ at 477.0927, *m/z* error—5.87 ppm).

Apigenin-7-*O*-[*α-*_L_-rhamnopyranosyl(1→2)]*β*-_D_-glucuronic acid methyl ester (**2**): pale yellow amorphous powder; ${\left[\alpha \right]}_{D}^{25}$ = −13.4 (*c* 0.1, MeOH); UV (MeOH) *λ*_max_ (log *ε*) 203 (−0.91), 253 (−0.67), 347 (−0.55); IR (KBr) *ν*_max_ 3370, 2926, 2382, 2357, 2307, 1748, 1658, 1608, 1513, 1443, 1344, 1298, 1248, 1179, 1099, 949, 835, 679, 604 cm^−1^; ^1^H NMR (pyridine-*d*_5_, 600 MHz) and ^13^C NMR (pyridine-*d*_5_, 150 MHz): Table 1; HRESIMS found *m/z* 605.1500 [M − H]^−^ (calcd. for C_28_H_29_O_15_ at 605.1506, *m/z* error—1.0 ppm).

Apigenin 7-*O*-glucuronide methyl ester (**3**): pale yellow amorphous powder; ${\left[\alpha \right]}_{D}^{25}$ = −19.8 (*c* 0.1, MeOH); IR (KBr) *ν*_max_ 3725, 3630, 3600, 3316, 2926, 2387, 2352, 2312, 1752, 1618, 1588, 1523, 1468, 1363, 1339, 1248, 1174, 1044, 835, 684, 670, 645 cm^−1^; ^1^H NMR (DMSO-*d*_6_, 600 MHz) and ^13^C NMR (DMSO-*d*_6_, 150 MHz): Figures S17 and S18; HRESIMS found *m/z* 461.1055 [M + H]^+^ (calcd. for C_22_H_21_O_11_ at 461.1084, *m/z* error—6.29 ppm).

Chrysoeriol-7-*O*-*β*-_D_-glucuronide (**4**): pale yellow amorphous powder; ${\left[\alpha \right]}_{D}^{25}$= +30.7 (*c* 0.1, MeOH); UV (MeOH) *λ*_max_ (log *ε*) 252 (−1.95), 350 (−1.85); IR (KBr) *ν*_max_ 3730, 3620, 3595, 3300, 2946, 2387, 2307, 1748, 1613, 1503, 1418, 1333, 1174, 1039, 689, 670, 645 cm^−1^; ^1^H NMR (DMSO-*d*_6_, 500 MHz) and ^13^C NMR (DMSO-*d*_6_, 125 MHz): Figures S22 and S23; HRESIMS found *m/z* 477.1032 [M + H]^+^ (calcd. for C_22_H_21_O_12_ at 477.1033, *m/z* error—0.2 ppm).

Chrysoeriol-7-*O*-*β*-_D_-glucuronic acid methyl ester (**5**): pale yellow amorphous powder; ${\left[\alpha \right]}_{D}^{25}$ = −64.7 (*c* 0.1, MeOH); UV (MeOH) *λ*_max_ (log *ε*) 203 (−0.90), 252 (−0.67), 348 (−0.52); IR (KBr) *ν*_max_ 3725, 3700, 3625, 3595, 3306, 2966, 2387, 2362, 2342, 2307, 1748, 1513, 1418, 1363, 1333, 1049, 1034, 1014, 689, 674, 649, 615 cm^−1^; ^1^H NMR (DMSO-*d*_6_, 600 MHz) and ^13^C NMR (DMSO-*d*_6_, 150 MHz): Figures S27 and S28; HRESIMS found *m/z* 491.1193 [M + H]^+^ (calcd. for C_23_H_23_O_12_ at 491.1190, *m/z* error 0.61 ppm).

Paprazine (**6**): pale yellow amorphous powder; ${\left[\alpha \right]}_{D}^{25}$ = −18.1 (*c* 0.1, MeOH); IR (KBr) *ν*_max_ 3717, 3696, 3627, 2972, 2386, 2351, 2302, 146, 1508, 1220, 1051, 818, 673 cm^−1^; ^1^H NMR (DMSO-*d*_6_, 500 MHz) and ^13^C NMR (DMSO-*d*_6_, 125 MHz): Figures S31 and S32; HRESIMS found *m/z* 284.1281 [M + H]^+^ (calcd. for C_17_H_18_NO_3_ at 284.1287, *m/z* error—2.11 ppm).

*N*-*trans*-feruloyltyramine (**7**): pale yellow amorphous powder; ${\left[\alpha \right]}_{D}^{25}$ = −24.7 (*c* 0.1, MeOH); IR (KBr) *ν*_max_ 3731, 3702, 3627, 3597, 3344, 2942, 2381, 2357, 2307, 1592, 1508, 1265, 1225, 1031, 823, 679 cm^−1^; ^1^H NMR (DMSO-*d*_6_, 400 MHz) and ^13^C NMR (DMSO-*d*_6_, 100 MHz): Figures S35 and S36; HRESIMS found *m/z* 314.1381 [M + H]^+^ (calcd. for C_18_H_20_NO_4_ at 314.1392, *m/z* error—3.50 ppm). 

*N*-*trans*-feruloyl-3-*O*-methyldopamine (**8**): pale yellow amorphous powder; ${\left[\alpha \right]}_{D}^{25}$ = −13.0 (*c* 0.1, MeOH); UV (MeOH) *λ*_max_ (log *ε*) 240 (−1.60), 290 (−1.54), 318 (−1.49); IR (KBr) *ν*_max_ 3737, 3726, 3702, 3627, 3592, 3305, 2972, 2938, 2381, 2357, 2312, 1752, 1602, 1508, 1269, 1220, 1126, 1036, 689, 669 cm^−1^; ^1^H NMR (DMSO-*d*_6_, 600 MHz) and ^13^C NMR (DMSO-*d*_6_, 150 MHz): Figures S40 and S41; HRESIMS found *m/z* 344.1427 [M + H]^+^ (calcd. for C_19_H_21_NO_5_ at 344.1420, *m/z* error 2.03 ppm).

5,7,4′-Trimethoxyflavone (**9**): pale yellow amorphous powder; ${\left[\alpha \right]}_{D}^{25}$ = −49.1 (*c* 0.1, MeOH); UV (MeOH) *λ*_max_ (log *ε*) 230 (−0.85), 265 (−0.62), 328 (−0.56); IR (KBr) *ν*_max_ 3349, 2942, 2351, 1642, 1607, 1518, 1458, 1424, 1349, 1255, 1220, 1181, 1160, 1126, 1091, 1051, 1026, 837, 673 cm^−1^; ^1^H NMR (DMSO-*d*_6_, 600 MHz) and ^13^C NMR (DMSO-*d*_6_, 150 MHz): Figures S45 and S46; HRESIMS found *m/z* 313.1084 [M + H]^+^ (calcd. for C_18_H_17_O_5_ at 313.1076, *m/z* error 2.6 ppm).

Diosgenin-3-*O*-[[*β*-_D_-glucopyranosyl(1→4)]-*α*-_L_-rhamnopyranosyl(1→4)]-[*α-*_L_-rhamnopyranosyl(1→2)]]*β*-_D_-glucopyranoside (**10**): pale yellow amorphous powder; ${\left[\alpha \right]}_{D}^{25}$ = −39.4 (*c* 0.1, MeOH); IR (KBr) *ν*_max_ 3414, 2933, 2898, 2362, 2312, 1542, 1458, 1378, 1309, 1135, 1036, 907, 828, 673, 645 cm^−1^; ^1^H NMR (pyridine-*d*_5_, 500 MHz) and ^13^C NMR (pyridine-*d*_5_, 125 MHz): Figures S49 and S50; HRESIMS found *m/z* 1031.5406 [M + H]^+^ (calcd. for C_28_H_29_O_15_ at 1031.5427, *m/z* error—2.04 ppm).

### 2.6. Cell Culture

RAW264.7 cells were generously supplied by Prof. S. H. Sung (Seoul National University, Seoul, Republic of Korea) and were cultured in HyClone Dulbecco’s modified Eagle’s medium (DMEM) from, (Cytiva, Logan, UT, USA). The culture conditions included incubation at 37 °C in 5% CO_2_ and supplementation with 10% fetal bovine serum (FBS), 100 units/mL of penicillin, and 100 μg/mL of streptomycin. Subculturing was conducted every 48 h.

### 2.7. LPS-Induced NO Production and Cell Viability

The cells were seeded at a density of 5 × 10^4^ cells/well in 96-well plates and allowed to incubate overnight. Once the cells adhered to the wells, lipopolysaccharide (LPS, 1 μg/mL) was added, both with and without the addition of the isolated compounds. After the treatment of the tested compounds, the Griess reagent method was employed to access the nitrate levels, which consisted of a 1:1 ratio of solution A (1% sulfanilamide) and solution B (5% phosphoric acid with 0.1% naphthyl ethylenediamine-HCl). A microplate reader (VersaMax, Molecular Devices, LLC., San Jose, CA, USA) was used to measure the absorbance at 540 nm. A standard nitrite was used to evaluate the nitrite levels in the samples. Cell viability was assessed using a 3-(4,5-dimethyl-2-thiazolyl)-2,5-diphenyl-2H-tetrazolium bromide (MTT) assay. In short, 3T3-L1 adipocytes were seeded and cultured in DMEM with 10% FBS on 96-well plates. After 24 h of incubation, the cells were treated with the test compounds, which were dissolved in a serum-free medium for one day. Subsequently, 20 µL of the 2 mg/mL MTT solution (Sigma) was added to each well and left to incubate in the dark for 4 h. After discarding the supernatant, formazan was dissolved in DMSO, and the absorbance was measured at 550 nm using a microplate reader (VersaMax, Molecular Devices, LLC., San Jose, CA, USA).

### 2.8. Bleomycin-Induced Senescence Model

The A549 human lung carcinoma epithelial cell line, resembling the type II epithelial-like cell line, was procured from the Korean Cell Line Bank (Seoul, Republic of Korea). The cells were cultured in an RPMI 1640 medium (Welgene, Deajeon, Republic of Korea) supplemented with 10% FBS (Gibco), 100 U of penicillin, and 100 mg/mL of streptomycin (Hyclone). For experiments involving bleomycin-induced senescence, A549 cells were exposed to 5 mg/mL of bleomycin sulfate (Tokyo Chemical Industry Co., Ltd., Tokyo, Japan) for 4 days in a growth medium. To evaluate the senomorphic effect, cells were co-treated with 5 mg/mL of bleomycin and the indicated compounds for 6 days. Then, RNA was extracted to compare the aging-related markers after bleomycin-induced senescence.

#### Senescence-Associated β-Galactosidase Staining

Cells were subjected to staining with the Senescence *β*-Glactosidase Staining Kit (#9860, Cell Signaling Technology, Denver, MA, USA) according to the provided guidelines. Following the staining process, the cell samples were examined under an Olympus ix70 microscope (Olympus Corporation, Tokyo, Japan).

### 2.9. RNA Extraction and Quantitative Real-Time Polymerase Chain Reaction (qRT-PCR)

Human dermal fibroblast (HDF) cells were seeded in a 6-well plate at a density of 80,000 per well. After a 3-day treatment, 250 µL of the TRIzol reagent (Invitrogen, Waltham, MA, USA) was added to each well for total RNA extraction. Subsequently, 1 µg of the total RNA, along with the M-MLV Reverse Transcriptase kit (Bioneer, Daejeon, Republic of Korea), were used for cDNA synthesis in a 20 µL reaction, which was later diluted to 100 µL and utilized as a template in the following real-time qPCR reactions. Real-time qPCR was conducted using the AccuPower^®^ 2X GreenStar™ qPCR Master Mix (Bioneer), and all reactions were executed in triplicate with the expression levels of the markers normalized to the expression level of 18s ribosomal RNA.

### 2.10. Statistical Analyses

The data underwent analysis through GraphPad PRISM 5 software (GraphPad Software, Inc., La Jolla, CA, USA) and are expressed as the mean ± standard deviation (SD) derived from triplicate experiments. To substantiate comparisons among the group means, an analysis of variance (ANOVA) was applied, followed by Tukey’s multiple comparison test for post hoc analysis. Statistical significance was established at a threshold of *p* < 0.05.

## 3. Results

### 3.1. Feature-Based Molecular Networking of the A. hookeri Leaf Extract

**Figure 1:**
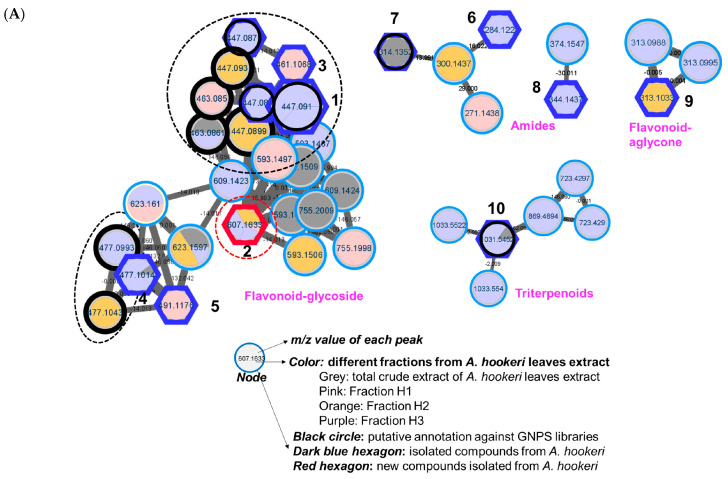

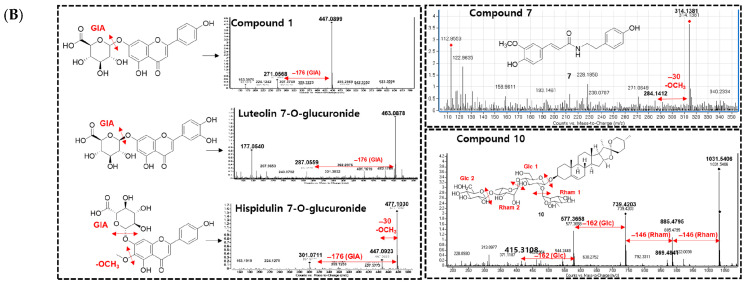
(**A**) Molecular networking of the crude extract, EtOAc, and *n*-BuOH fractions from the leaves of *Allium hookeri* using HRESI-qTOF-MS/MS data recorded in the positive mode. The black frame was assigned using GNPS, and the blue frame for the accuracy of the MN was confirmed by isolating compounds (**1–10**) from each cluster and confirming their structures to the physicochemical properties of reported compounds using spectroscopic methods. The red frame shows the new compound **2**, which was first identified from this plant. (**B**) An analysis of the cluster of interest related to flavonoid–glycoside suggested by molecular networking.

The mass data of the total extract and three fractions (H1–H3) were analyzed using MassHunter software, and the results are shown in Figure S1 (Supplementary Materials) for the positive mode. For the molecular networking analysis, the feature-based molecular networking (FBMN) generated from the GNPS platform was visualized using Cytoscape, as shown in Figure S2 (Supplementary Materials) for the full network and Figure 1A for the clusters of interest. 

As shown in Figure 1A, four clusters of interest were identified, including flavonoid-glycosides, amides, and triterpenoids. In the flavonoid–glycoside cluster, three compounds were annotated using the GNPS platform against the online database of GNPS, namely, apigenin-7-*O*-glucuronide (*m/z* 447 Da), (2*S*,3*S*,4*S*,5*R*,6*S*)-6-[2-(3,4-dihydroxyphenyl)-5-hydroxy-4-oxochromen-7-yl]oxy-3,4,5-trihydroxyoxane-2-carboxylic acid (*m/z* 463 Da), and (2*S*,3*S*,4*S*,5*R*,6*S*)-3,4,5-trihydroxy-6-[5-hydroxy-2-(4-hydroxyphenyl)-6-methoxy-4-oxochromen-7-yl]oxyoxane-2-carboxylic acid (*m/z* 477 Da). The amide cluster displayed the presence of moupinamide (*N*-*trans*-feruloyltyramine) (*m/z* 314 Da), while the triterpenoid group was suggested to contain the compound *β*-D-glucopyranoside (3*β*,22*β*,25*R*)-26-(*β*-D-glucopyranosyloxy)-22-hydroxyfurost-5-en-3-yl *O*-6-deoxy-α-_L_-mannopyranosyl-(1→2)-*O*-[6-deoxy-α-_L_-mannopyranosyl-(1→4)] (*m/z* 1031 Da). The mass data for each putative compound were analyzed in detail to understand their fragmentation pattern, which is exhibited in Figure 1B. Among these annotated compounds, compounds **1**, **7**, and **10** were isolated to confirm their structures. 

**Figure 2:**
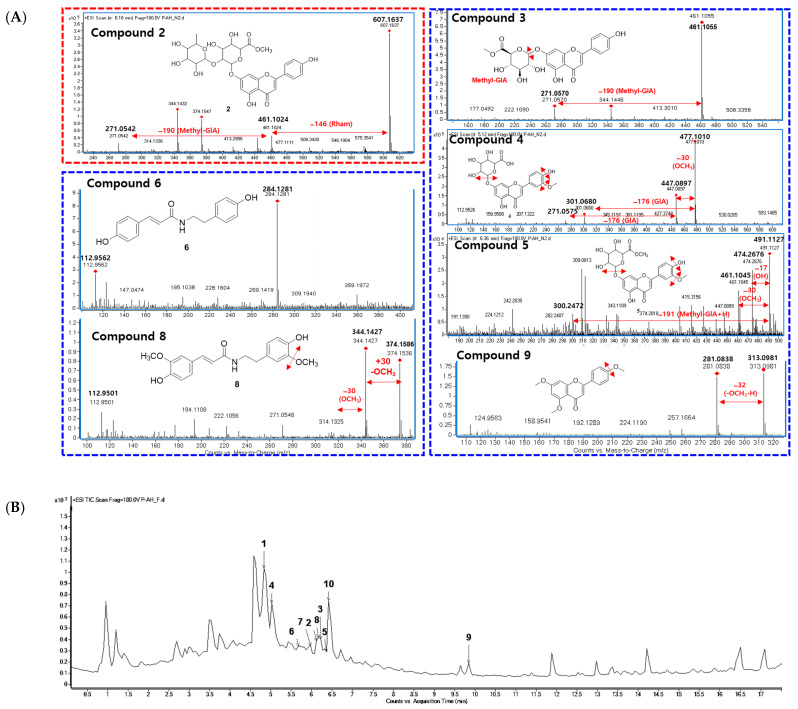
(**A**) Fragmentation analysis of isolated compounds. (**B**) Chemical profiling of isolated compounds in the *A. hookeri* leaf extract. Total ion chromatography (TIC) of *Allium hookeri* leaves recorded with HRESI-qTOF-MS/MS (positive mode). Compounds **1**–**10** were marked in the feature-based molecular networking displayed in Figure 1, and their structures are shown in Figure 3.

Compound **1**, observed within the flavonoid–glycoside cluster at an *m/z* value of 447.0899 Da (Figure 1A), was characterized as apigenin-7-*O*-glucuronide via a comparison against GNPS library matching. Additionally, its identification was corroborated via nuclear magnetic resonance (NMR) spectroscopy, referencing previously documented data from the literature [37]. The mass fragmentation analysis of compound **1** and the other identified nodes (black circle) within the flavonoid–glycoside cluster revealed the consistent presence of a glucuronic acid moiety, as evidenced by distinct *m/z* differences of 176 Da in their primary fragments (Figure 1B). A node of *m/z* 461.1068 Da was not assigned against GNPS libraries. However, this node was clustered in the same group as compound **1**, and the mass difference of 14 Da suggests that an additional methoxy (-OCH_3_) replaced a hydroxy group. Compound **3** was then isolated, and the structure was confirmed as the apigenin 7-*O*-glucuronide methyl ester via NMR spectra compared to the literature [38] and mass fragmentation analysis (Figure 2A). Similarly, compound **4**, which shared the same *m/z* value but had a different retention time to the annotated compound (2*S*,3*S*,4*S*,5*R*,6*S*)-3,4,5-trihydroxy-6-[5-hydroxy-2-(4-hydroxyphenyl)-6-methoxy-4-oxochromen-7-yl]oxyoxane-2-carboxylic acid was further confirmed by its mass fragments and NMR data to be chrysoeriol-7-*O*-*β*-D-glucuronide [39]. The mass data of compound **5** (*m/z* 491 Da) showed a loss of 191 Da, which is characteristic of a methyl-GlA, a methoxy (30 Da), and a hydroxy (17 Da). Additionally, a mass difference of 14 Da compared to compound **4** indicated that **5** had an additional methyl group attached in the GlA unit to form the methyl GlA ester. Compound **5** was also purified, and the structure was confirmed using NMR and comparing it to the reference, confirming it to be the chrysoeriol-7-*O*-*β*-D-glucuronic acid methyl ester [40]. Compound **9**, situated within a flavonoid–aglycone cluster (Figure 1A) and exhibiting an observed *m/z* value of 313.1084 Da, was conclusively identified as 5,7,4′-trimethoxyflavone. This determination was based on its NMR spectroscopic data, which sufficiently matches the literature report [41] and is further supported by mass fragmentation analysis, which revealed the presence of a fragment with an *m/z* of 287 Da [M−OCH_3_]^+^, as shown in Figure 2A.

An analysis of the fragmentation of **7** suggested that it contained a methoxy unit due to the observation of a *m/z* 30 Da (-OCH_3_) loss. Following isolation, NMR elucidation, and comparison with the literature, **7** was identified to be the same as the GNPS assignment of *N*-*trans*-feruloyltyramine [42]. Compound **6** (*m/z* 284 Da) was located in the same cluster as **7** and showed a 30 Da mass difference, indicating the absence of the methoxy group compared to **7**. Compound **6** was isolated, and its NMR confirmed the structure to be paprazine [43]. Compound **8** (*m/z* 344 Da) suggested the presence of an additional methoxy compared to **7**. The NMR of **8** was analyzed and compared with previous reports to identify it as *N*-trans-feruloyl-3-*O*-methyldopamine [43].

In the triterpenoid cluster, compound **10** (*m/z* 1031 Da) was suggested to be diosgenin-3-*O*-[[*β*-d-glucopyranosyl(1→4)]-α-l-rhamnopyranosyl(1→4)]-[α-l-rhamnopyranosyl(1→2)]]*β*-d-glucopyranoside. The mass fragmentation analysis agreed with the presence of two rhamose (Rham) units, as evidenced by the mass loss at *m/z* 146 Da. It also contained two glucose (Glc) moieties, as indicated by the mass loss at *m/z* 162 Da. After isolation, NMR analysis, and comparison to previous reports, the structure of **10** was confirmed to be annotated [44]. Within this cluster, two nodes with *m/z* values of 1033 Da were observed, differing by two hydrogens from compound **10** yet sharing identical fragments, including those at 577, 739, and 885 Da. The mass loss analysis of these nodes indicates the presence of 2 Glc (−162 Da), 1 Rham (−146 Da), and Arabinose (Ara, −148 Da). This information allows for two possible structural configurations, as displayed in Figure S4A (Supplementary Materials). Similarly, a node at 869.4894 Da within the same cluster displayed fragments indicative of mass losses corresponding to two Rham (146 Da) and a Glc (162 Da). Compared to compound **10**, this node exhibited a mass difference of 162 Da, suggesting the presence of an additional Glc. Consequently, this structure can be assigned to one of nine potential configurations, as illustrated in Figure S4B (Supplementary Materials). Furthermore, within this cluster, two nodes were identified at *m/z* 723.4238 and 723.4296 Da (with retention times of 8.15 and 13.36 min, respectively), indicating a 308 Da mass difference [= 162 (Glc) + 146 (Rham)] in comparison to compound **10**. The mass fragmentation analysis of these two peaks was conducted and searched in SciFinder, revealing 19 possible structural candidates (Figure S4C,D, Supplementary Materials). These predicted compounds vary in their absolute configurations, which cannot be discerned solely through mass analysis.

### 3.2. Isolation and Structural Elucidation of New Compound **2**

The crude extract was further applied to various column chromatography methods to isolate ten compounds, comprising one new compound (**2**) and nine known compounds (**1**, **3**–**10**), as shown in Figure 1. The chemical structures of known compounds were confirmed via mass analysis and a comparison with previous reports, as mentioned above.

**Figure 3:**
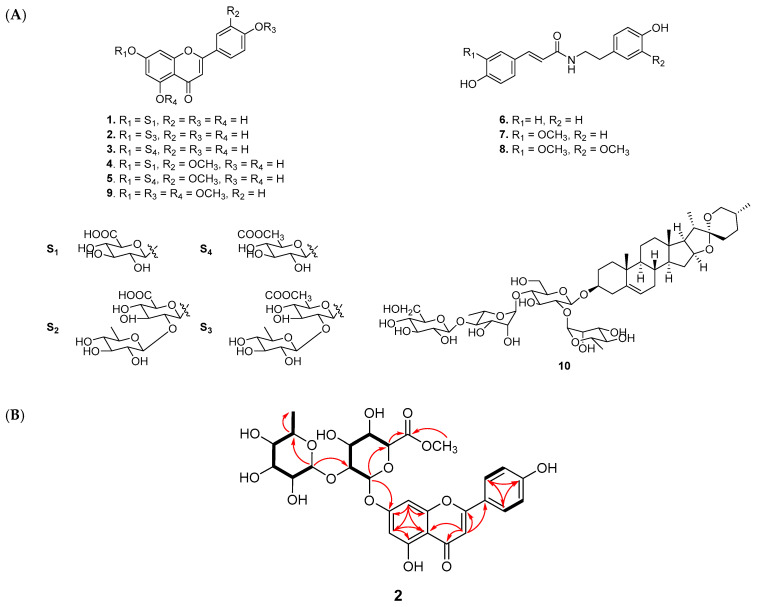
(**A**) Chemical structures of compounds **1**–**10** from *A. hookeri*. (**B**) Key COSY (bold) and HMBC (red arrows) correlations of the new compound **2** isolated from *A. hookeri*.

Compound **2** was obtained as a pale yellow powder with an established molecular formula of C_28_H_30_O_15_ based on its negative HRESIMS at 605.1500 [M − H]^−^ (calcd for C_28_H_29_O_15_ at 605.1506). In the FBMN, compound **2** was observed in a cluster of flavonoid–glycosides, suggesting that the structure of **2** has the same skeleton. As shown in Figure 3, the mass fragmentation of **2** indicated that it contained one Rham (*m/z* 146 Da) and one methyl-GlA (*m/z* 190 Da) unit. The ^1^H NMR spectrum of **2** showed five aromatic proton signals at *δ*_H_ 7.85 (2H, d, *J* = 5.1 Hz), 7.20 (overlap), 7.19 (2H, overlap), 7.04 (1H, s), one olefinic proton at *δ*_H_ 6.93 ppm, two anomeric signals at *δ*_H_ 5.44 (1H, d, *J* = 7.5 Hz) and 5.11 (1H, d, *J* = 1.8 Hz), six oxygenated protons [*δ*_H_ 4.65, t (7.8); 4.47, t (8.8); 4.52, t (8.8); 4.88, d (8.5); 4.84, br s; 4.58, d (7.7); 4.38, t (9.1); 4.80, m], one methoxy group (*δ*_H_ 3.90, s) and one methyl group [*δ*_H_ 1.18, d, (6.3)]. The ^13^C NMR data of **2** displayed 28 carbon signals, including one ketone (*δ*_C_ 183.3), an ester carbon (*δ*_C_ 170.4), 14 aromatic signals (*δ*_C_ 100.8–165.5), two anomeric carbons (*δ*_C_ 100.0, 95.6), eight carbons bearing oxygen (*δ*_C_ 70.6–78.7), and one methyl (*δ*_C_ 19.4). The HMBC data of **2** confirmed the presence of a flavone moiety via the correlations in the two aromatic rings (Figure 3B). The glucuronic acid attached to C-7 of the flavone moiety was revealed by the cross-peaks from H-1ʹ (*δ*_H_ 6.03, d, *J* = 7.3 Hz) to C-7 (*δ*_C_ 163.7). The methoxy group attached to C-3ʹ was confirmed by the correlation from the methoxy proton signal (*δ*_H_ 3.89, s) to aromatic carbon at *δ*_C_ 148.8 ppm. The rhamnose sugar moiety was identified by the presence of a methyl group (*δ*_H_ 1.18, d, (6.0)/*δ*_C_ 18.0 ppm) and its correlation with carbon signals of sugar at *δ*_C_ 69.4 and 73.0 ppm. The position of rhamnose moiety was represented by the correlation from H-1ʹʹʹ (*δ*_H_ 6.45 ppm) to C-2″ (*δ*_C_ 77.8 ppm). The absolute configuration of the sugar unit was determined following acid hydrolysis and compared with the authentic sugar. The retention time of the sugar derivative in **2** was consistent with that of authentic sugar. Therefore, the structure of **2** was identified, as shown in Figure 3, and named apigenin-7-*O*-[*α-*_L_-rhamnopyranosyl(1→2)]*β*-_D_-glucuronic acid methyl ester.

### 3.3. Bio-Activity of Isolated Compounds from A. hookeri

In our assay system, senescent human dermal fibroblast (HDF) or lung fibroblast (IMR-90) cells were induced by replicative exhaustion. SA-*β*-gal activity and SASP secretion were used to evaluate senomorphic activity. Positive SA-*β*-gal staining and overexpression of SASP factors such as IL-6, IL-8, and IL-1α were confirmed in replicative senescent HDF cells. Drugs that reduced SA-*β*-gal staining or inhibited expression of these SASP factors are thought to be senomorphic candidates.

**Figure 4:**
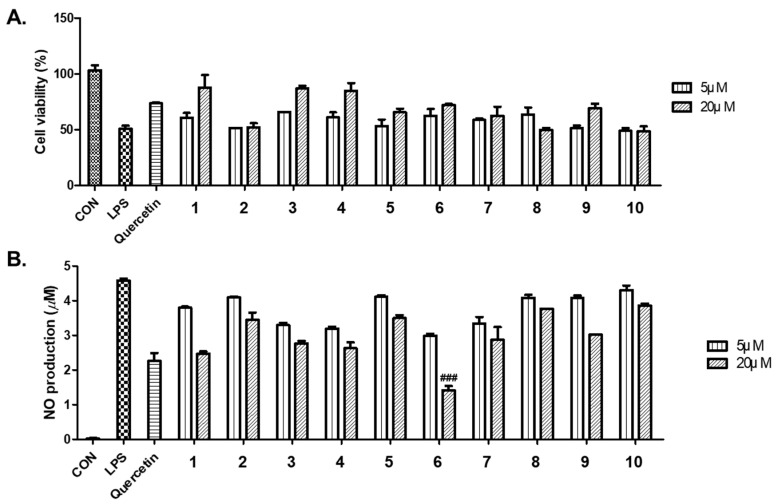
(**A**) The cell viability of compounds **1**–**10** from *A. hookeri* was measured using the EZ-Cytox cell viability assay. (**B**) The inhibitory effects of compounds **1**–**10** on LPS-induced NO production in RAW 264.7 cells compared to quercetin used as a positive control. Cell viability results indicate the relative cell viability compared to the untreated group (100%). NO assay data reflect the levels of NO production, calculated by utilizing the absorbance values at 540 nm and referencing a nitrite standard curve (white bar, untreated control group; black bar, LPS-treated control group); ^###^ *p* < 0.05 compared to the LPS-treated control group.

**Figure 5:**
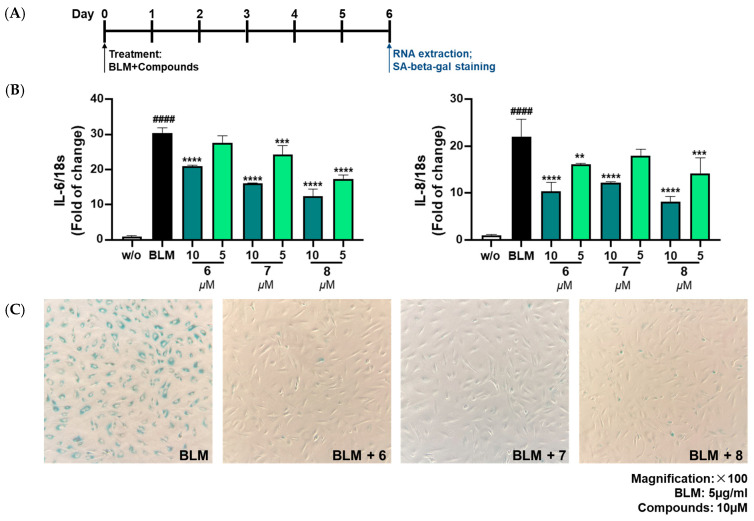
Senomorphic effects in BLM-senescent A549 cells of compounds **6**–**8** from *A. hookeri*. (**A**) A549 cells were exposed to 5 µg/mL of bleomycin (BML) and co-treated with the indicated compounds for 6 days. Then, the cells were analyzed via RNA extraction or SA-*β*-gal staining. (**B**) Compounds **6–8** were tested in a dose-dependent manner, and all three compounds showed senomorphic effects by decreasing the expression of IL-6 and IL-8 in stress-induced senescent cells. (**C**) After 6 days of treatment, SA-*β*-gal staining was significantly reduced by 10 µM in all these compounds, and no severe toxicity was detected. Data are presented as the mean ± SDs (n = 3) with one-way ANOVA with Dunnett’s multiple comparisons were performed at ** *p* < 0.01, *** *p* < 0.001 and **** *p* < 0.0001 compared to the control samples; *t*-test, ^####^ *p* < 0.0001.

**Figure 6:**
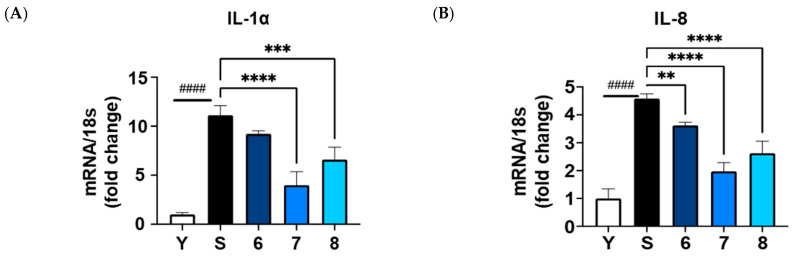

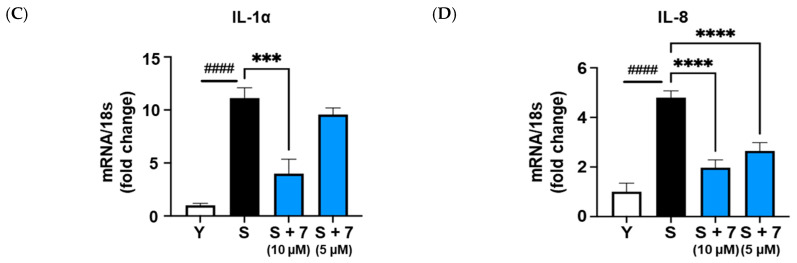
SASP inhibitor in replicative senescent HDF cells of compounds **6**–**8** from *A. hookeri.* In total, 10 µM of compounds **6**–**8** were treated on replicative senescent HDF, which was confirmed via positive SA-*β*-gal staining (Figure S43) and upregulated SASP factors such as IL-1α and IL-8. (**A**,**B**) compounds **6**–**8** significantly reduced the expression of IL-1α or IL-8 after 72 h of treatment, and compound **7** showed the best effect among them. (**C**,**D**) Compound **7** was tested and found to have a senomorphic effect in replicative senescent HDF cells in a dose-dependent manner. Data are presented as the mean ± SDs (n = 3) with one-way ANOVA with Dunnett’s multiple comparisons at ** p < 0.01, *** *p* < 0.001 and **** *p* < 0.0001 compared to the control samples; *t*-test, ^####^ *p* < 0.0001.

Ten isolated compounds (**1**–**10**) from *A. hookeri* were evaluated for their capacity in reducing NO production. The cell viability of these compounds was tested at 5 and 20 µM on LPS-induced NO production in RAW 264.7 cells compared to quercetin, a positive control. As a result, no compounds showed any cytotoxicity up to 20 µM (Figure 4A), and among them, compound **6** showed the best effect in reducing NO production (Figure 4B). In addition, compounds **6**–**8** shared similar structures, so their senomorphic effects in bleomycin (BLM)-senescent A549 cells were further evaluated. Two SASP markers, IL-6 and IL-8, were used to test senomorphic activity. As shown in Figure 5A,B, compounds **6**–**8** reduced the expression of both IL-6 and IL-8 in stress-induced senescent A549 cells that were exposed to 5 µM of BML after 6 days of treatment. The dose-dependent manner of each compound was also tested, and compounds **6**–**8** were found to have senomorphic effects by decreasing the expression of IL-6 and IL-8 (Figure 5B). After 6 days of treatment, the SA-*β*-gal staining was significantly reduced by 10 µM without any detected toxicity for all three compounds (Figure 5C). Furthermore, the SASP inhibitory activities of **6**–**8** in replicative senescent HDF cells were tested. The compounds were treated at 10 µM on replicative senescent HDF cells, which were confirmed to be senescent through positive SA-*β*-gal staining (Figure S52, Supplementary Materials) and upregulated SASP makers, including IL-1α and IL-8. After 72 h of treatment, compounds **6**–**8** significantly reduced the expression of IL-1α and IL-8 (Figure 6A,B). Among them, compound **7** showed the best effect in replicative senescent HDF cells in a dose-dependent manner. As a result, compound **7** showed senomorphic activities in replicative senescent HDF cells at 5 and 10 µM by significantly reducing the expression levels of both IL-1α and IL-8 (Figure 6C,D).

## 4. Discussion

In this study, a new compound (**2**) was identified, alongside nine known compounds (**1**, **3**–**10**), isolated from *A. hookeri*. The application of the feature-based molecular networking (FBMN) strategy not only provided a detailed chemical profile of metabolites in this extensively studied plant but also proved effective in isolating a novel compound from *A. hookeri*, thereby enriching its chemical diversity.

The senomorphic effects of all the isolated phytochemicals, characterized by a reduction in SASP markers, including IL-6, IL-8, IL-1α, and SA-*β*-gal, staining were evaluated. The results indicate that *N-trans*-feruloyltyramine (**7**) exhibits the most promising SASP-inhibitory activity among the isolated compounds. Notably, cellular senescence is integral to the wound-healing process, with the persistent presence of senescence-associated secretory phenotypes (SASP) marked by indicators of senescence, oxidative stress, and proinflammatory cytokines in fibroblasts, highlighting their involvement in the chronic wound pathology [33,34]. Consequently, the inhibition of SASP markers could potentially contribute to wound healing, aligning with the traditional use of *A. hookeri*. Furthermore, this is the first report of the senomorphic activity of *N-trans*-feruloyltyramine, which has previously been demonstrated to possess a wide range of biological effects, such as protection against scopolamine-induced cholinergic dysfunction on the cortex and hippocampus [45] and *β*-amyloid peptide-induced neurotoxicity [46], the inhibition of melanin biosynthesis [47], antioxidant, cytotoxic activities, and protection against H_2_O_2_-induced oxidative damage [48]. 

Of particular interest, the active compounds (**6**–**8**) had a similar skeleton to avenanthramide C (Avn C), with the only difference being the phenyl ethyl moieties of **6–8** and the unit of benzoic acid of avenanthramide C. Avenanthramide C has recently been extensively studied for its protective effects against pediatric pneumonia [49], allergic inflammation [50], its senomorphic effect [51], and ability to restore impaired plasticity and cognition in a mice model of Alzheimer’s disease [52]. The in vitro results from these studies suggested that Avn C reduces cytokines, including IL-4 [50], IL-6 [50,51], IL-8 [51], TNF-α [50,51], and SASP [51]. These findings are consistent with the present study’s data, which demonstrate the potential of phenolamides from *A. hookeri* to inhibit SASP in senescent cells and contribute to their senomorphic activities. Hence, its natural amide skeleton could be a promising analog for developing drugs to treat age-related diseases.

This study leveraged advanced analytical techniques, specifically UHPLC-qTOF-MS/MS-based molecular networking, to rapidly elucidate the secondary metabolite profile of hooker chive (*A. hookeri*), a plant of nutritional and medicinal significance. This approach facilitated the identification of a novel compound within the plant. Furthermore, the senomorphic activity of *A. hookeri* was elucidated for the first time, with particular attention given to the active skeleton, phenolamides, and the principal compound, *N-trans*-feruloyltyramine. This revelation establishes a link between the traditional uses of the plant and its in vitro physiological effects. Nevertheless, this study is not without limitations. Firstly, the chemical profiling of *A. hookeri* relied on putative constructions derived from GNPS libraries, which may have omitted certain well-established compounds that were not present in shared repositories or that were misaligned due to the structural isomers inherent in natural products. Secondly, the investigation has yet to unveil the mechanistic basis underlying the senomorphic effects exerted by the lead compound.

## 5. Conclusions

In summary, the study employed HRESI-qTOF-MS/MS-based molecular networking to the phytochemical profile of *A. hookeri* leaves, revealing flavonoid–glycosides, flavonoid–aglycones, triterpenoids, and phenolamides. Subsequently, ten compounds, comprising one new flavonoid (apigenin-7-*O*-[*α-*_L_-rhamnopyranosyl(1→2)]*β*-_D_-glucuronic acid methyl ester) and nine known ones, were isolated and identified through NMR analysis. An evaluation of these compounds demonstrated their impact on the senescent cell-associated secretory phenotype (SASP), with *N-trans*-feruloyltyramine (**7**) emerging as the most potent inhibitor. This underscores its crucial role in *A. hookeri*’s senomorphic activities, suggesting the potential of phenolamides as valuable bioactive compounds for combating senescence-associated diseases.

## Figures and Tables

**Table 1:** ^1^H and ^13^C NMR data of compound **2**.

Pos.	2	
	*δ*_C_ ^a^	*δ*_H_ (*J* in Hz) ^b^
2	165.5	
3	104.5	6.93, s
4	183.3	
5	158.3	
6	95.6	7.20, overlap.
7	163.7	
8	100.8	7.04, s
9	163.4	
10	107.4	
1′	122.4	
2′	129.5	7.85, d (5.1)
3′	117.4	7.19, overlap.
4′	163.3	
5′	117.4	
6′	129.5	
1″	100.0	6.03, d (7.3)
2″	77.8	4.65, t (7.8)
3″	78.7	4.47, t (8.8)
4″	73.3	4.52, t (8.8)
5″	77.5	4.88, d (8.5)
-C=O	170.4	
-COOCH_3_	52.6	3.62, s
4′-OCH_3_		
1‴	103.1	6.45, s
2‴	72.9	4.84, br s
3‴	73.2	4.58, d (7.7)
4‴	74.5	4.38, t (9.1)
5‴	70.6	4.80, m
6‴	19.4	1.85, d (6.0)

^a^ Recorded in Pyridine-*d_5_* at 600 MHz, ^b^ Recorded in Pyridine-*d_5_* at 150 MHz.

## Data Availability

Data available in a publicly accessible repository.

## Supplementary Materials

The following supporting information can be downloaded at: https://www.mdpi.com/article/10.3390/nu15245109/s1, Figure S1. Total ion chromatography (TIC) using HR-ESI -qTOF-MS/MS of *A. hookeri* leaf extract and its fractions in positive mode. Figure S2. Total ion chromatography (TIC) of *A. hookeri* leaf extract and Sep-Pak fractions (0–100% MeOH/H_2_O) using HR-ESI -qTOF-MS/MS in positive mode, Figure S3. Feature-based molecular networking of fractions and crude extract from leaves of *Allium hookeri* using HRESI-qTOF-MS/MS. Mass data was recorded in positive mode, Figure S4. Chemical structures of putative identification of components from leaves of *Allium hookeri* using HRESI-qTOF-MS/MS-based molecular networking, Figure S5. Mass/mass fragmentation of triterpenoid cluster in FBMN, Figure S6. HRESIMS data of compound **2**, Figure S7. IR(KBr) spectrum of compound **2**, Figure S8. ^1^H NMR spectrum of compound **2** (600 MHz, pyridine-*d*_5_), Figure S9. ^13^C NMR spectrum of compound **2** (150 MHz, pyridine-*d*_5_), Figure S10. HSQC spectrum of compound **2**, Figure S11. HMBC spectrum of compound **2**, Figure S12. The COSY spectrum of compound **2**, Figure S13. HRESIMS/MS of compound **1**, Figure S14. IR(KBr) spectrum of compound **1**, Figure S15. ^1^H NMR spectrum of compound **1** (400 MHz, DMSO-*d*_6_), Figure S16. HRESIMS/MS of compound **3**, Figure S17. IR(KBr) spectrum of compound **3**, Figure S18. ^1^H NMR spectrum of compound **3** (600 MHz, DMSO-*d*_6_), Figure S19. ^13^C NMR spectrum of compound **3** (150 MHz, DMSO-*d*_6_), Figure S20. HRESIMS data of compound **4**, Figure S21. UV spectrum of compound **4**, Figure S22. IR(KBr) spectrum of compound **4**, Figure S23. ^1^H NMR spectrum of compound **4** (500 MHz, DMSO-*d*_6_), Figure S24. ^13^C NMR spectrum of compound **4** (125 MHz, DMSO-*d*_6_), Figure S25. HRESIMS data of compound **5**, Figure S26. UV spectrum of compound **5**, Figure S27. IR(KBr) spectrum of compound **5**, Figure S28. ^1^H NMR spectrum of compound **5** (600 MHz, DMSO-*d*_6_), Figure S29. ^13^C NMR spectrum of compound **5** (150 MHz, DMSO-*d*_6_), Figure S30. HRESIMS data of compound **6**, Figure S31. IR(KBr) spectrum of compound **6**, Figure S32. ^1^H NMR spectrum of compound **6** (500 MHz, DMSO-*d*_6_), Figure S33. ^13^C NMR spectrum of compound **6** (125 MHz, DMSO-*d*_6_), Figure S34. HRESIMS data of compound **7**, Figure S35. IR(KBr) spectrum of compound **7**, Figure S36. ^1^H NMR spectrum of compound **7** (400 MHz, DMSO-*d*_6_), Figure S37. ^13^C NMR spectrum of compound **7** (100 MHz, DMSO-*d*_6_), Figure S38. HRESIMS data of compound **8**, Figure S39. UV spectrum of compound **8**, Figure S40. IR(KBr) spectrum of compound **8**, Figure S41. ^1^H NMR spectrum of compound **8** (600 MHz, DMSO-*d*_6_), Figure S42. ^13^C NMR spectrum of compound **8** (150 MHz, DMSO-*d*_6_), Figure S43. HRESIMS/MS data of compound **9**, Figure S44. UV spectrum of compound **9**, Figure S45. IR(KBr) spectrum of compound **9**, Figure S46. ^1^H NMR spectrum of compound **9** (600 MHz, DMSO-*d*_6_), Figure S47. ^13^C NMR spectrum of compound **9** (150 MHz, DMSO-*d*_6_), Figure S48. HRESIMS/MS data of compound **10**, Figure S49. IR(KBr) spectrum of compound **10**, Figure S50. ^1^H NMR spectrum of compound **10** (500 MHz, pyridine-*d*_5_), Figure S51. ^13^C NMR spectrum of compound **10** (125 MHz, pyridine-*d*_5_), Figure S52. Inhibitory effects of fractions and single compounds from *A. hookeri* on BLM-induced senescence system in RAW 264.7 cells using nitric oxide (NO) assay kit, Figure S53. SA-β-gal-positive senescent cells on replicative senescent HDF cell, Table S1. Putative identification of components from leaves of *Allium hookeri* using HRESI-qTOF-MS/MS-based molecular networking.
