# Supplementary material for: Metabolite Profiling of Allium hookeri Leaves Using UHPLC-qTOF-MS/MS and the Senomorphic Activity of Phenolamides
Source: Nutrients. 2023 Dec 14;15(24):5109. doi: 10.3390/nu15245109 (PMC10747020; doi:10.3390/nu15245109)
Supplement: Supplementary file 1 [file nutrients-15-05109-s001.zip › nutrients-2685794-supplementary.pdf]

Supplementary Materials

Metabolite Profiling of *Allium hookeri* Leaves using UHPLC-qTOF-MS/MS and Senomorphic Activity of Phenolamides

Thi-Phuong Doan [1], Mi Zhang [1], Jin-Pyo An [1], Jorge-Eduardo Ponce-Zea [1], Van-Hieu Mai [1], Byeol Ryu [1], Eun-Jin Park [1], and Won-Keun Oh [1,*]

[1] Research Institute of Pharmaceutical Sciences, College of Pharmacy, Seoul National University, Seoul 08826, Republic of Korea; phuongdoan@snu.ac.kr (T.P.D); mintazhang@snu.ac.kr (M.Z); ntopjp77@gmail.com (J.P.A); jepz210689@snu.ac.kr (J.E.P); maihieu@snu.ac.kr (V.H.M); estrella56@snu.ac.kr (B.R); eunjin_p@snu.ac.kr (E.J.P); wkoh1@snu.ac.kr (W.K.O)

* Correspondence: wkoh1@snu.ac.kr; Tel.: +82-2-880-7872

Table of contents

Figure S1. Total ion chromatography (TIC) using HR-ESI -qTOF-MS/MS of *A. hookeri* leaves extract and its fractions in positive mode. 4
Figure S2. Total ion chromatography (TIC) of *A. hookeri* leaves extract and Sep-Pak fractions (0–100% MeOH/H_2O) using HR-ESI -qTOF-MS/MS in positive mode. 5
Figure S3. Feature-based molecular networking of fractions and crude extract from leaves of *Allium hookeri using* HRESI-qTOF-MS/MS. Mass data was recorded in positive mode........6
Figure S4. Chemical structures of putative identification of components from leaves of *Allium hookeri* using HRESI-qTOF-MS/MS-based molecular networking. 7
Figure S5. Mass/mass fragmentation of triterpenoid cluster in FBMN 8
Figure S6. HRESIMS data of compound **2** 9
Figure S7. IR(KBr) spectrum of compound **2** 9
Figure S8. ^{1}H NMR spectrum of compound **2** (600 MHz, pyridine-d_5) 10
Figure S9. ^{13}C NMR spectrum of compound **2** (150 MHz, pyridine-d_5) 11
Figure S10. HSQC spectrum of compound **2** 12
Figure S11. HMBC spectrum of compound **2** 13
Figure S12. COSY spectrum of compound **2** 14
Figure S13. HRESIMS/MS of compound **1** 15
Figure S14. IR(KBr) spectrum of compound **1** 15
Figure S15. ^{1}H NMR spectrum of compound **1** (400 MHz, DMSO-d_6) 16
Figure S16. HRESIMS/MS of compound **3** 17
Figure S17. IR(KBr) spectrum of compound **3** 17
Figure S18. ^{1}H NMR spectrum of compound **3** (600 MHz, DMSO-d_6) 18
Figure S19. ^{13}C NMR spectrum of compound **3** (150 MHz, DMSO-d_6) 19
Figure S20. HRESIMS data of compound **4** 20
Figure S21. UV spectrum of compound **4** 20
Figure S22. IR(KBr) spectrum of compound **4** 20
Figure S23. ^{1}H NMR spectrum of compound **4** (500MHz, DMSO-d_6) 21
Figure S24. ^{13}C NMR spectrum of compound **4** (125 MHz, DMSO-d_6) 22
Figure S25. HRESIMS data of compound **5** 23
Figure S26. UV spectrum of compound **5** 23
Figure S27. IR(KBr) spectrum of compound **5** 23
Figure S28. ^{1}H NMR spectrum of compound **5** (600 MHz, DMSO-d_6) 24
Figure S29. ^{13}C NMR spectrum of compound **5** (150 MHz, DMSO-d_6) 25
Figure S30. HRESIMS data of compound **6** 26
Figure S31. IR(KBr) spectrum of compound **6** 26
Figure S32. ^{1}H NMR spectrum of compound **6** (500 MHz, DMSO-d_6) 27
Figure S33. ^{13}C NMR spectrum of compound **6** (125 MHz, DMSO-d_6) 28
Figure S34. HRESIMS data of compound **7** 29
Figure S35. IR(KBr) spectrum of compound **7** 29
Figure S36. ^{1}H NMR spectrum of compound **7** (400 MHz, DMSO-d_6) 30
Figure S37. ^{13}C NMR spectrum of compound **7** (100 MHz, DMSO-d_6) 31
Figure S38. HRESIMS data of compound **8** 32
Figure S39. UV spectrum of compound **8** 32
Figure S40. IR(KBr) spectrum of compound **8** 32

Figure S41. ^{1}H NMR spectrum of compound **8** (600 MHz, DMSO-d_6)................................33
Figure S42. ^{13}C NMR spectrum of compound **8** (150 MHz, DMSO-d_6)...............................34
Figure S43. HRESIMS/MS data of compound **9**...35
Figure S44. UV spectrum of compound **9** 35
Figure S45. IR(KBr) spectrum of compound **9**...35
Figure S46. ^{1}H NMR spectrum of compound **9** (600 MHz, DMSO-d_6)................................36
Figure S47. ^{13}C NMR spectrum of compound **9** (150 MHz, DMSO-d_6)...............................37
Figure S48. HRESIMS/MS data of compound **10**...38
Figure S49. IR(KBr) spectrum of compound **10**...38
Figure S50. ^{1}H NMR spectrum of compound **10** (500 MHz, pyridine-d_5)............................39
Figure S51. ^{13}C NMR spectrum of compound **10** (125 MHz, pyridine-d_5)...........................40
Figure S52. Inhibitory effects of fractions and single compounds from *A. hookeri* on BLM-induced senescence system in RAW 264.7 cells using nitric oxide (NO) assay kit...............41
Figure S53. SA-*β*-gal-positive senescent cells on replicative senescent HDF cell................42

Table S1. Putative identification of components from leaves of *Allium hookeri* using HRESI-qTOF-MS/MS-based molecular networking…………………………………………………42

Figure S1. Total ion chromatography (TIC) using HR-ESI -qTOF-MS/MS of *A. hookeri* leaves extract and its fractions in positive mode.

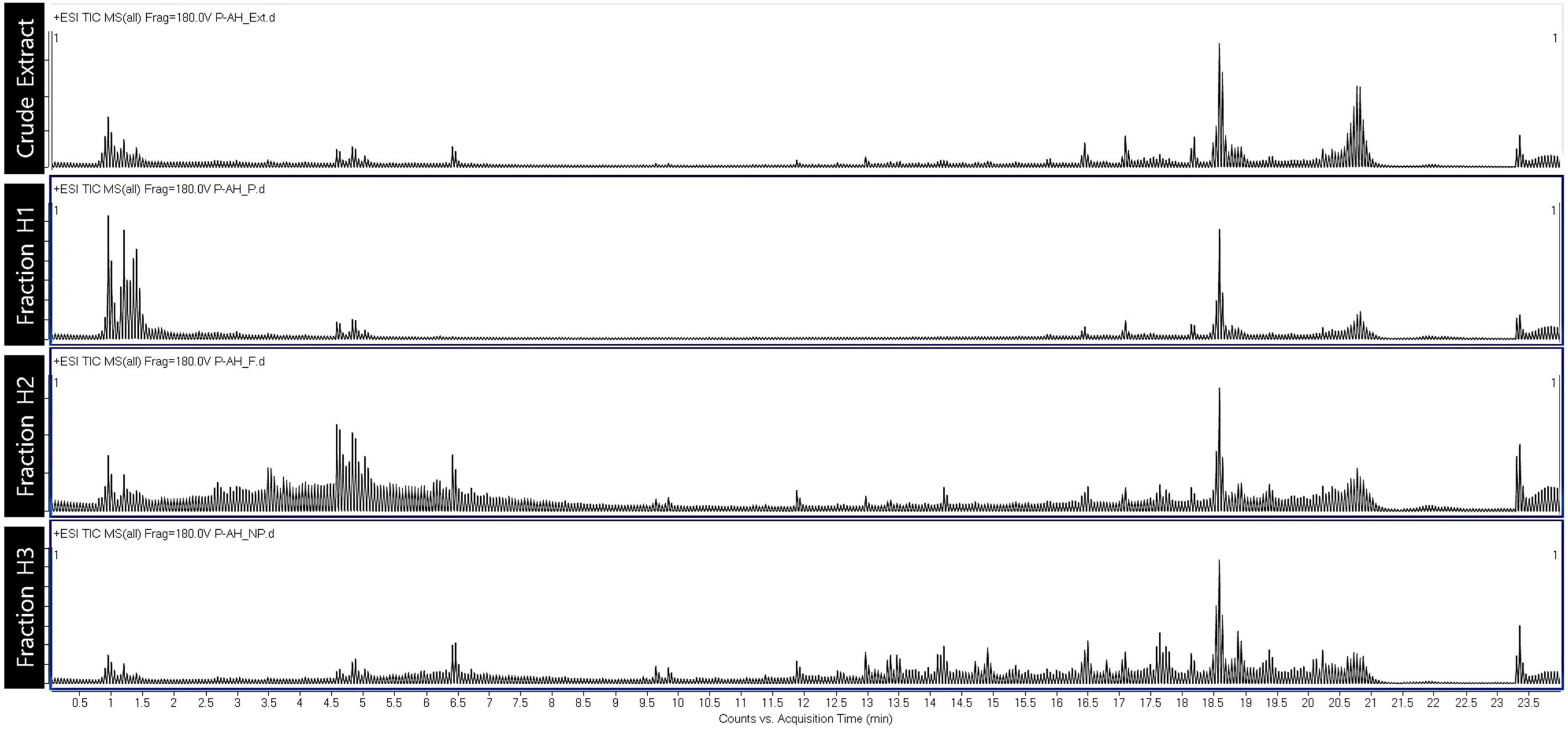

Figure S2. Total ion chromatography (TIC) of *A. hookeri* leaf extract and Sep-Pak fractions (0–100% MeOH/H_2O) using HR-ESI -qTOF-MS/MS in positive mode.

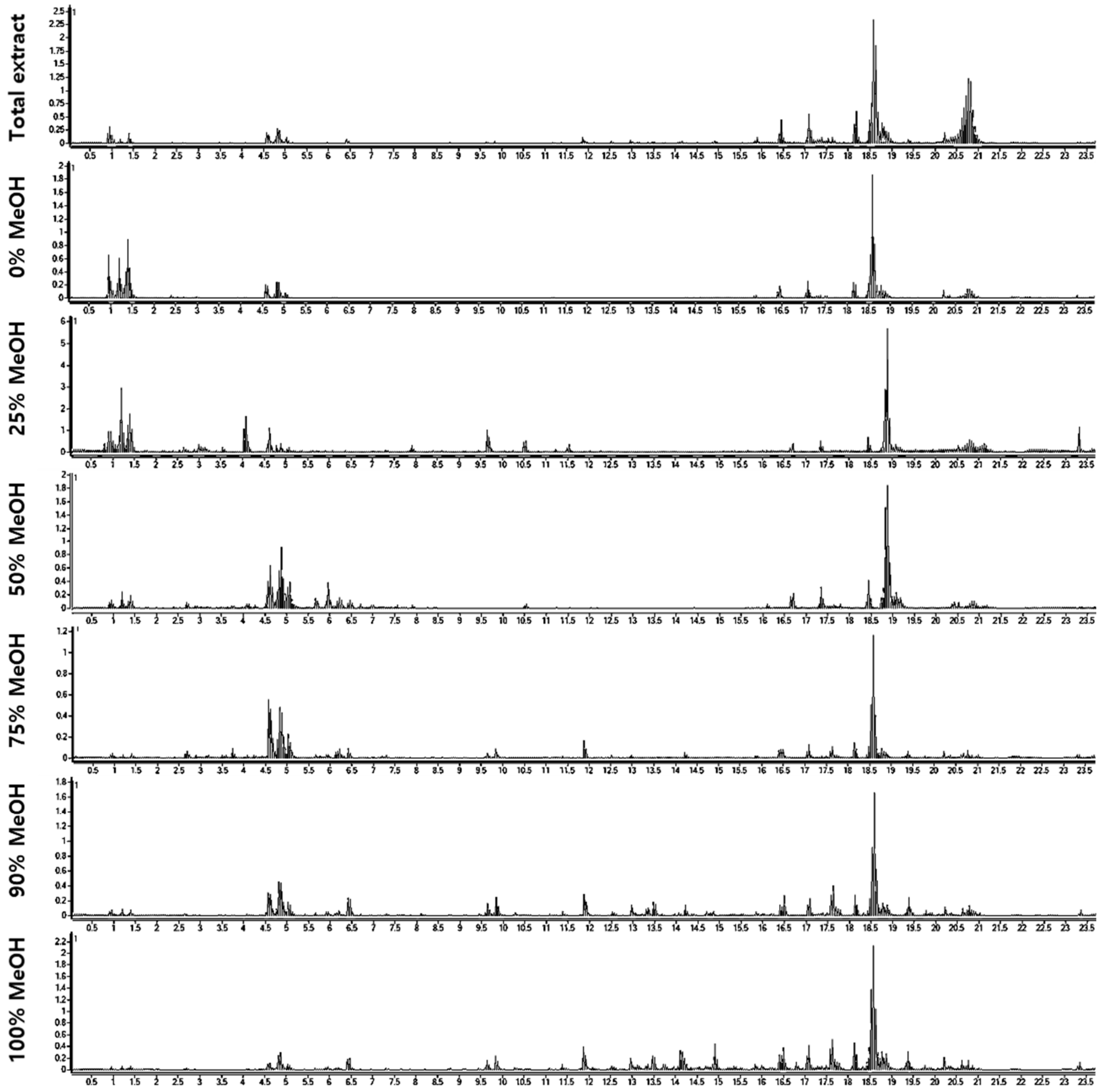

Figure S3. Feature-based molecular networking of fractions and crude extract from leaves of *Allium hookeri using* HRESI-qTOF-MS/MS. Mass data was recorded in positive mode. Data pre-processing using Mzmine3, FBMN was created at GNPS platform and can be found at https://gnps.ucsd.edu/ProteoSAFe/status.jsp?task=b6f262ecf9994b82bab562336b008eeb. Data visualzation using Cytoscape software.

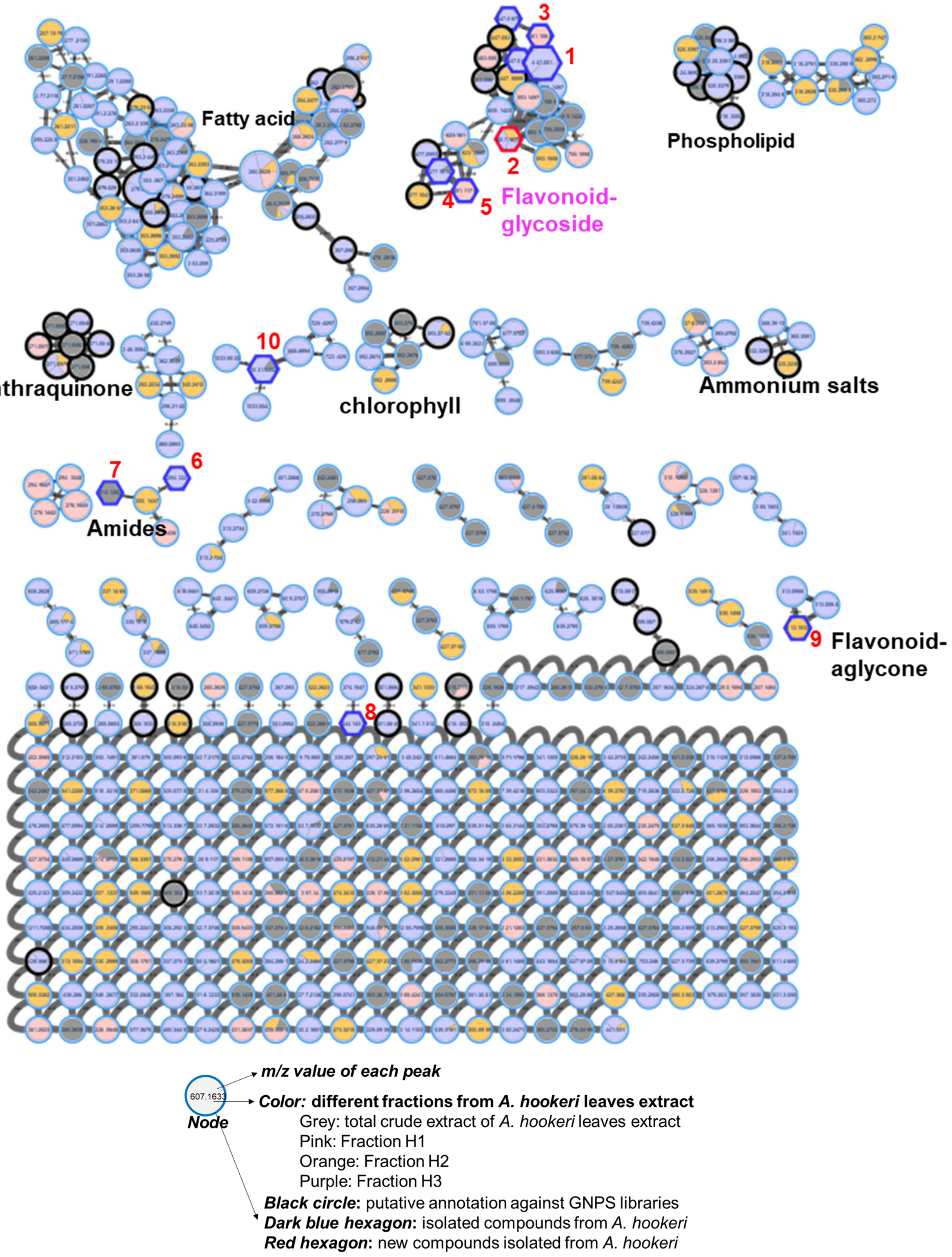

Figure S4. Chemical structures of putative identification of components from leaves of *Allium hookeri* using HRESI-qTOF-MS/MS-based molecular networking.
HRESI-qTOF-MS/MS data recorded in positive mode selective for assignment with *m/z* error less than 10 ppm. Details of each compound including retention, *m/z*, *m/z* error, and GNPS link of compound assignment can be found in Table S1. Molecular networking was created at GNPS (https://gnps.ucsd.edu/ProteoSAFe/status.jsp?task=b6f262ecf9994b82bab562336b008eeb)

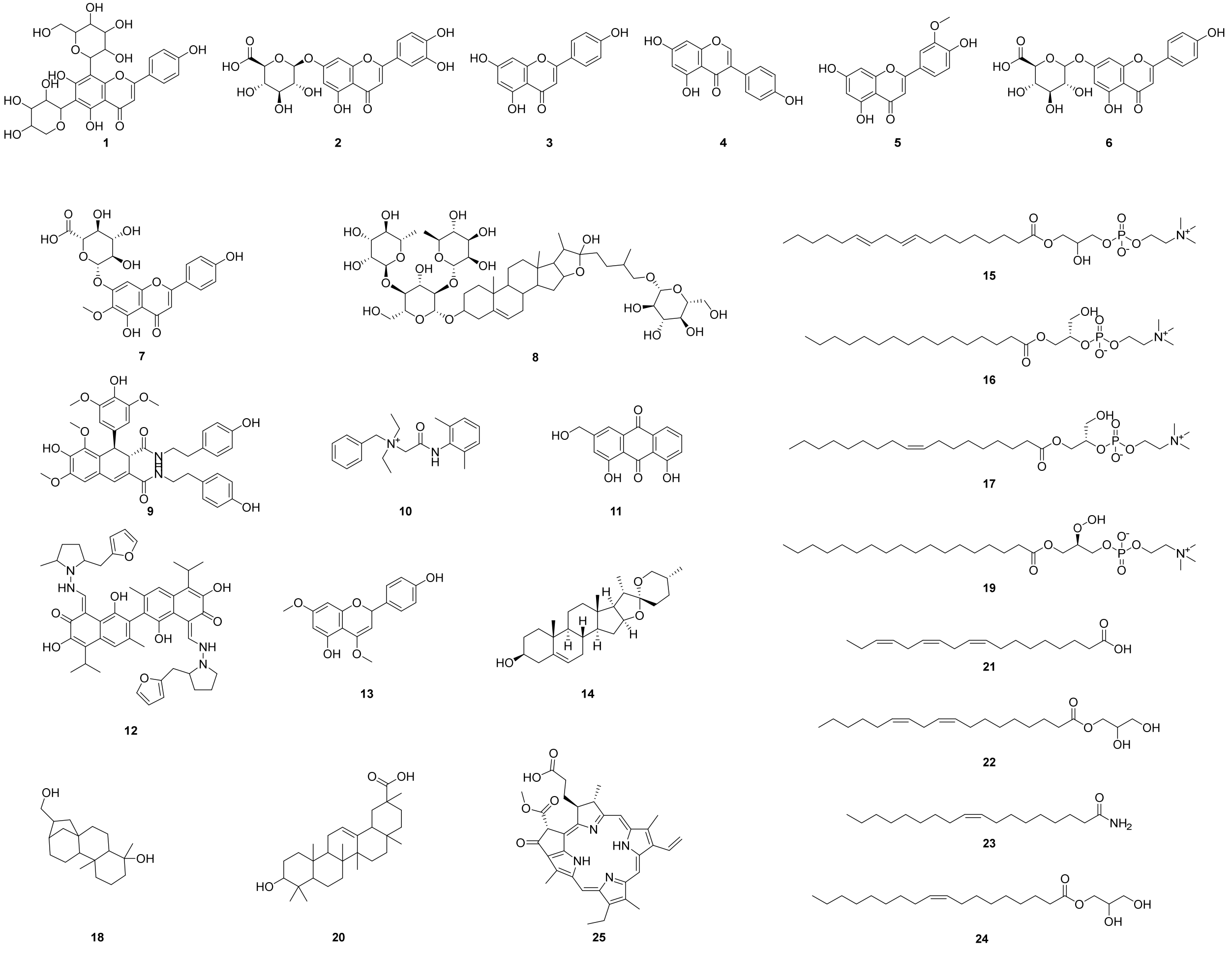

Figure S5. Mass/mass fragmentation of triterpenoid cluster in FBMN

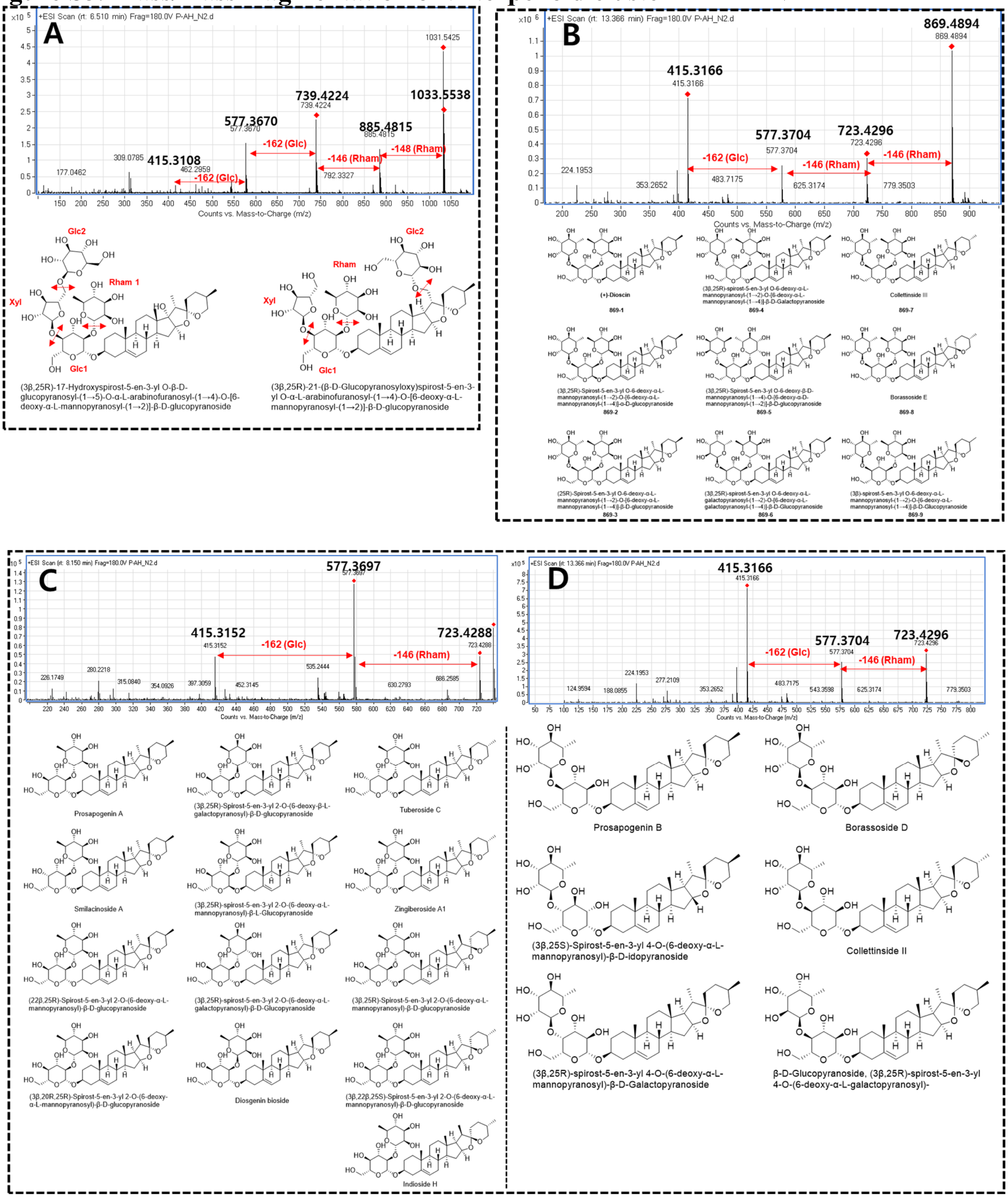

Figure S6. HRESIMS data of compound 2

Mass	RA	Calc. Mass	mDa	PPM	DBE	Formula	i-FIT	i-FIT Norm	Fit Conf %	Frag. Mass	Fragment details	C	H	O
605.1500	100.00	605.1506	-0.6	-1.0	14.5	C28 H29 O15	125.9	0.000	100.00	269.0447	C15 H9 O5 : -0.3 (-1.1)	28	29	15
		605.1542	-4.2	-6.9	36.5	C46 H21 O2	137.8	11.862	0.00			46	21	2

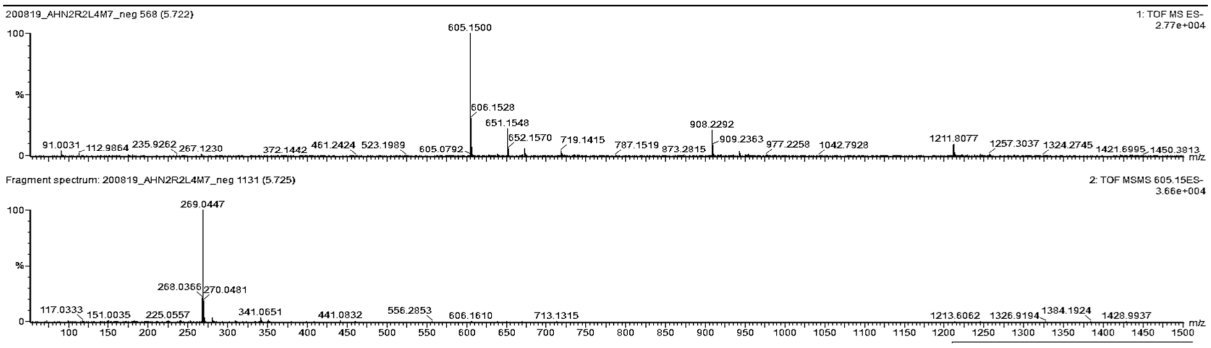

Figure S7. IR(KBr) spectrum of compound 2

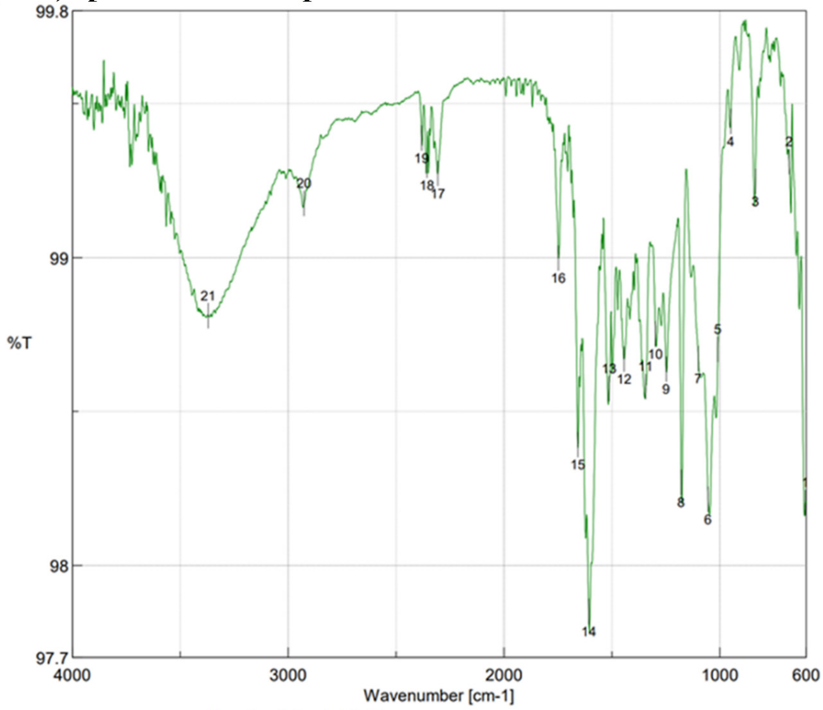

Result of Peak Picking

No.	Position	Intensity	No.	Position	Intensity
1	604.574	98.2035	2	679.785	99.3108
3	835.026	99.2461	4	949.77	99.4409
5	1009.55	98.7003	6	1053.91	98.214
7	1099.23	98.6709	8	1179.26	98.2691
9	1248.68	98.6355	10	1298.82	98.751
11	1344.14	98.5798	12	1443.46	98.6693
13	1513.85	98.5732	14	1608.34	97.8495
15	1658.48	98.3912	16	1748.16	98.9967
17	2307.41	99.2718	18	2357.55	99.2988
19	2382.62	99.3861	20	2926.45	99.1741
21	3370.96	98.8101			

Figure S8. ^{1}H NMR spectrum of compound 2 (600 MHz, pyridine-d_5)

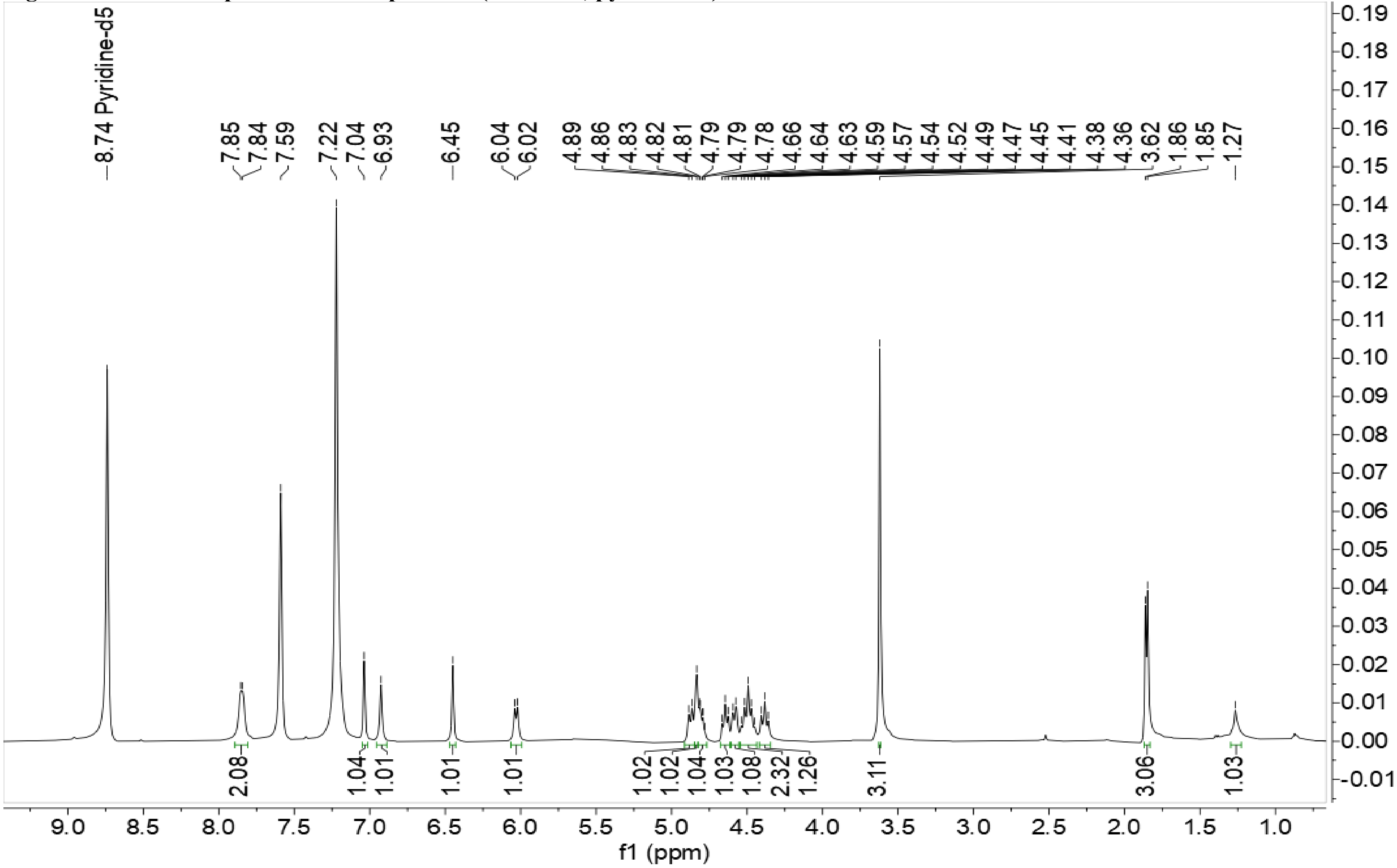

Figure S9. ^{13}C NMR spectrum of compound 2 (150 MHz, pyridine-d_5)

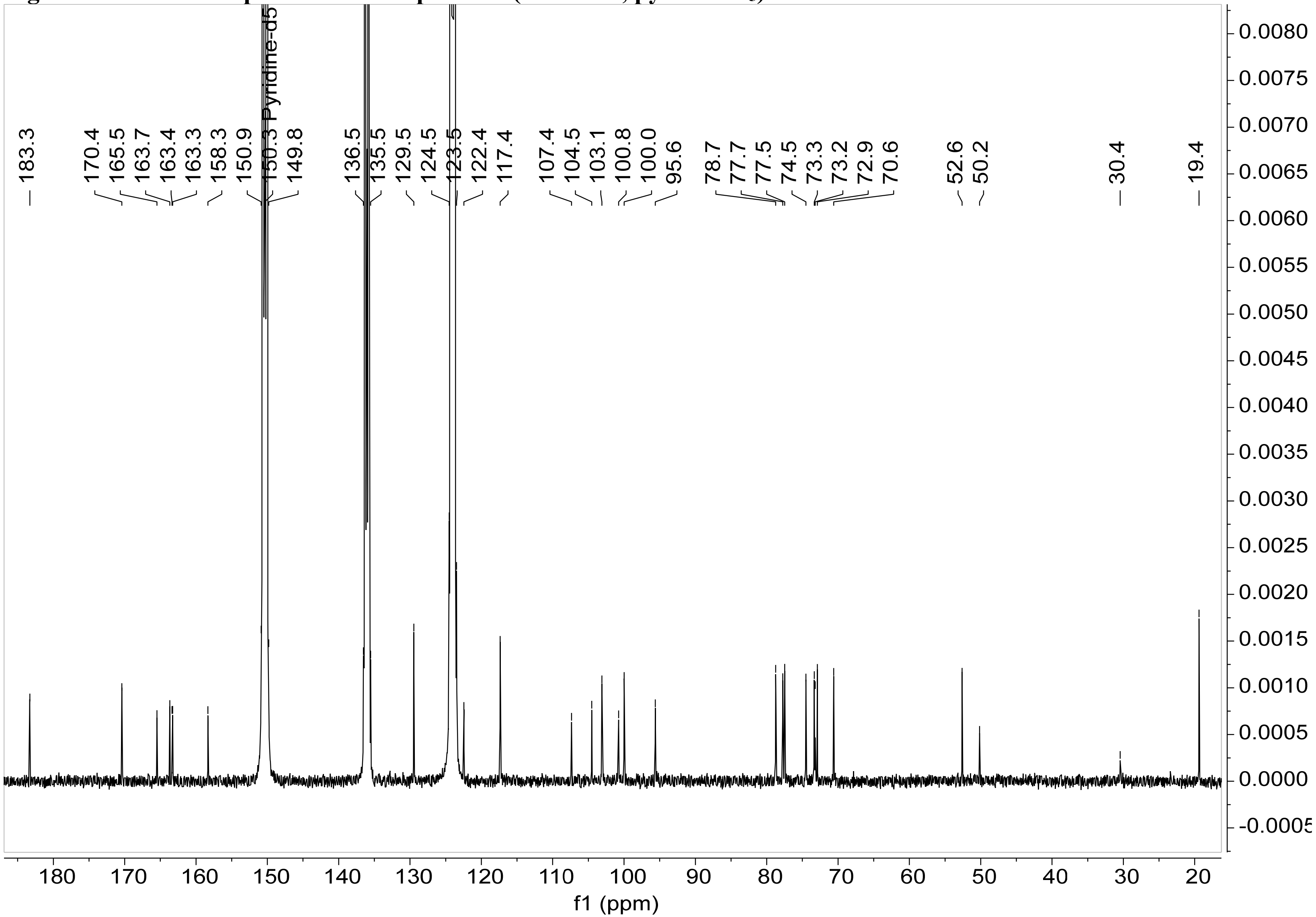

Figure S10. HSQC spectrum of compound 2

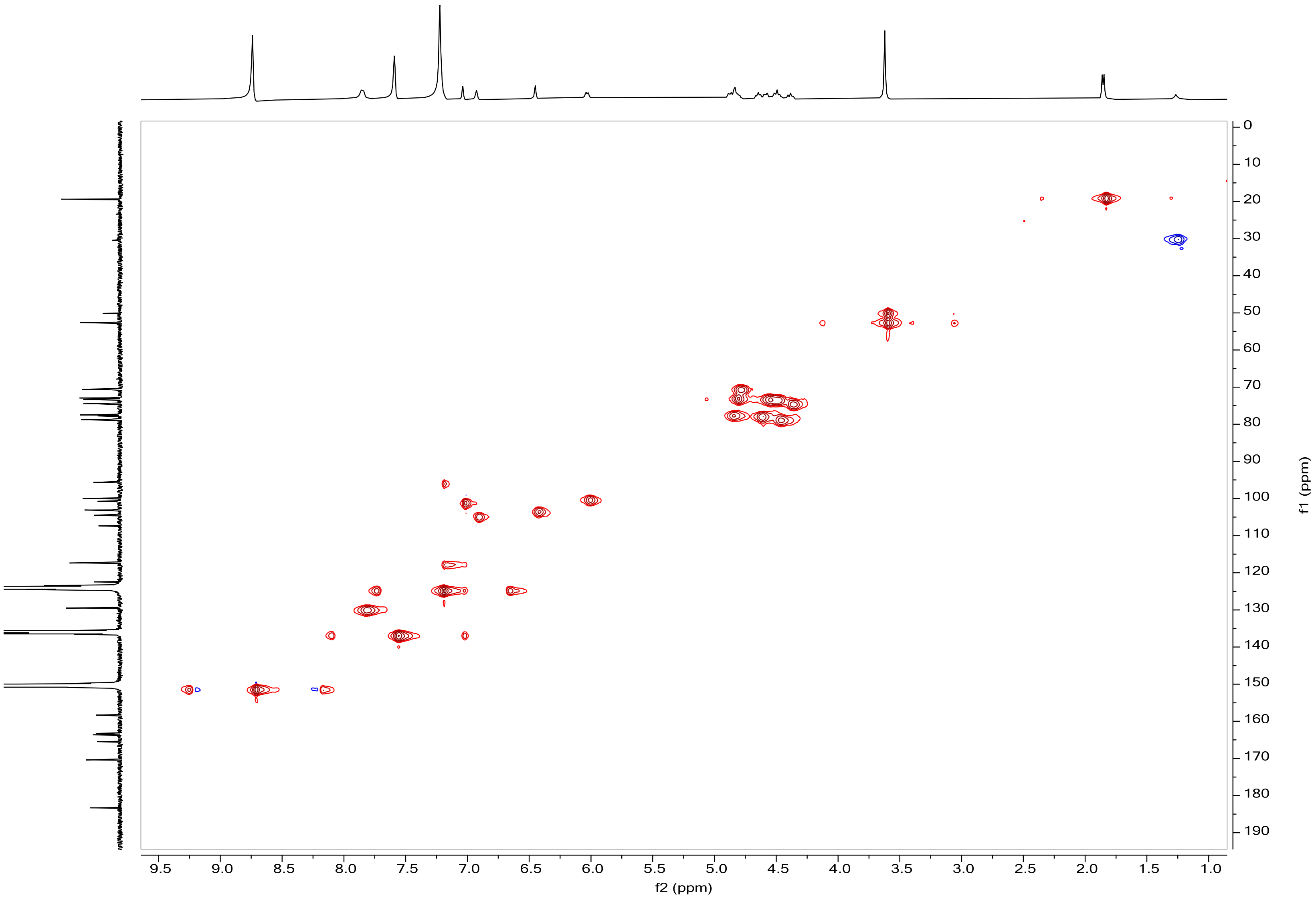

Figure S11. HMBC spectrum of compound 2

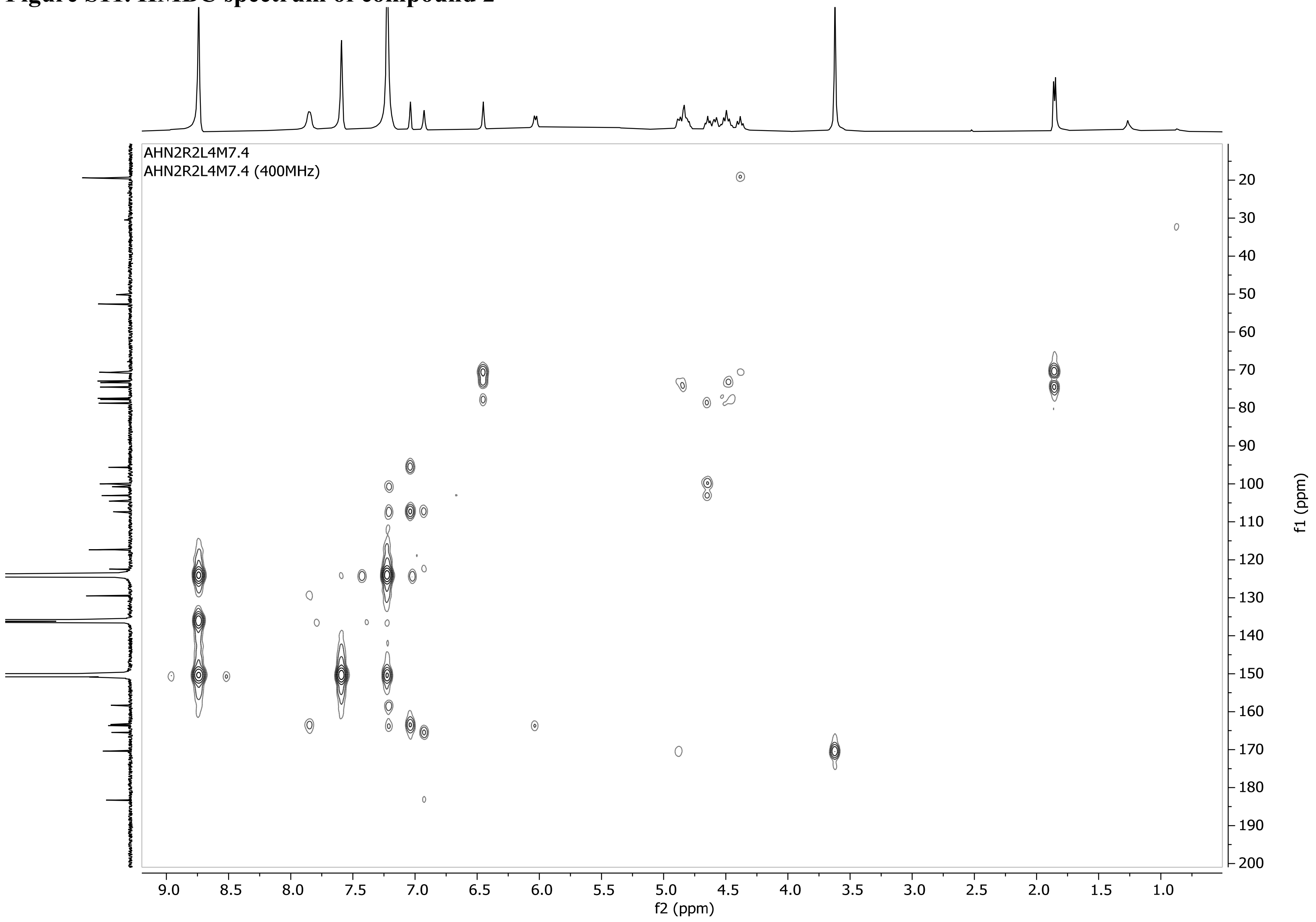

Figure S12. COSY spectrum of compound 2

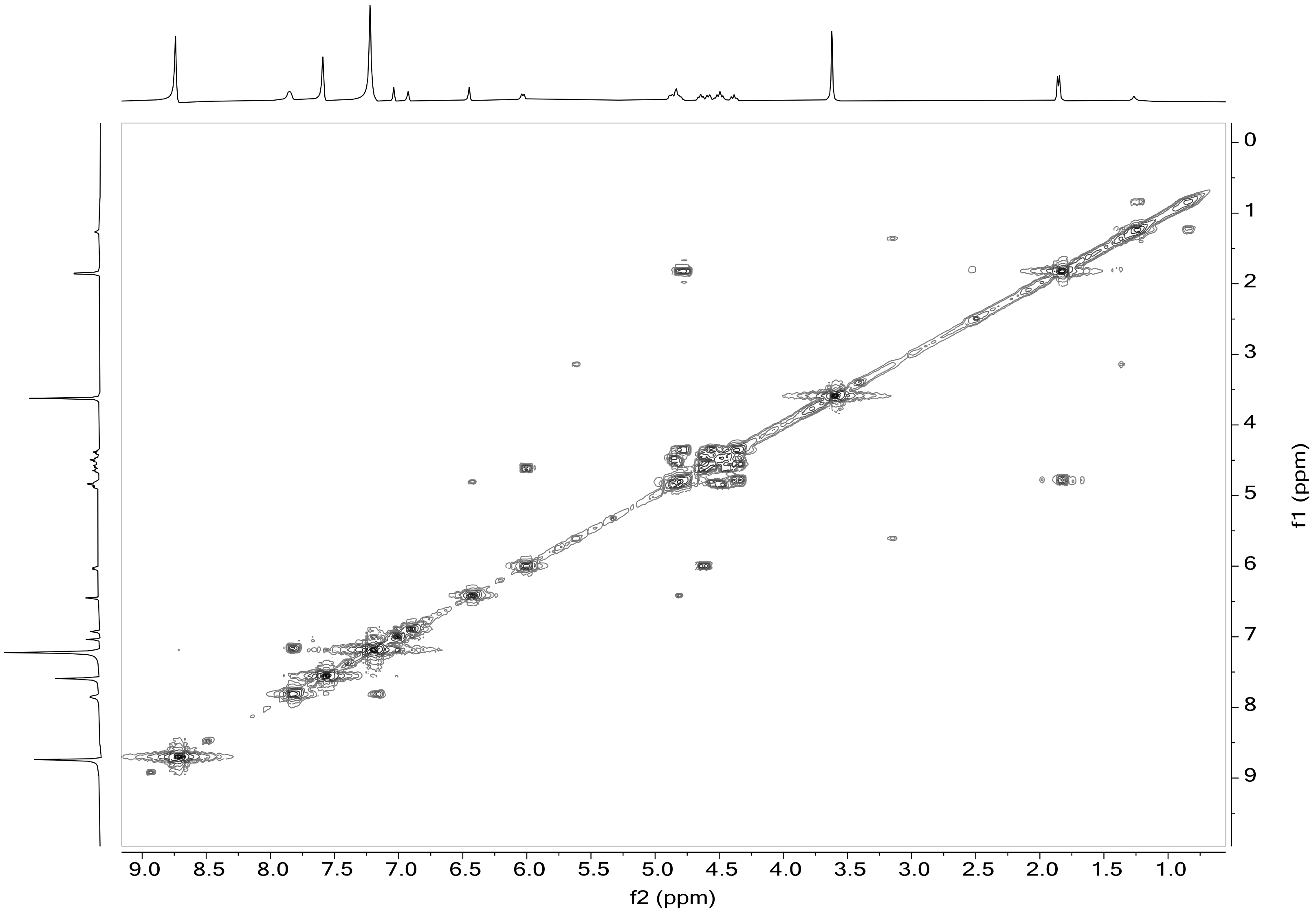

Figure S13. HRESIMS/MS of compound 1

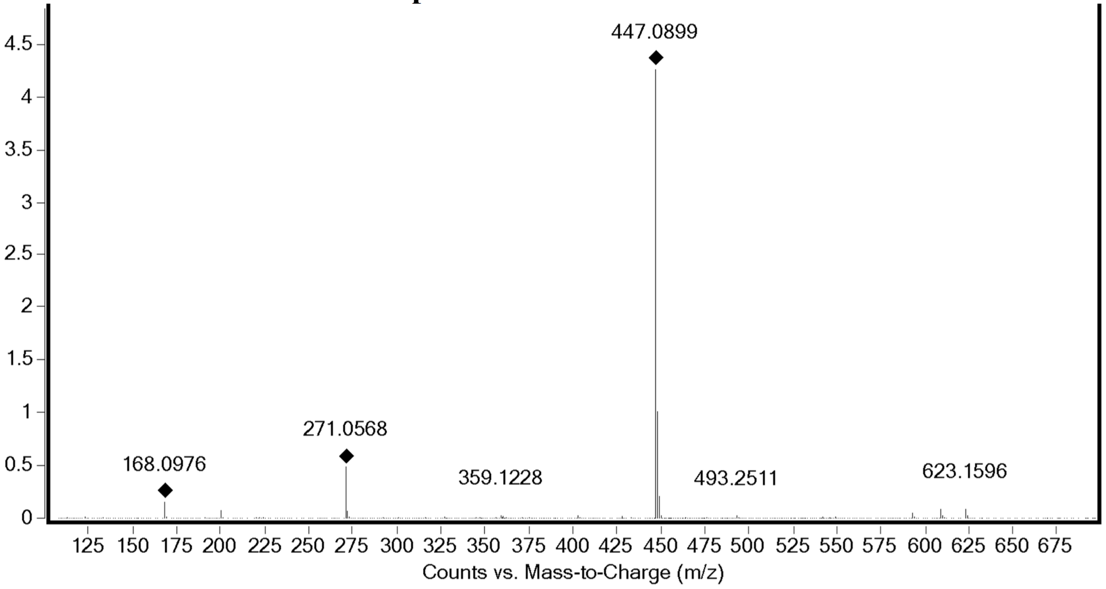

Figure S14. IR(KBr) spectrum of compound 1

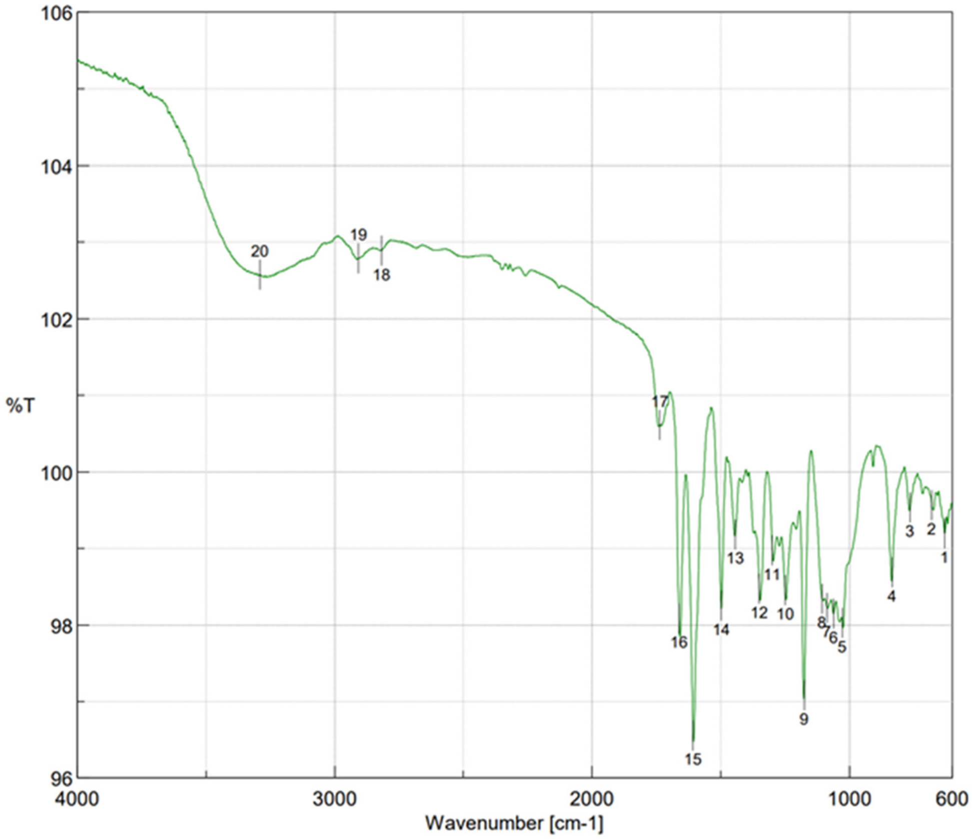

Result of Peak Picking

No.	Position	Intensity	No.	Position	Intensity
1	629.644	99.1941	2	679.785	99.5601
3	763.673	99.5297	4	833.098	98.6825
5	1026.91	98.023	6	1061.62	98.1448
7	1086.69	98.2217	8	1105.98	98.3363
9	1175.4	97.0805	10	1250.61	98.4502
11	1299.79	98.9738	12	1349.93	98.473
13	1443.46	99.1809	14	1498.42	98.2506
15	1607.38	96.5488	16	1662.34	98.0832
17	1736.58	100.606	18	2818.45	102.886
19	2908.13	102.784	20	3289.96	102.568

Figure S15. ^{1}H NMR spectrum of compound 1 (400 MHz, DMSO-d_6)

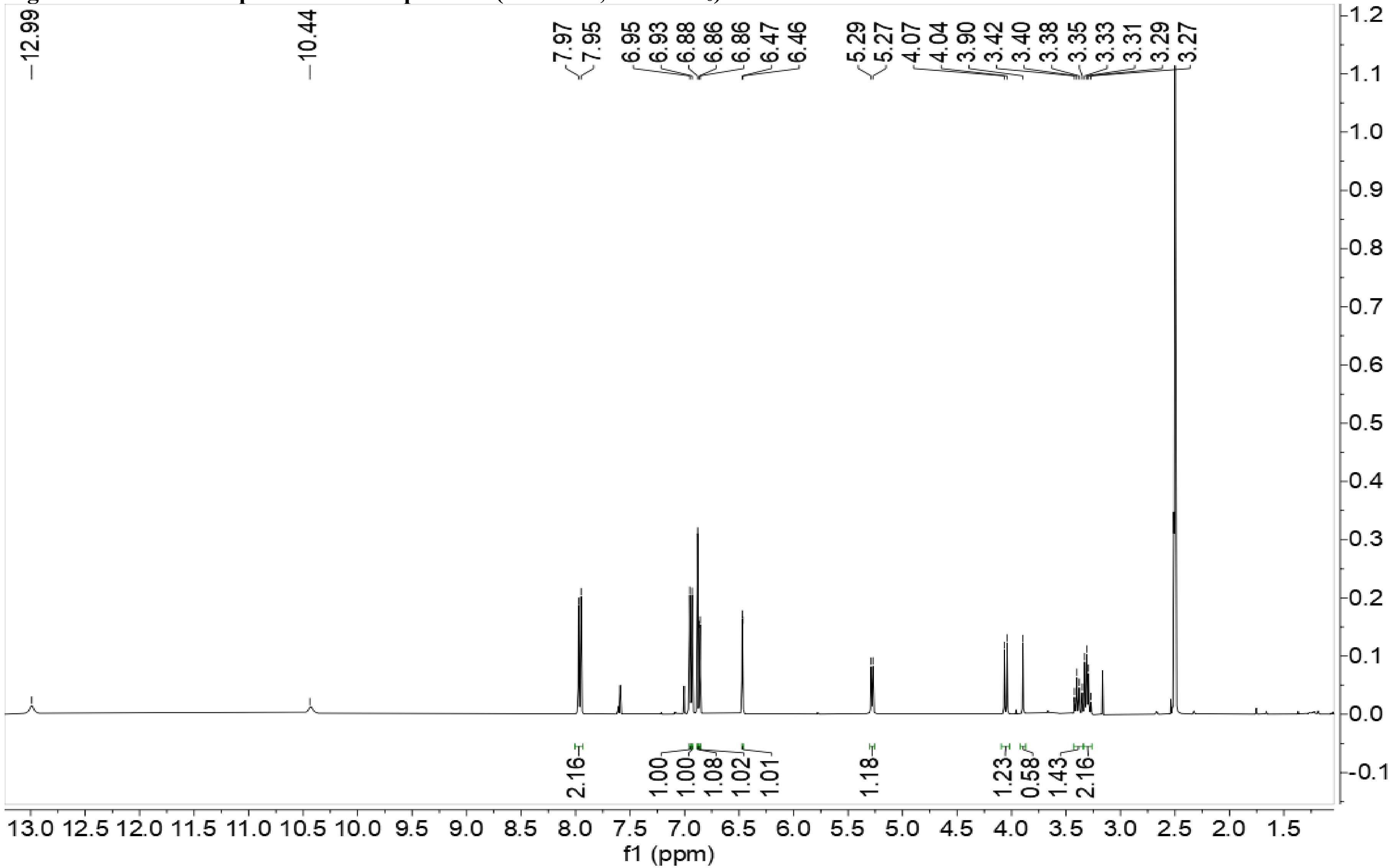

Figure S16. HRESIMS/MS of compound 3

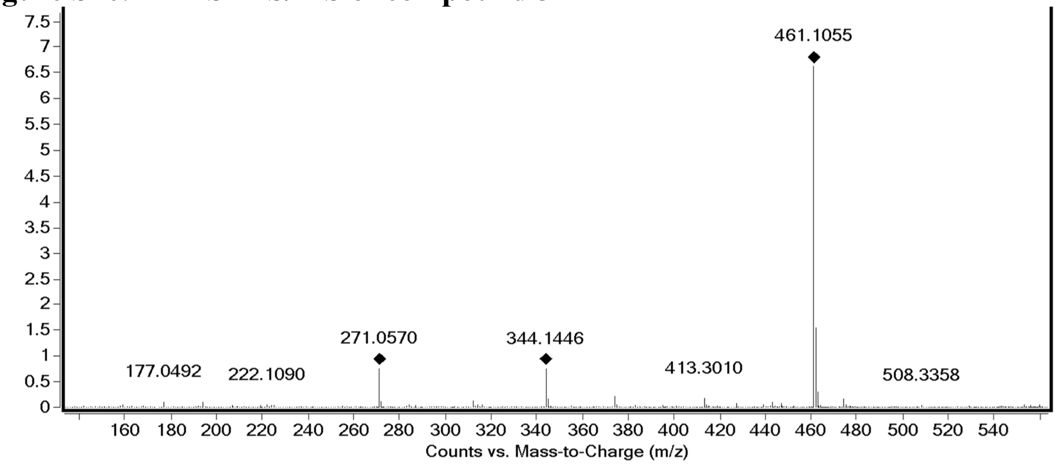

Figure S17. IR(KBr) spectrum of compound 3

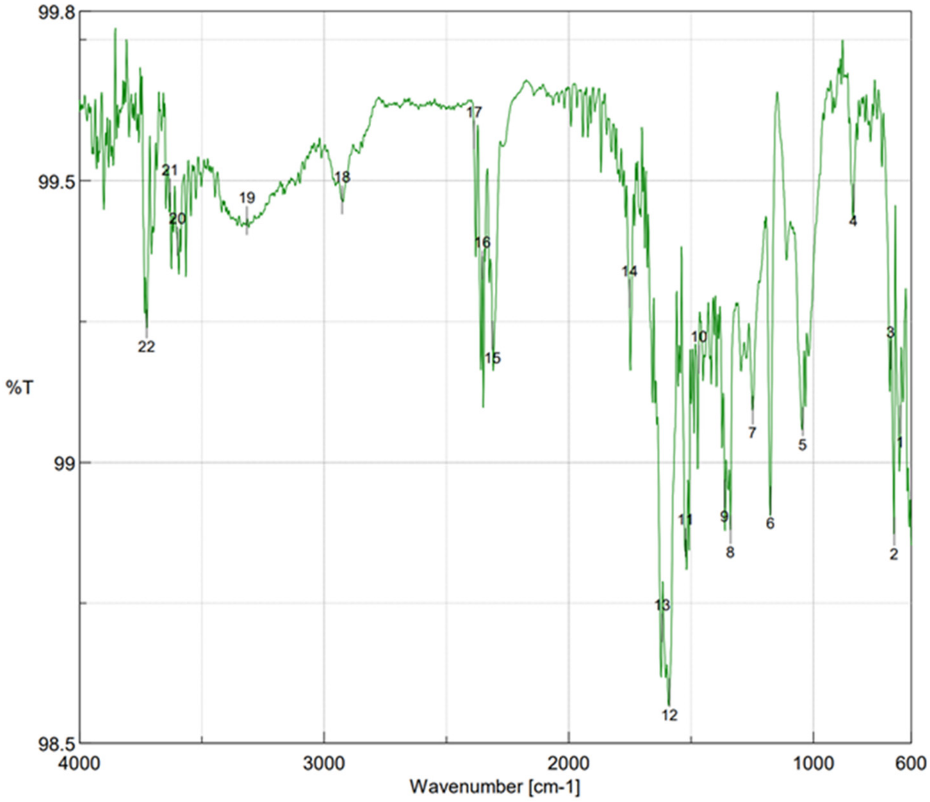

Result of Peak Picking

No.	Position	Intensity	No.	Position	Intensity
1	645.072	99.0755	2	670.142	98.8769
3	684.606	99.1894	4	835.026	99.4685
5	1044.26	99.0713	6	1174.44	98.9313
7	1248.68	99.0917	8	1339.32	98.8796
9	1363.43	98.9431	10	1468.53	99.1818
11	1523.49	98.8574	12	1588.09	98.5904
13	1618.95	98.7055	14	1752.98	99.2987
15	2312.23	99.2245	16	2352.73	99.3492
17	2387.44	99.5805	18	2926.45	99.4652
19	3316	99.4287	20	3600.45	99.3916
21	3630.34	99.4783	22	3725.8	99.2451

Figure S18. **¹H NMR spectrum of compound 3 (600 MHz, DMSO-d_6)**

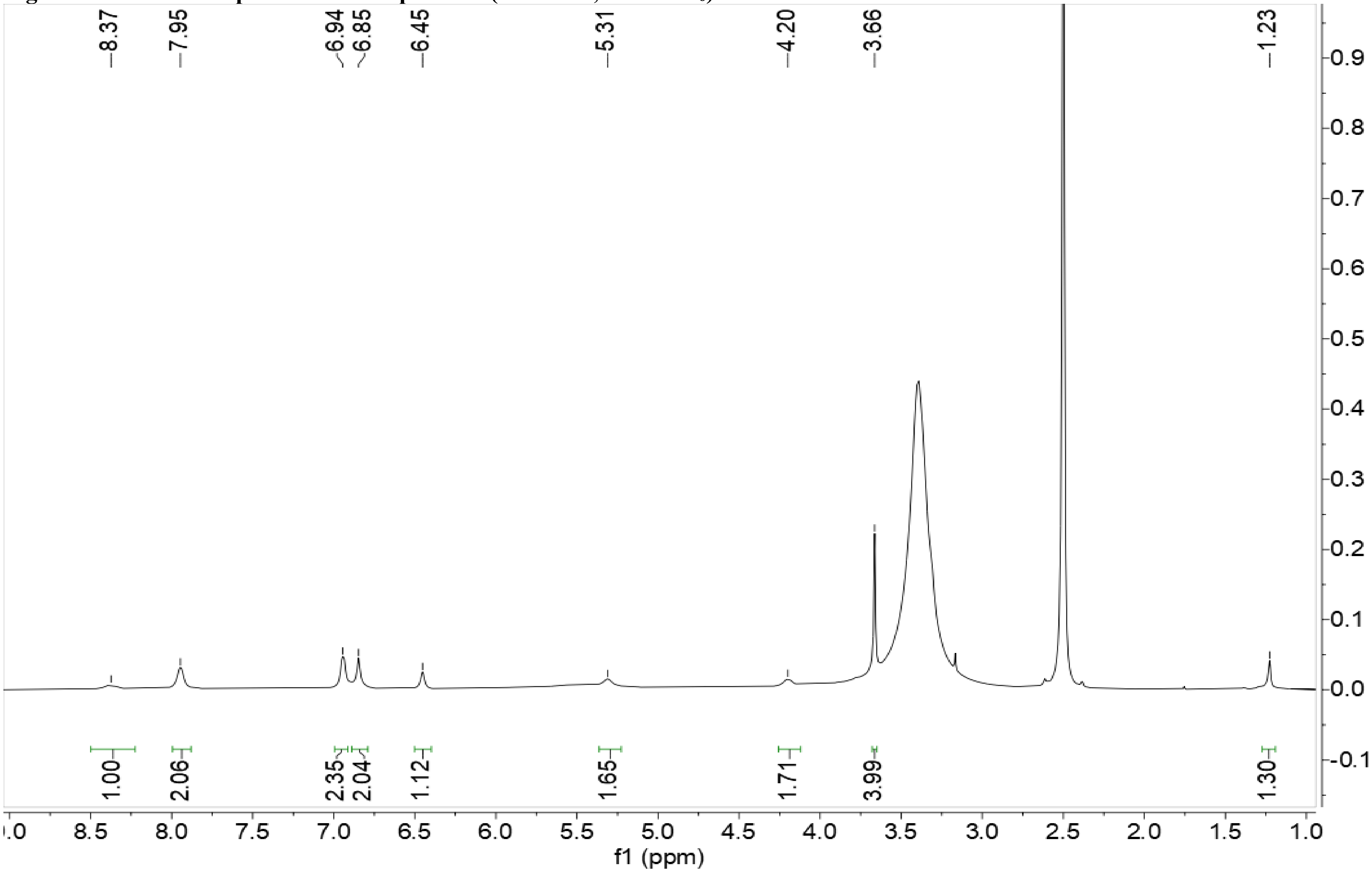

Figure S19. ^{13}**C NMR spectrum of compound 3 (150 MHz, DMSO-*d*$_6$)**

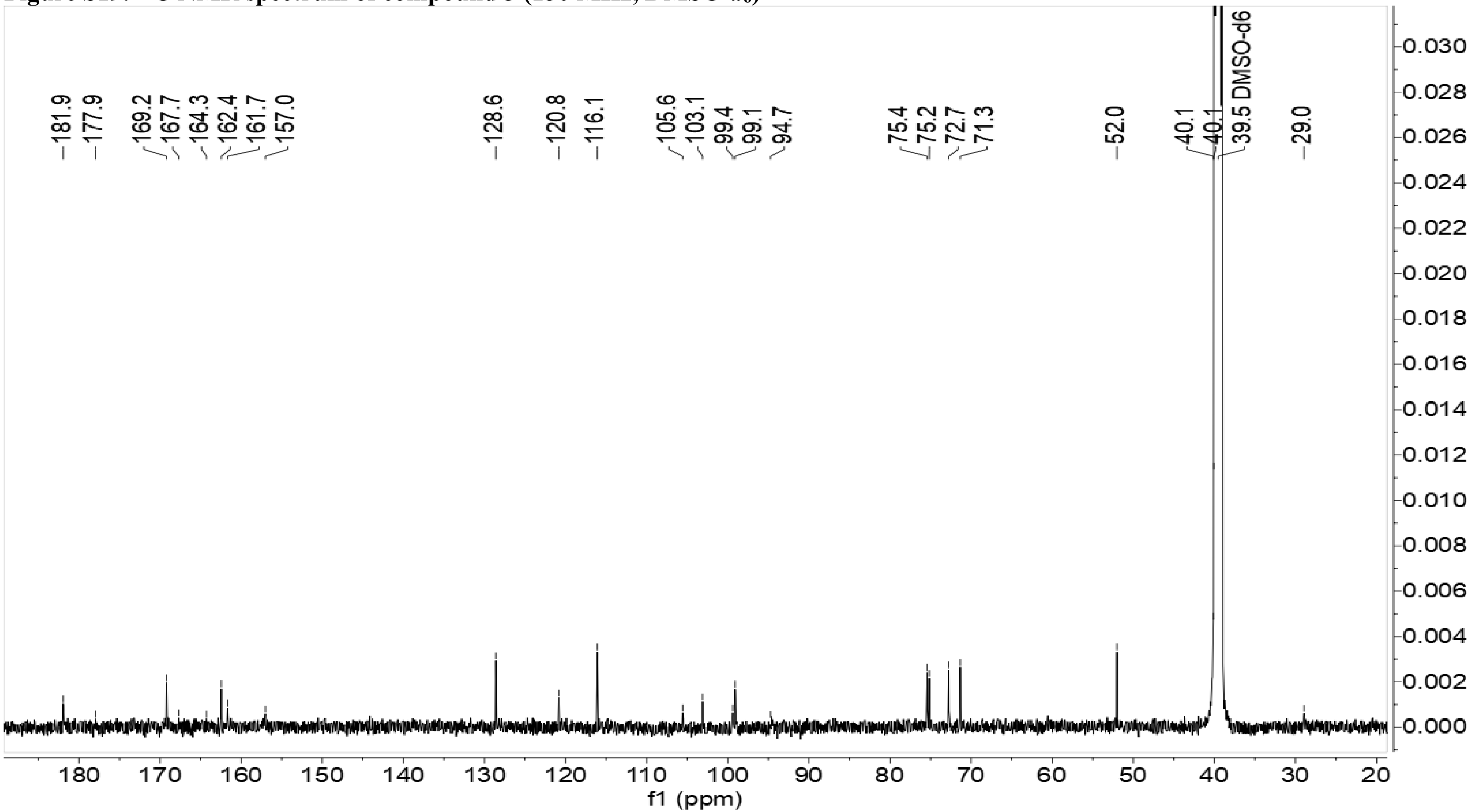

Figure S20. HRESIMS data of compound 4

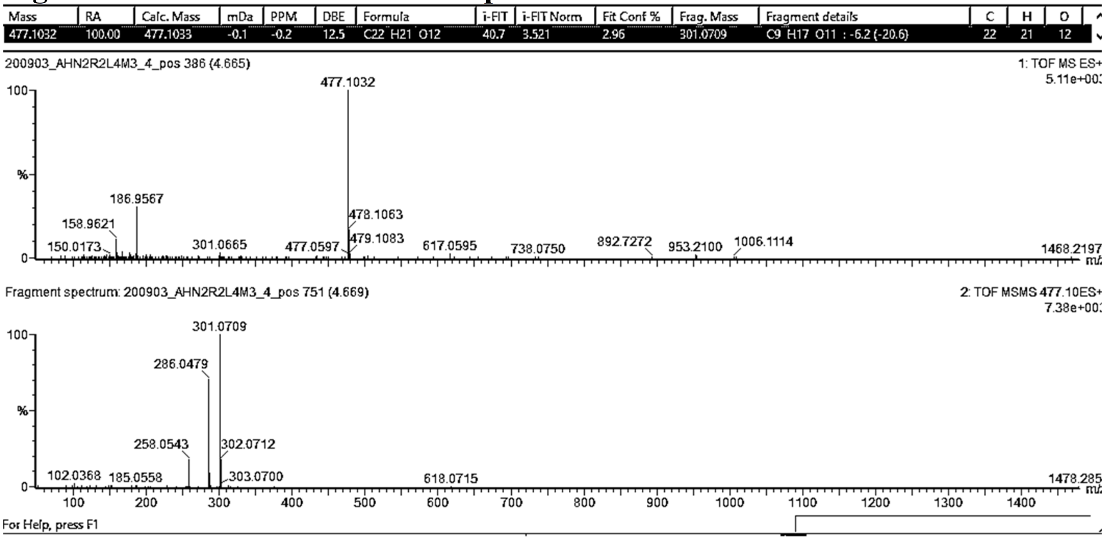

Figure S21. UV spectrum of compound 4

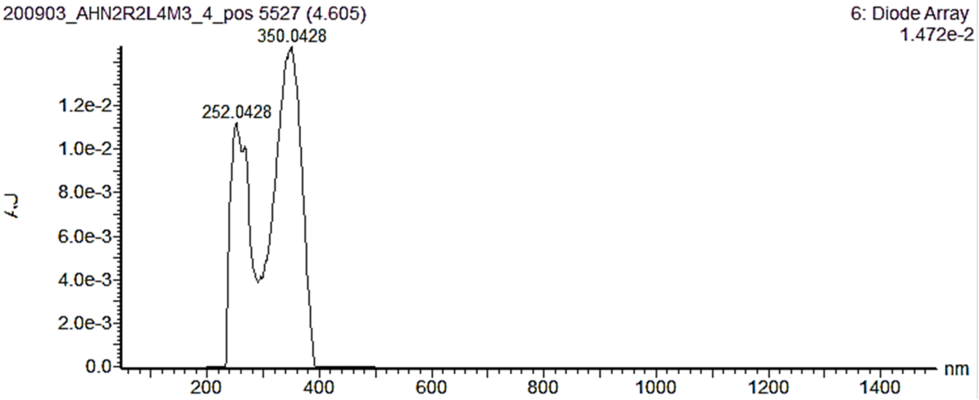

Figure S22. IR(KBr) spectrum of compound 4

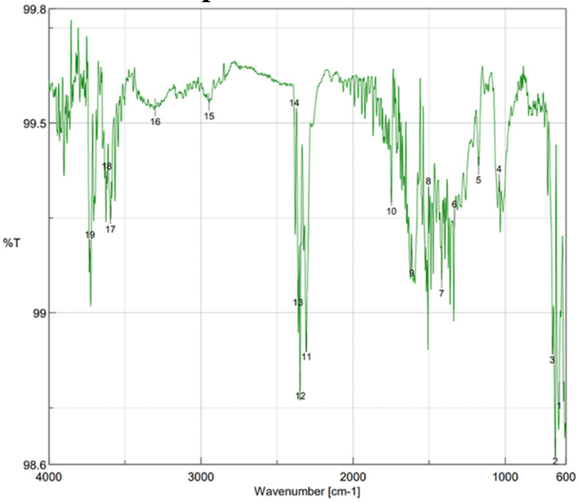

Result of Peak Picking

No.	Position	Intensity	No.	Position	Intensity
1	645.072	98.7928	2	670.142	98.6457
3	689.427	98.9129	4	1039.44	99.341
5	1174.44	99.386	6	1333.53	99.2486
7	1418.39	99.0883	8	1503.24	99.3084
9	1613.16	99.1413	10	1748.16	99.3041
11	2307.41	98.9216	12	2347.91	98.8183
13	2362.37	99.0645	14	2387.44	99.5148
15	2946.7	99.5546	16	3300.57	99.5406
17	3595.63	99.2568	18	3620.7	99.3485
19	3730.62	99.1677			

Figure S23. ^{1}H NMR spectrum of compound 4 (500MHz, DMSO-d_6)

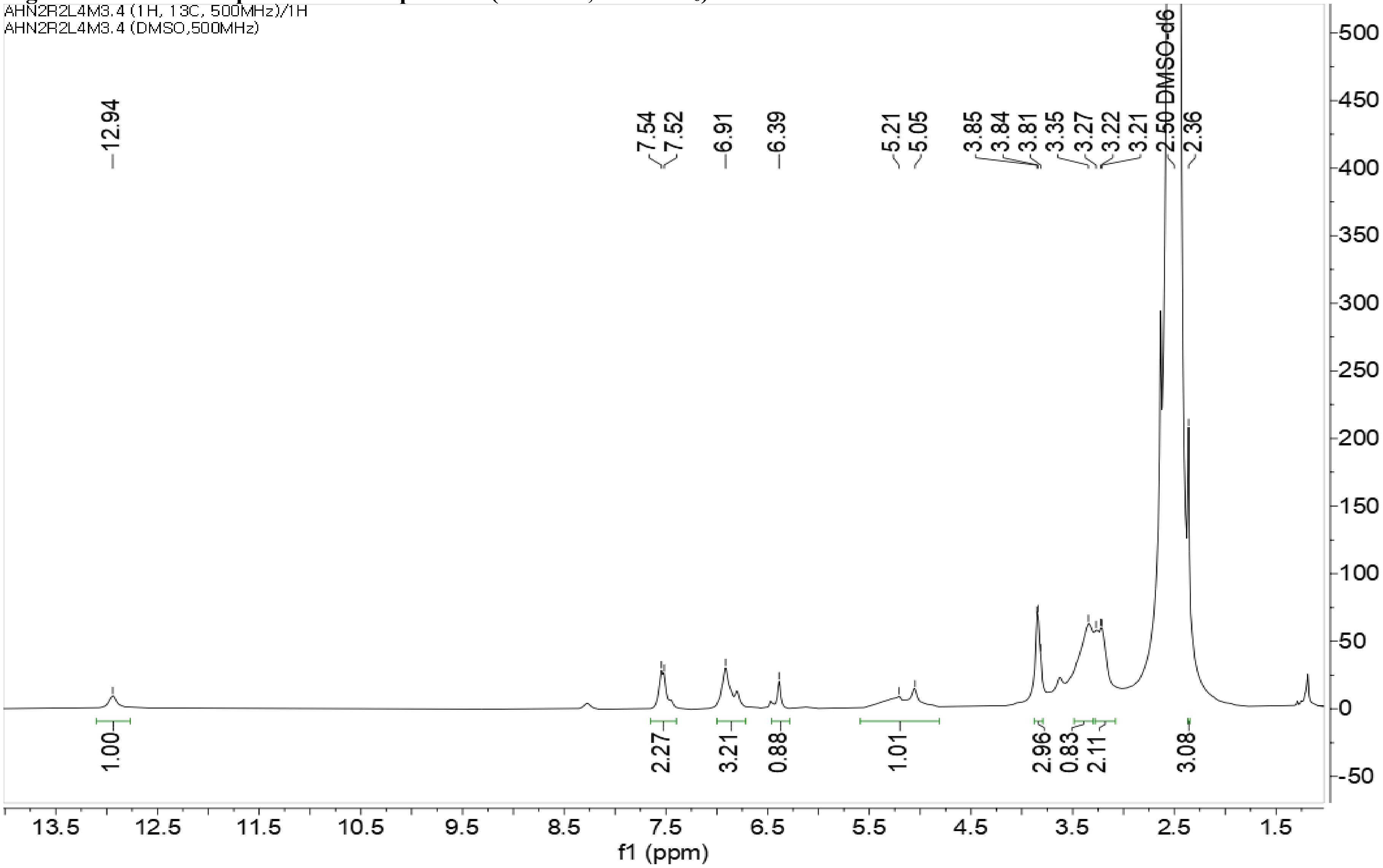

Figure S24. ^{13}C **NMR spectrum of compound 4 (125 MHz, DMSO-d_6)**

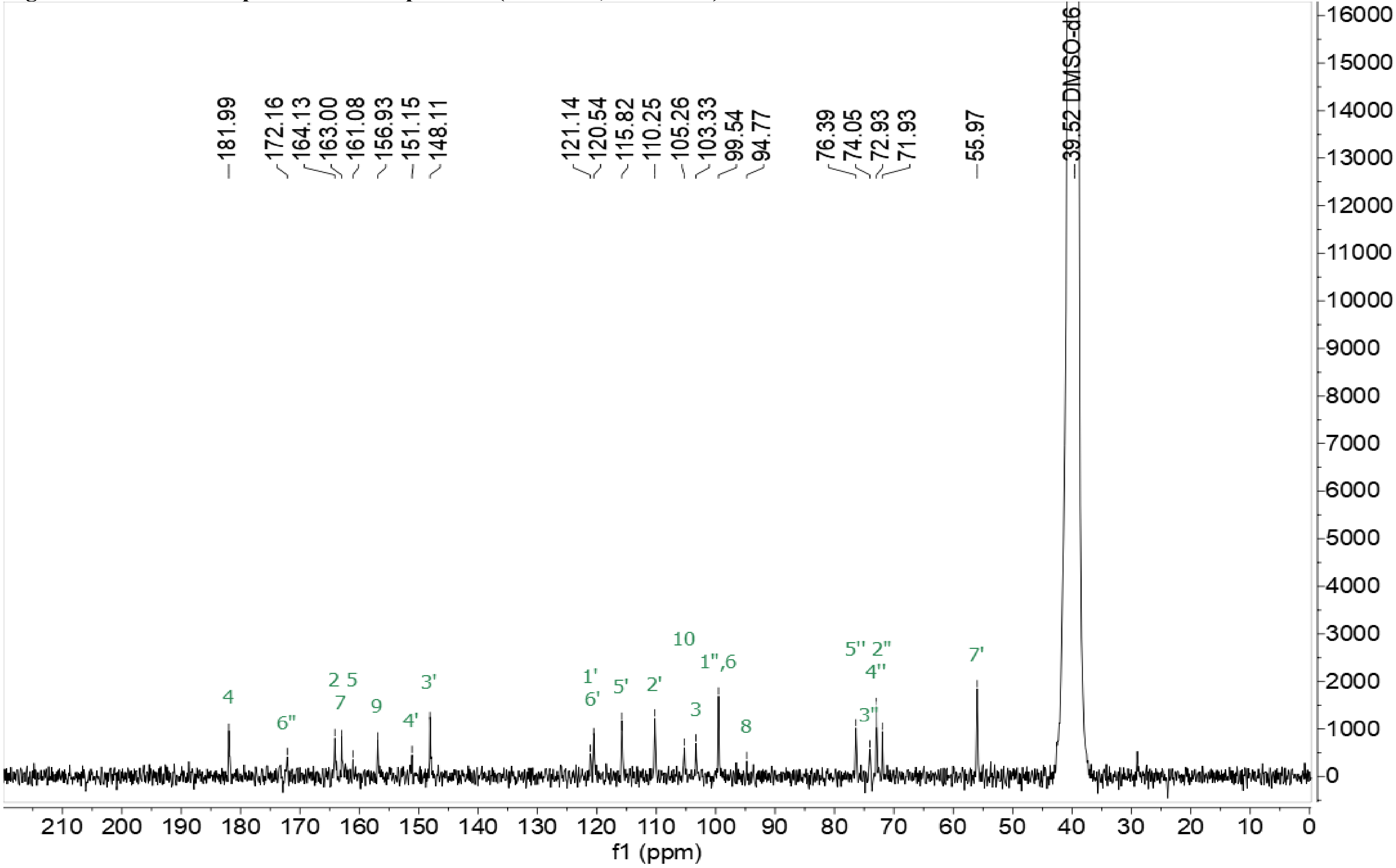

Figure S25. HRESIMS data of compound 5

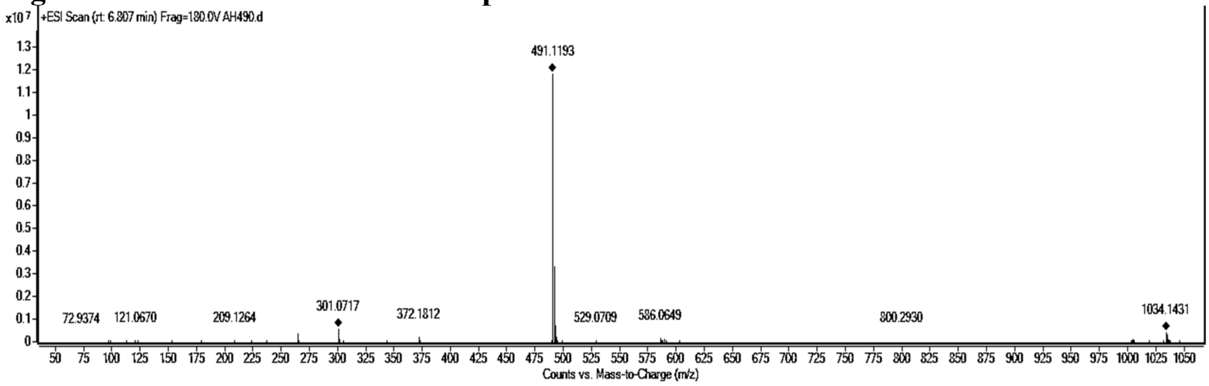

Figure S26. UV spectrum of compound 5

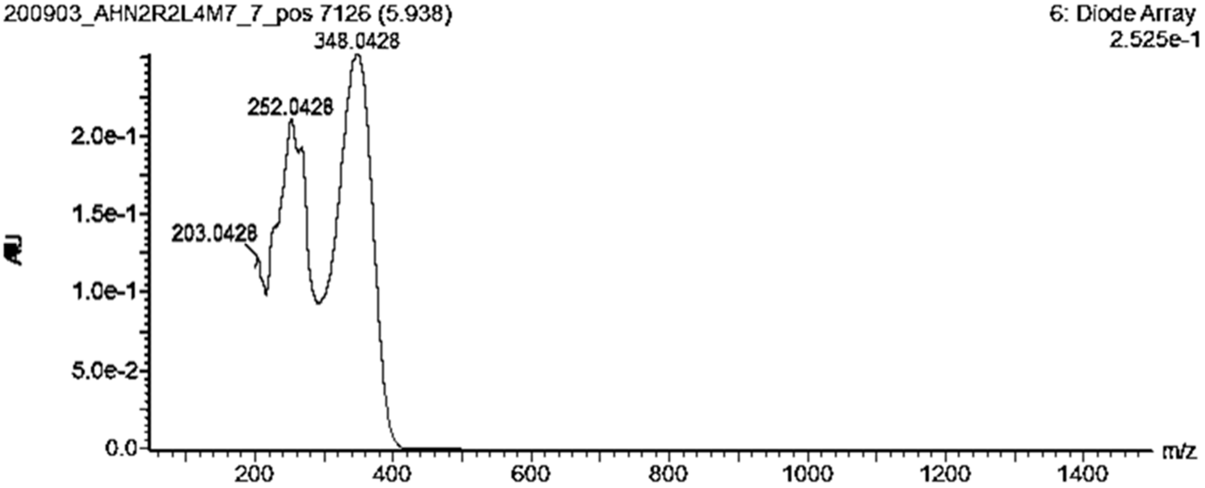

Figure S27. IR(KBr) spectrum of compound 5

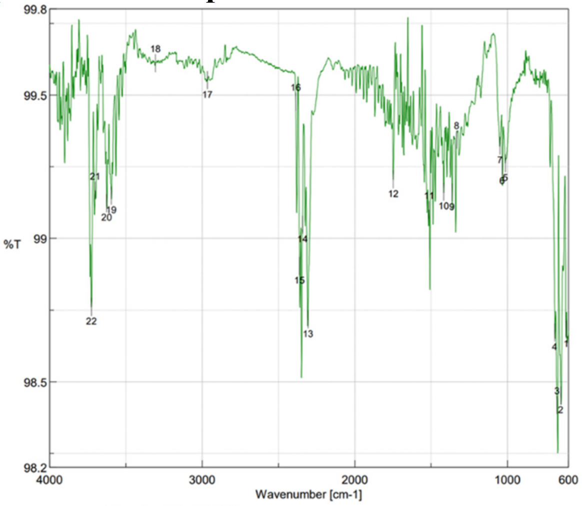

Result of Peak Picking

No.	Position	Intensity	No.	Position	Intensity
1	615.181	98.6816	2	649.893	98.4508
3	674.963	98.4177	4	689.427	98.6703
5	1014.37	99.2617	6	1034.62	99.2499
7	1049.09	99.3224	8	1333.53	99.3439
9	1363.43	99.1592	10	1418.39	99.1616
11	1513.85	99.0986	12	1748.16	99.2056
13	2307.41	98.7165	14	2342.12	99.0468
15	2362.37	98.9032	16	2387.44	99.4763
17	2966.95	99.5515	18	3306.36	99.6102
19	3595.63	99.1479	20	3625.52	99.1219
21	3700.73	99.1664	22	3725.8	98.7599

Figure S28. **^{1}H NMR spectrum of compound 5 (600 MHz, DMSO-d_6)**

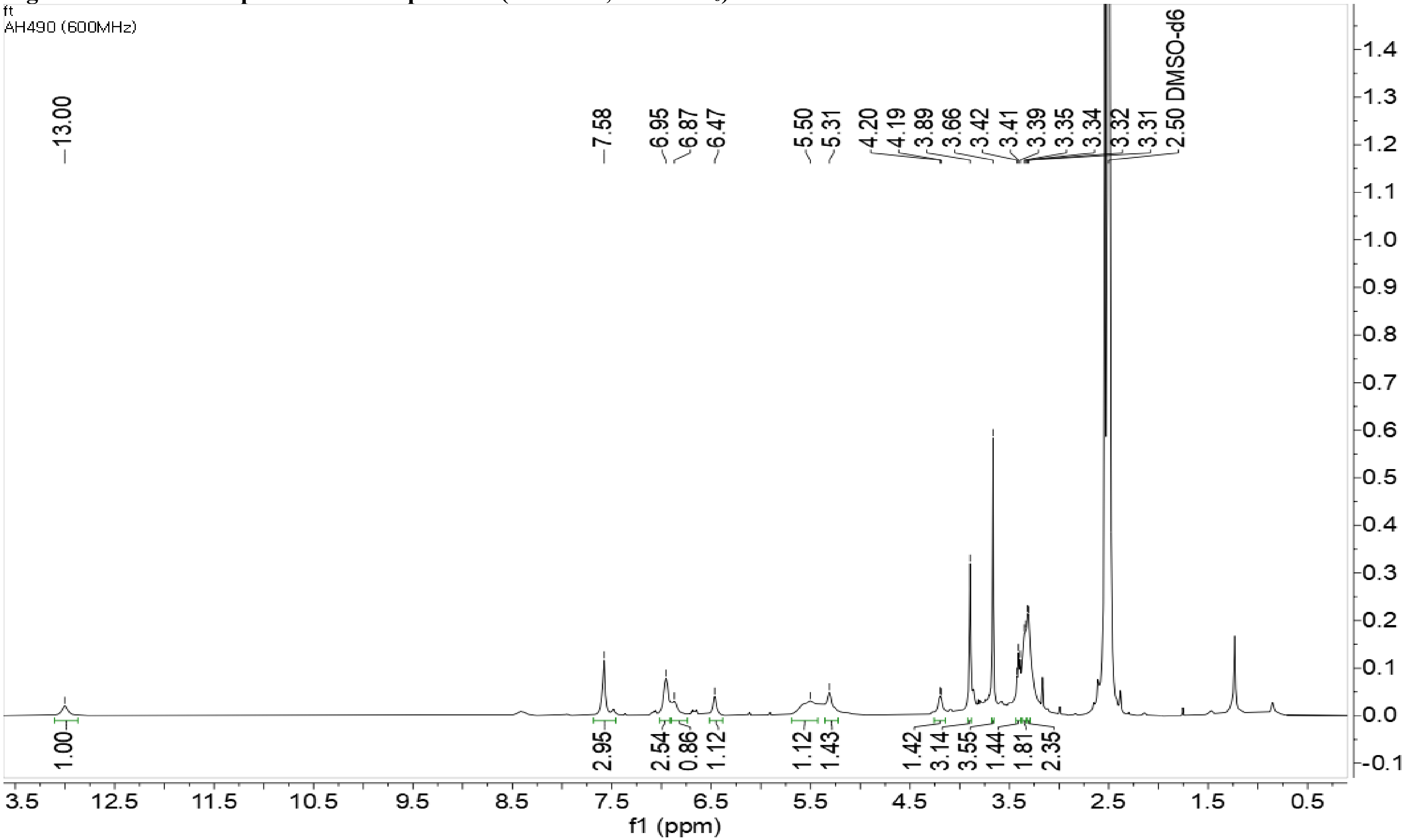

Figure S29. ^{13}**C NMR spectrum of compound 5 (150 MHz, DMSO-*d*$_6$)**

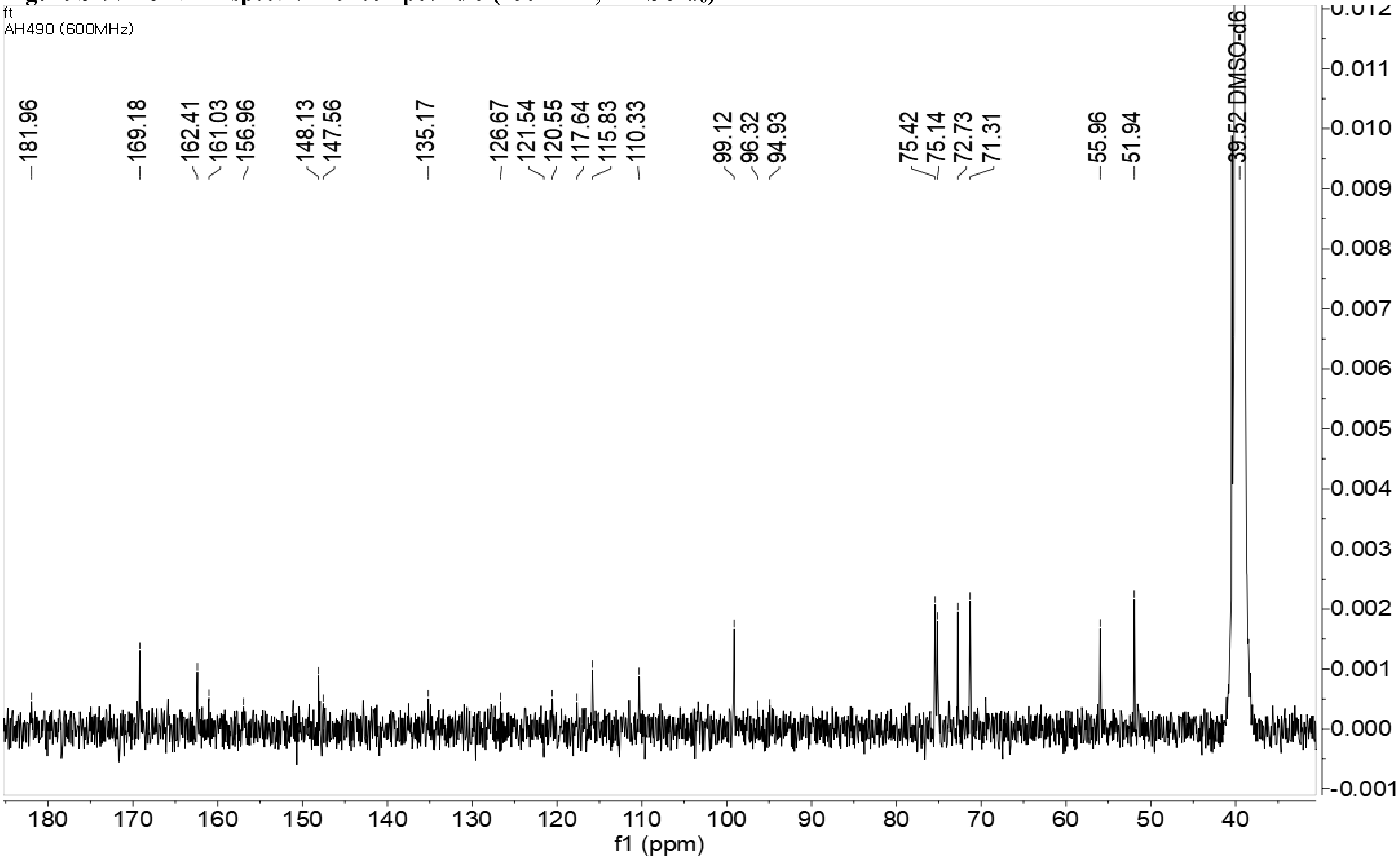

Figure S30. HRESIMS data of compound 6

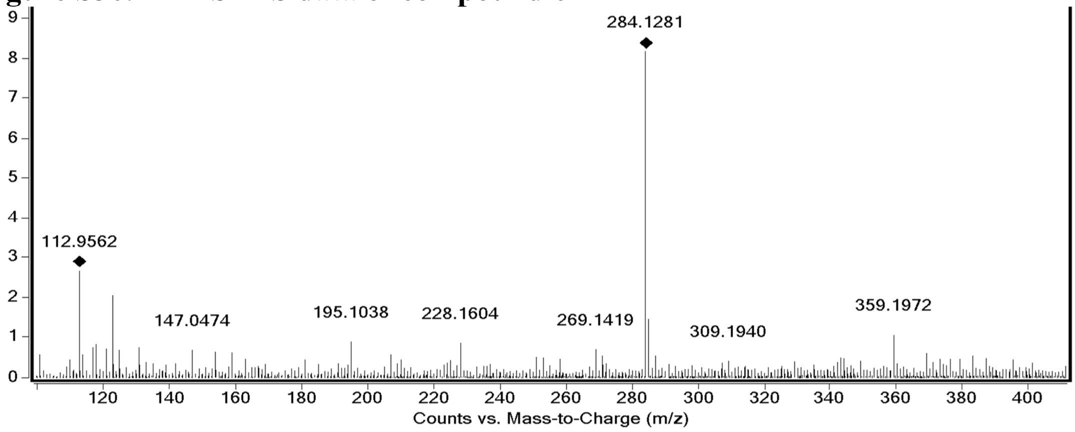

Figure S31. IR(KBr) spectrum of compound 6

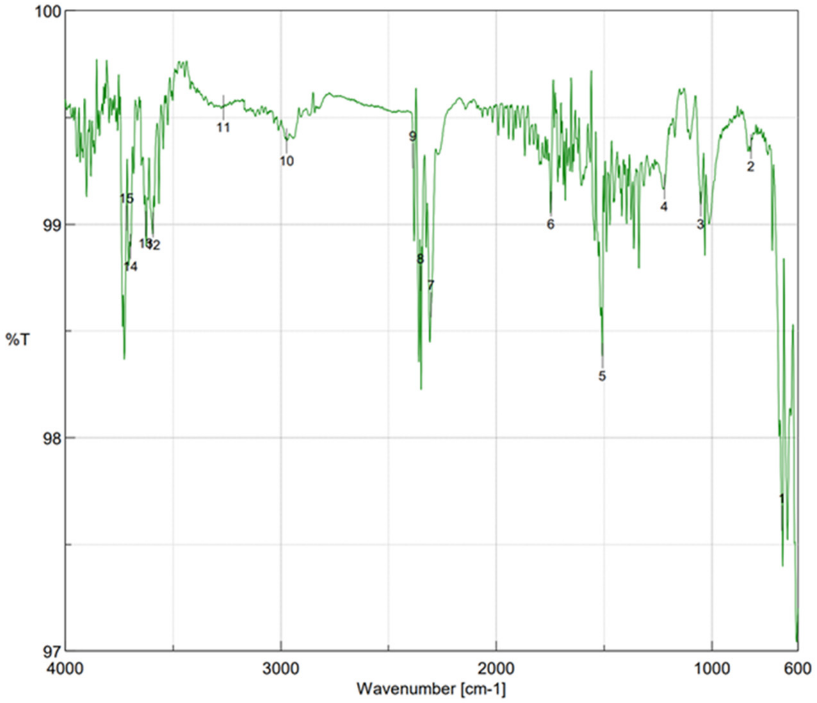

Result of Peak Picking

No.	Position	Intensity	No.	Position	Intensity
1	673.999	97.6227	2	818.634	99.3625
3	1051.98	99.0938	4	1220.72	99.1741
5	1508.06	98.3826	6	1746.23	99.0941
7	2302.59	98.6215	8	2351.77	98.7456
9	2386.48	99.3191	10	2972.73	99.3889
11	3265.86	99.5452	12	3592.73	98.9952
13	3627.45	99.0012	14	3696.87	98.8935
15	3717.12	99.0272			

Figure S32. **^{1}H NMR spectrum of compound 6 (500 MHz, DMSO-d_6)**

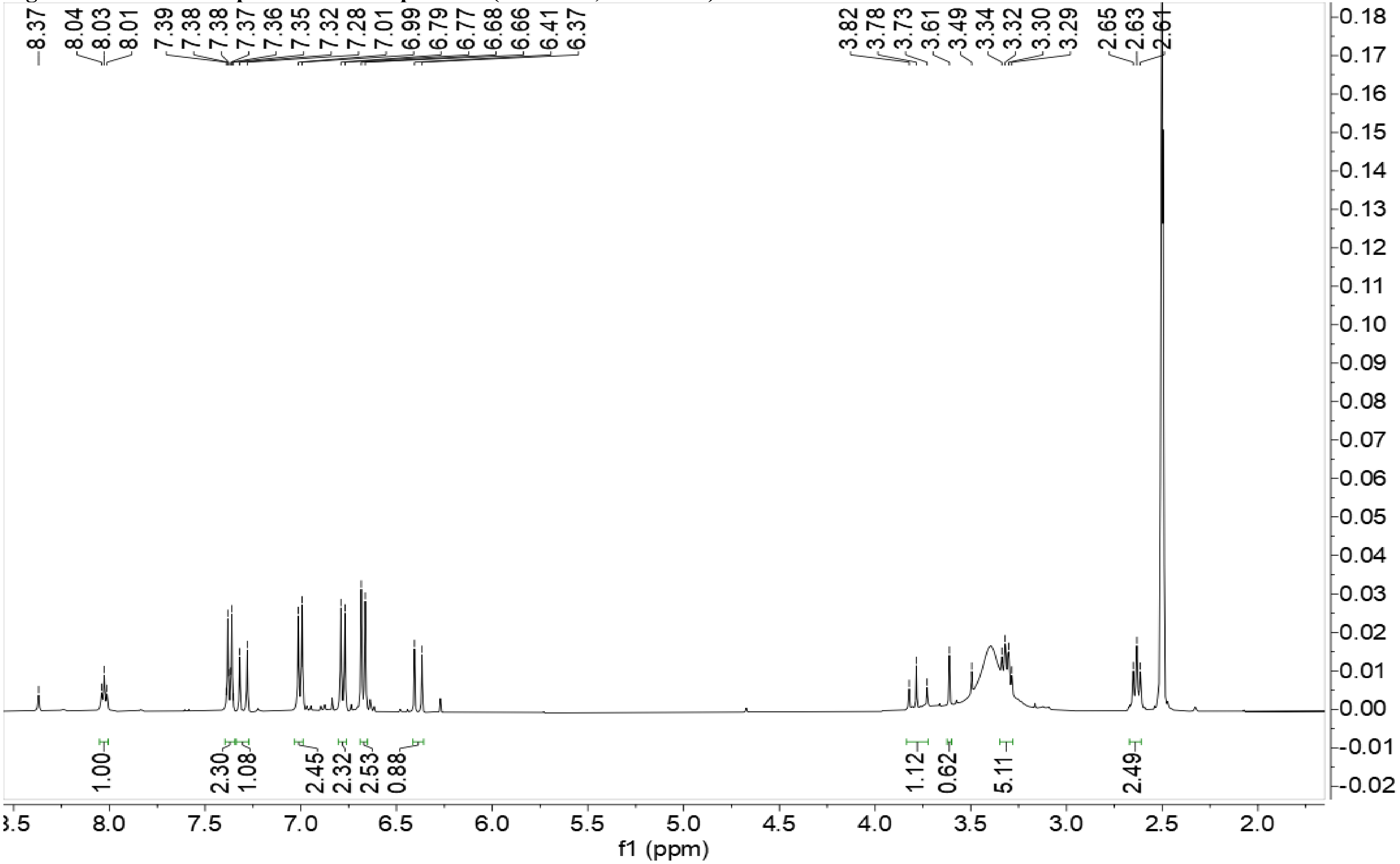

Figure S33. ^{13}**C NMR spectrum of compound 6 (125 MHz, DMSO-*d*$_6$)**

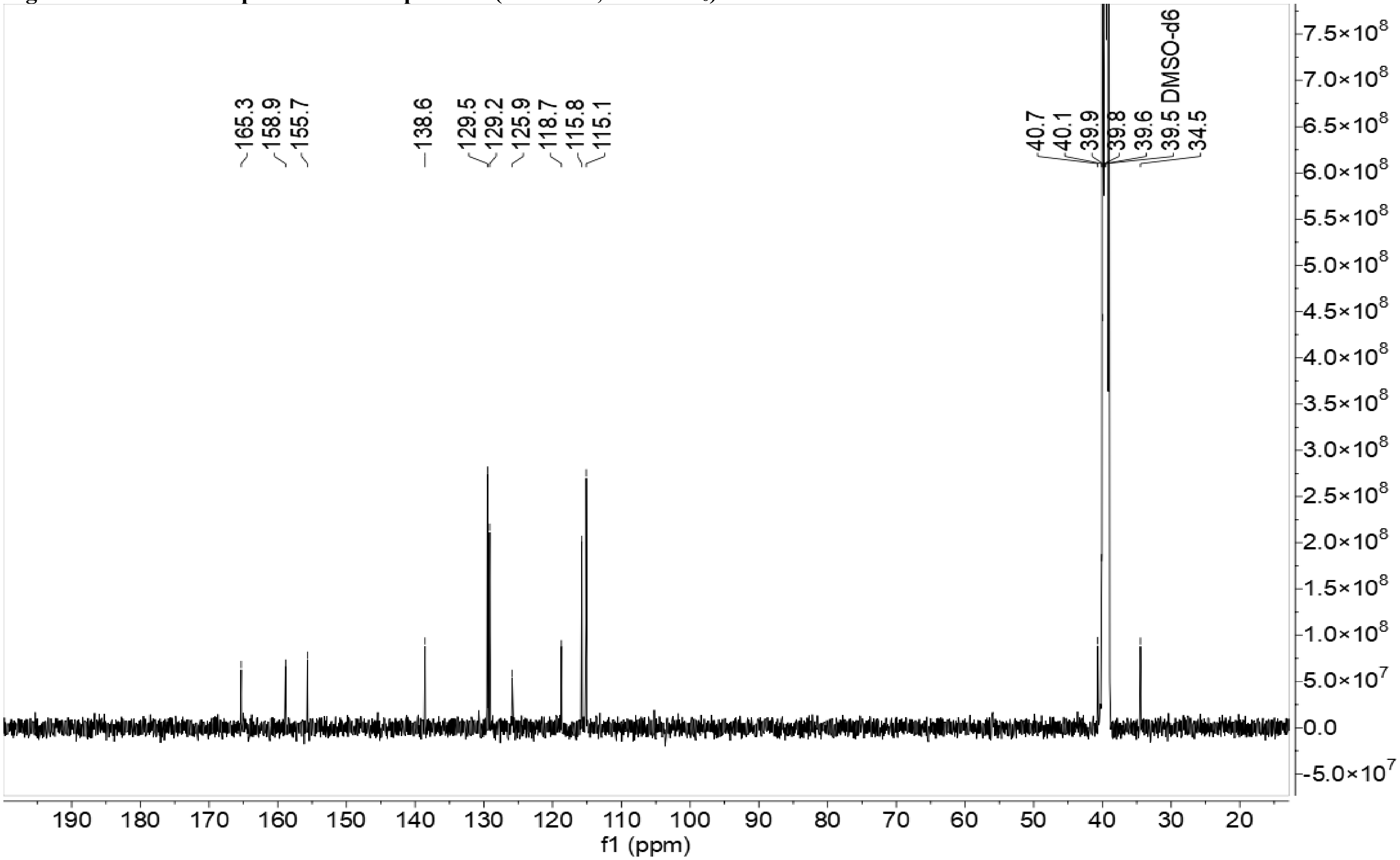

Figure S34. HRESIMS data of compound 7

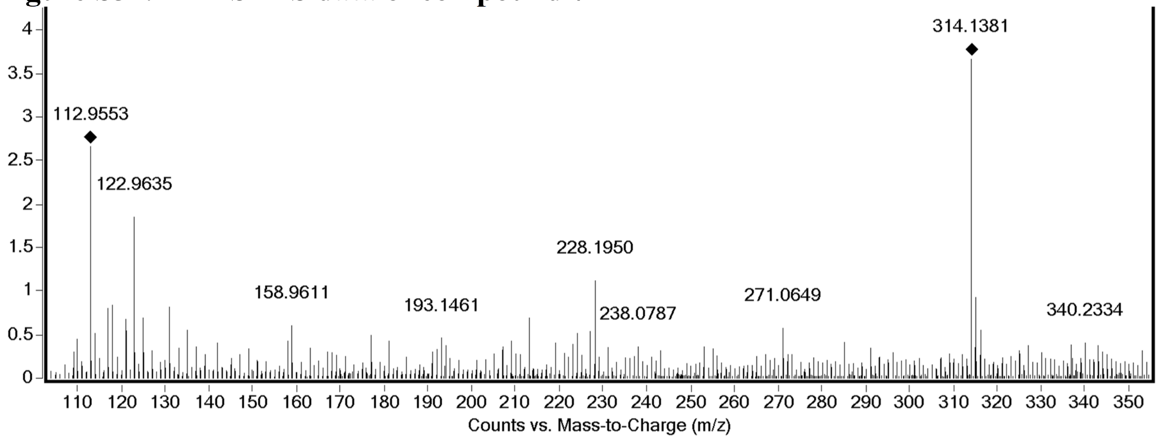

Figure S35. IR(KBr) spectrum of compound 7

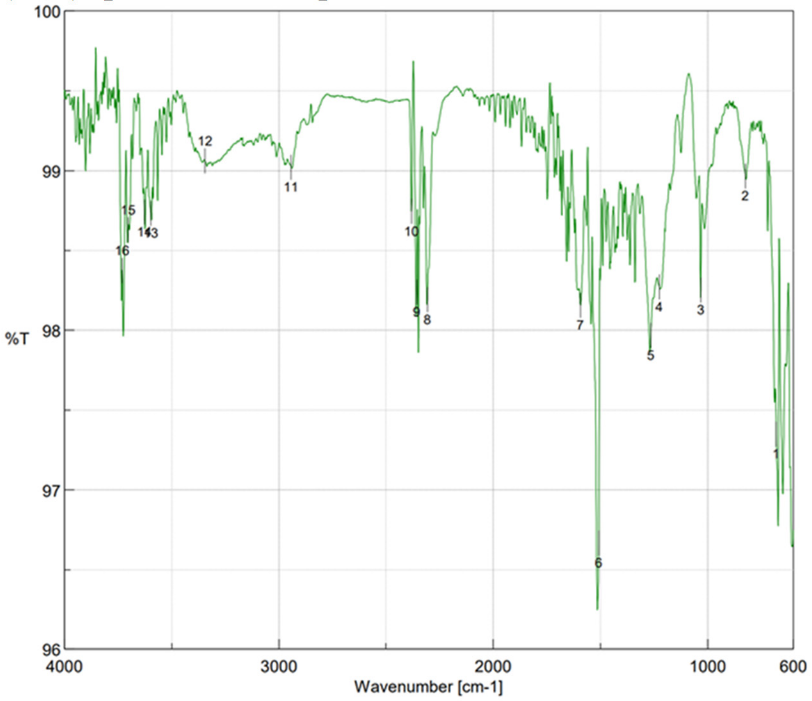

Result of Peak Picking

No.	Position	Intensity	No.	Position	Intensity
1	679.785	97.3464	2	823.455	98.9638
3	1031.73	98.2506	4	1225.54	98.27
5	1265.07	97.965	6	1508.06	96.6639
7	1592.91	98.1548	8	2307.41	98.1905
9	2357.55	98.2369	10	2381.66	98.7437
11	2942.84	99.0206	12	3344.93	99.0604
13	3597.56	98.7302	14	3627.45	98.7395
15	3702.66	98.6277	16	3731.58	98.3732

Figure S36. ^{1}H NMR spectrum of compound 7 (400 MHz, DMSO-d_6)

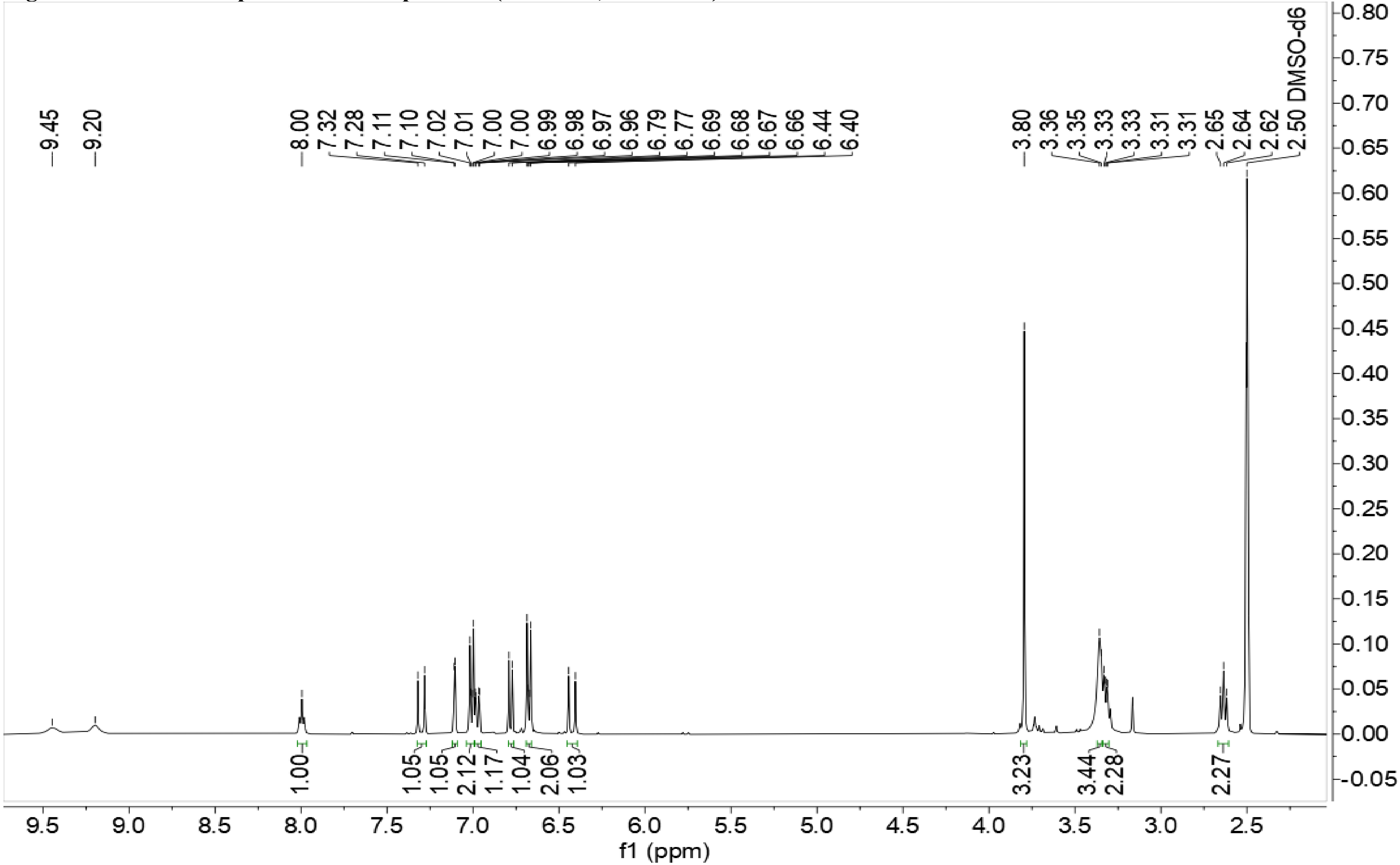

Figure S37. ^{13}C NMR spectrum of compound 7 (100 MHz, DMSO-d_6)

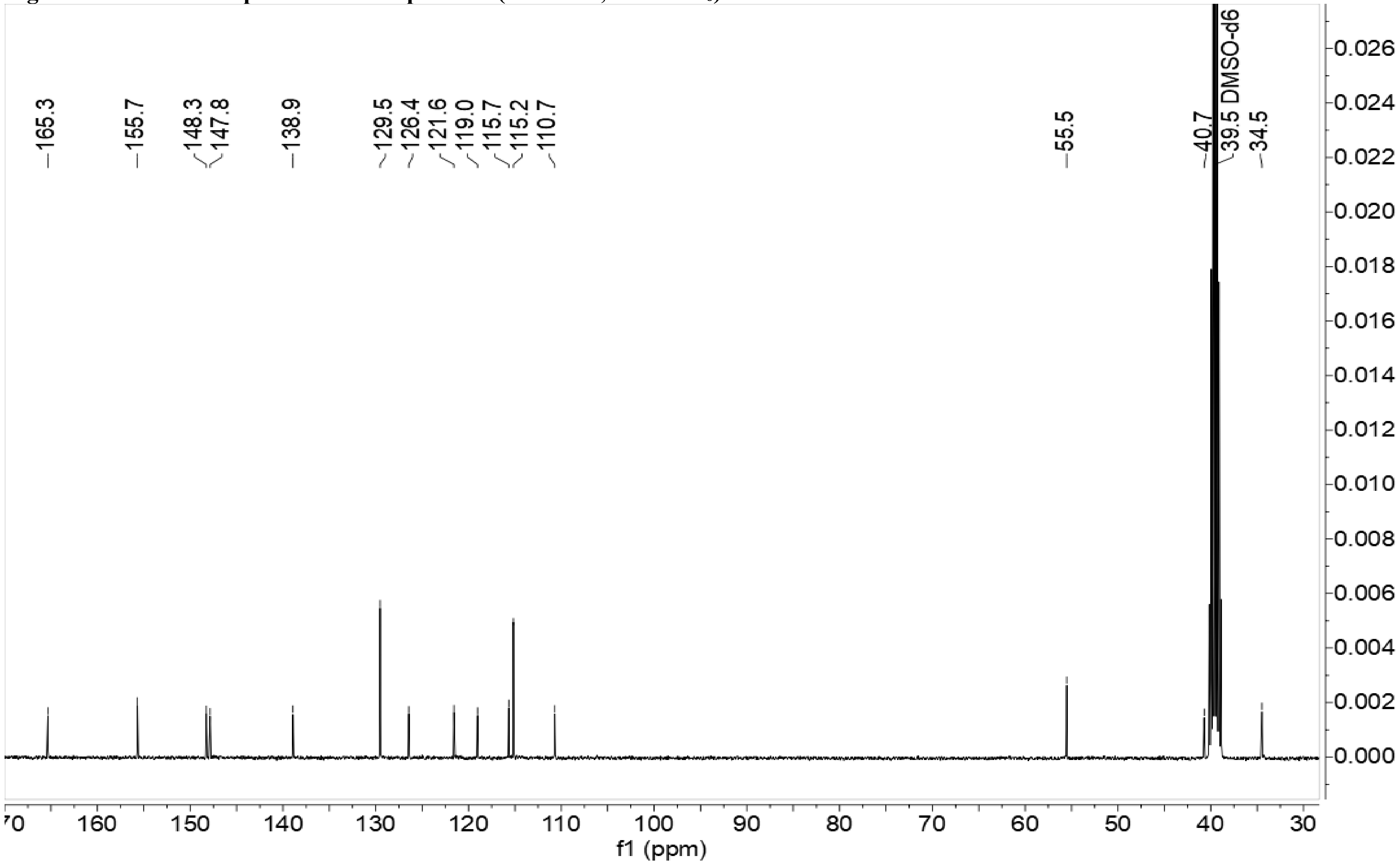

Figure S38. HRESIMS data of compound 8

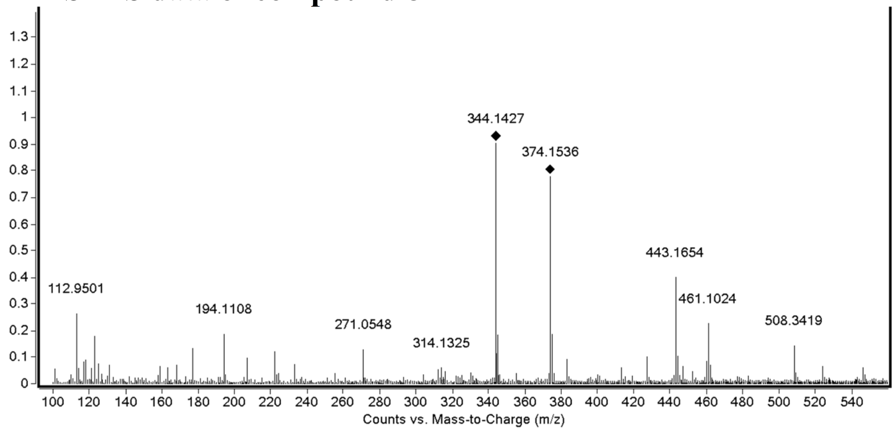

Figure S39. UV spectrum of compound 8

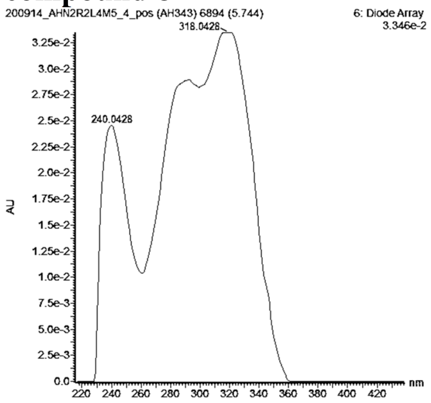

Figure S40. IR(KBr) spectrum of compound 8

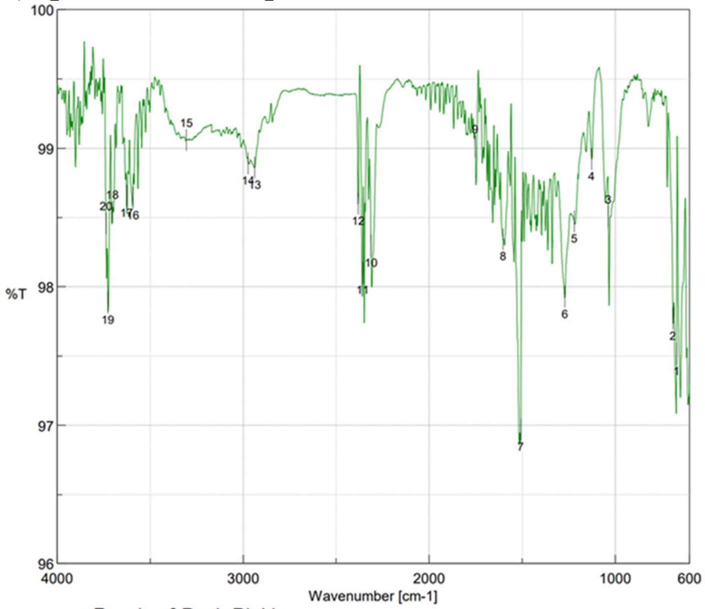

Result of Peak Picking

No.	Position	Intensity	No.	Position	Intensity
1	669.178	97.5138	2	689.427	97.7689
3	1036.55	98.5048	4	1126.22	98.9189
5	1220.72	98.4701	6	1269.9	97.9272
7	1508.06	96.9687	8	1602.56	98.3434
9	1752.01	99.0113	10	2312.23	98.2953
11	2357.55	98.1001	12	2381.66	98.5978
13	2938.02	98.8595	14	2972.73	98.8887
15	3305.39	99.0582	16	3592.73	98.6402
17	3627.45	98.6562	18	3702.66	98.5385
19	3726.76	97.8836	20	3737.37	98.4541

Figure S41. ^{1}**H NMR spectrum of compound 8 (600 MHz, DMSO-*d*$_6$)**

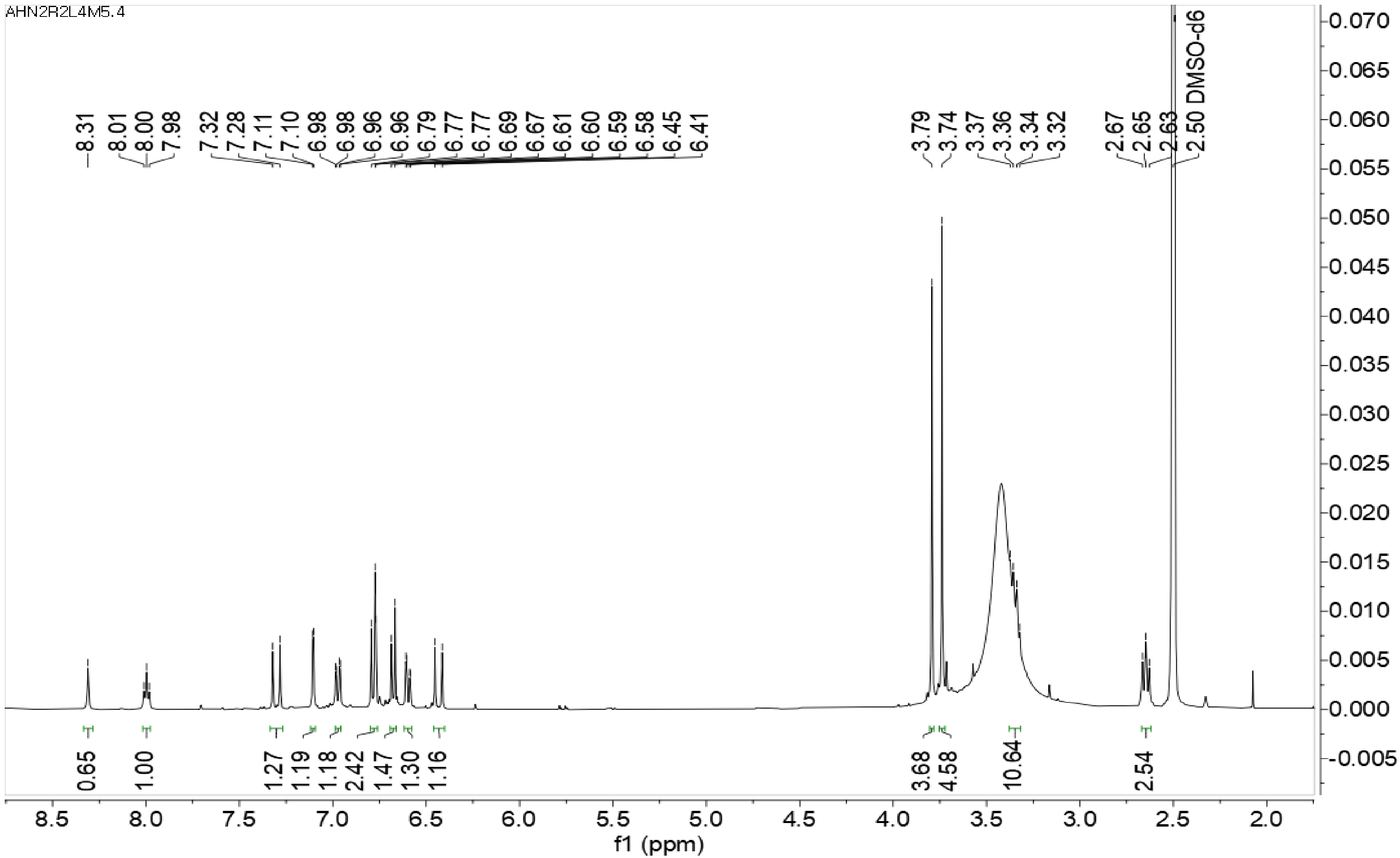

Figure S42. ^{13}C NMR spectrum of compound 8 (150 MHz, DMSO-d_6)

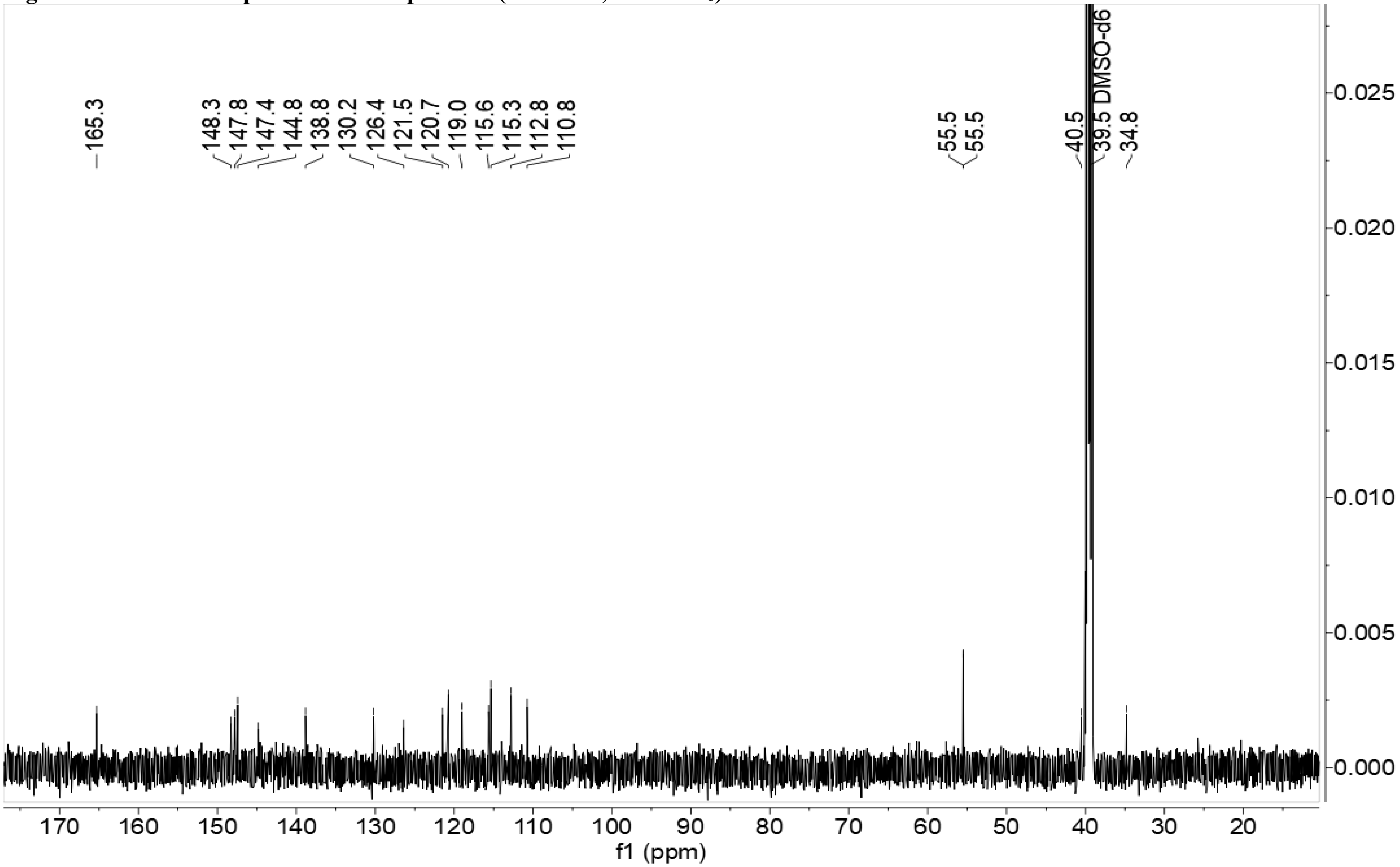

Figure S43. HRESIMS/MS data of compound 9

Mass	RA	Calc. Mass	mDa	PPM	DBE	Formula	i-FIT	i-FIT Norm	Fit Conf %	Frag. Mass	Fragment details	C	H	N	O
313.1084	100.00	313.1076	0.8	2.6	10.5	C18 H17 O5	604.7	6.742	0.12	269.0811	C16 H13 O4 : -0.3 (-1.1)	18	17		5

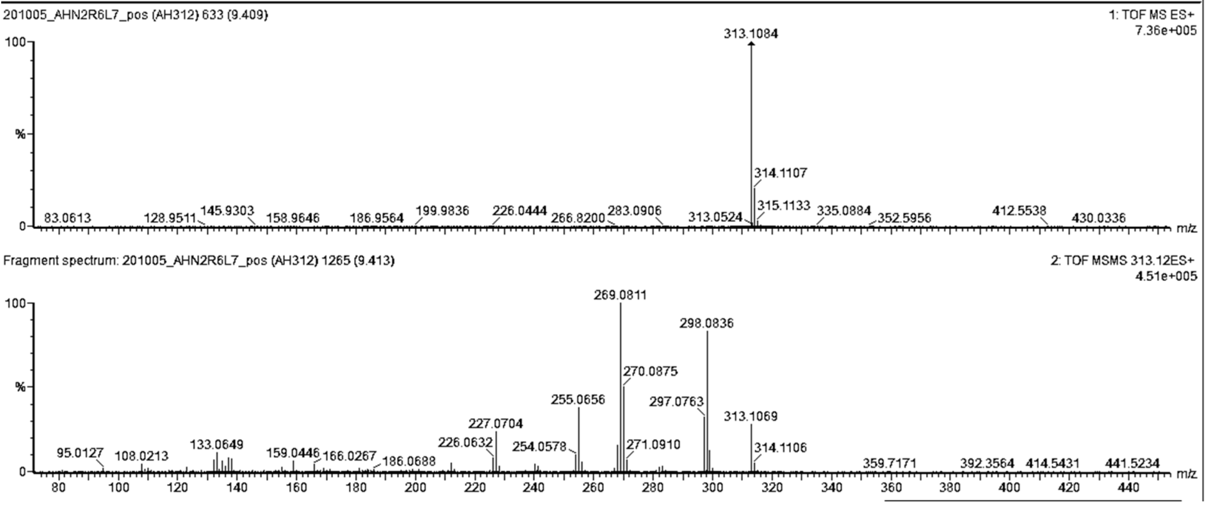

Figure S44. UV spectrum of compound 9

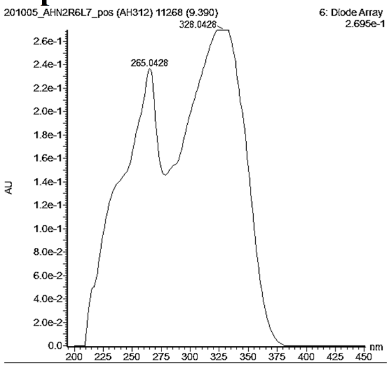

Figure S45. IR(KBr) spectrum of compound 9

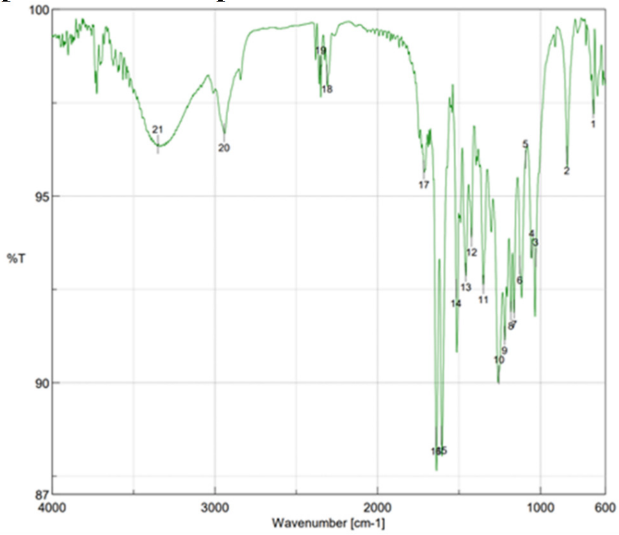

Result of Peak Picking

No.	Position	Intensity	No.	Position	Intensity
1	673.999	97.3239	2	837.919	96.0785
3	1026.91	93.3483	4	1051.98	93.5866
5	1091.51	95.9816	6	1126.22	93.143
7	1160.94	91.9642	8	1181.19	91.9166
9	1220.72	91.2536	10	1255.43	90.2019
11	1349.93	92.6223	12	1424.17	93.891
13	1458.89	92.9518	14	1518.67	92.5161
15	1607.38	88.5779	16	1642.09	88.5546
17	1716.34	95.7	18	2312.23	98.2621
19	2351.77	98.4934	20	2942.84	96.6918
21	3349.75	96.3839			

Figure S46. ^{1}H NMR spectrum of compound 9 (600 MHz, DMSO-d_6)

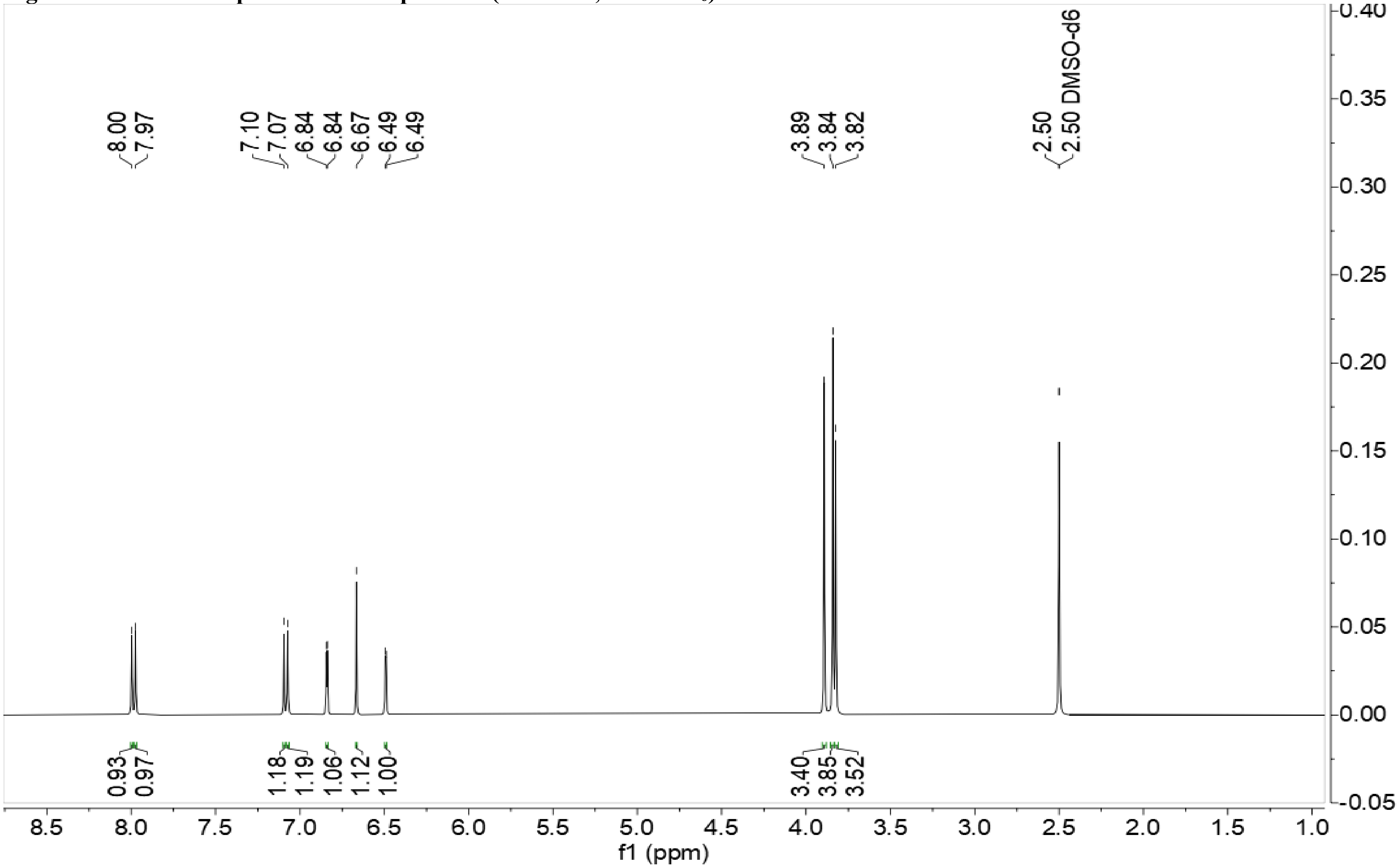

Figure S47. ^{13}**C NMR spectrum of compound 9 (150 MHz, DMSO-*d*$_6$)**

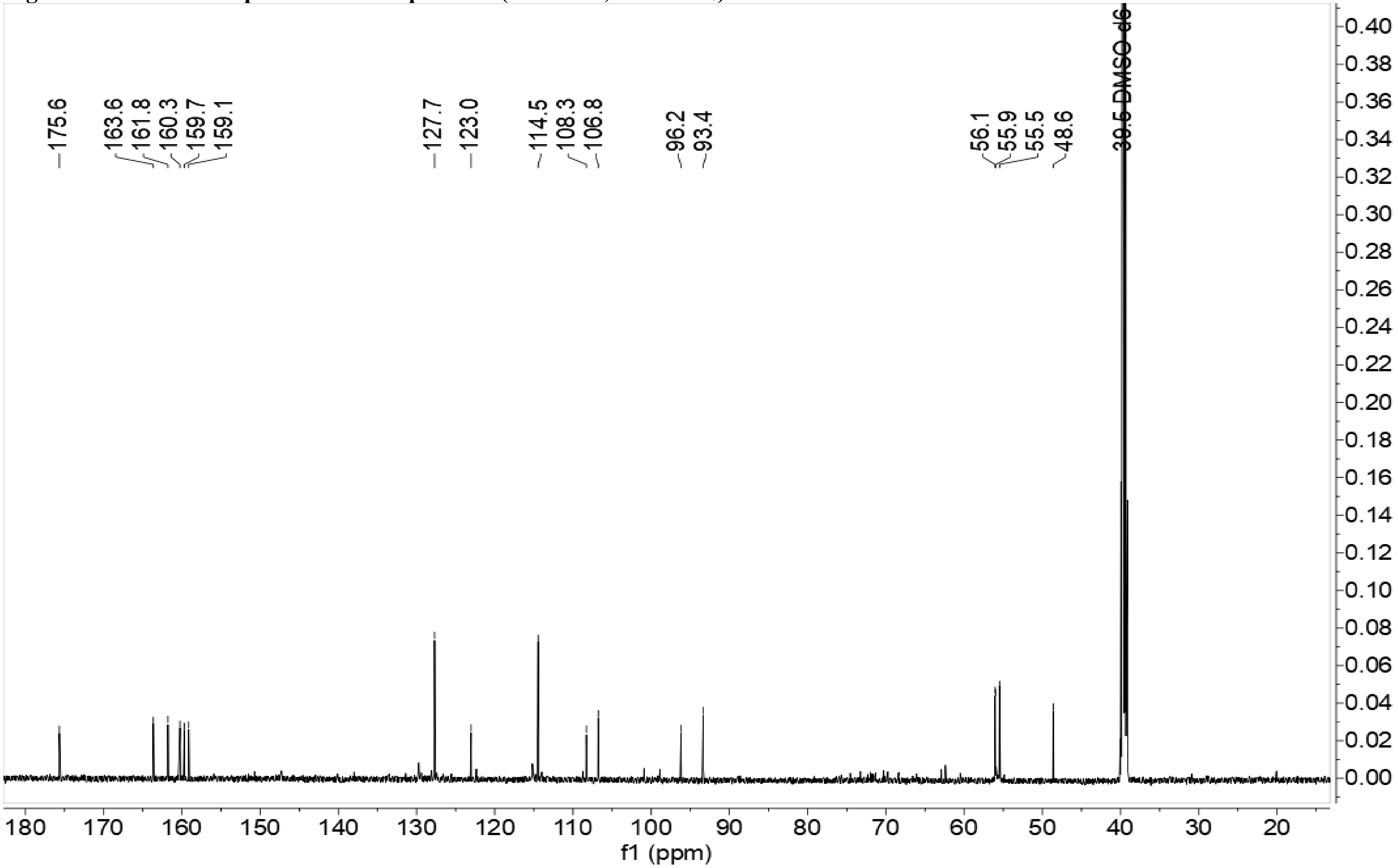

Figure S48. HRESIMS/MS data of compound 10

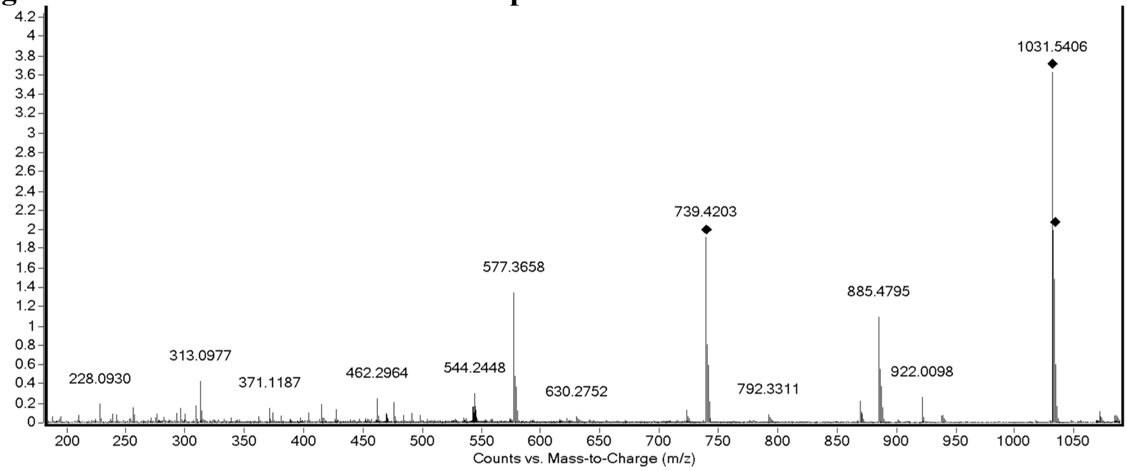

Figure S49. IR(KBr) spectrum of compound 10

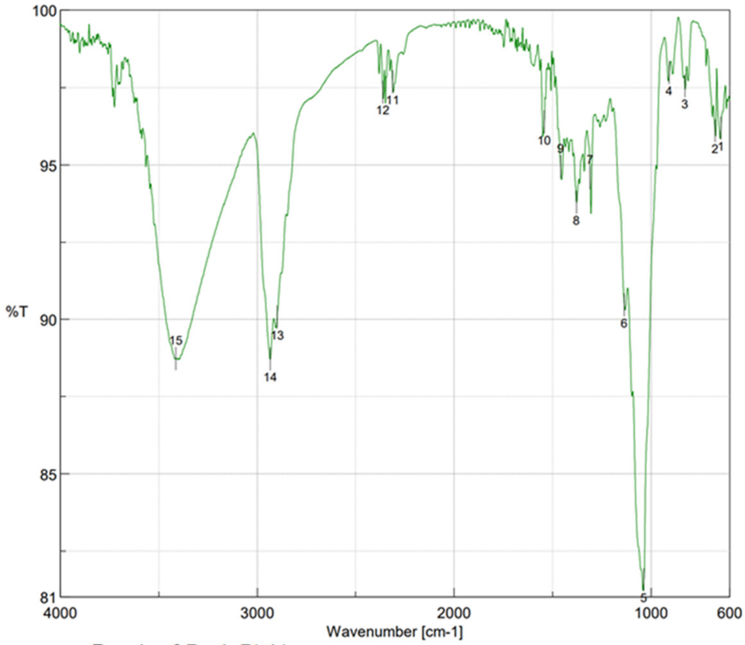

Result of Peak Picking

No.	Position	Intensity	No.	Position	Intensity
1	645.072	96.1801	2	673.999	96.0882
3	828.277	97.5468	4	907.344	97.973
5	1036.55	81.5542	6	1135.87	90.4553
7	1309.43	94.5721	8	1378.85	93.7865
9	1458.89	94.9347	10	1542.77	96.3798
11	2312.23	97.6789	12	2362.37	97.3489
13	2898.49	90.0695	14	2933.2	88.7035
15	3414.35	88.7152			

Figure S50. ^{1}**H NMR spectrum of compound 10 (500 MHz, pyridine-*d*$_5$)**

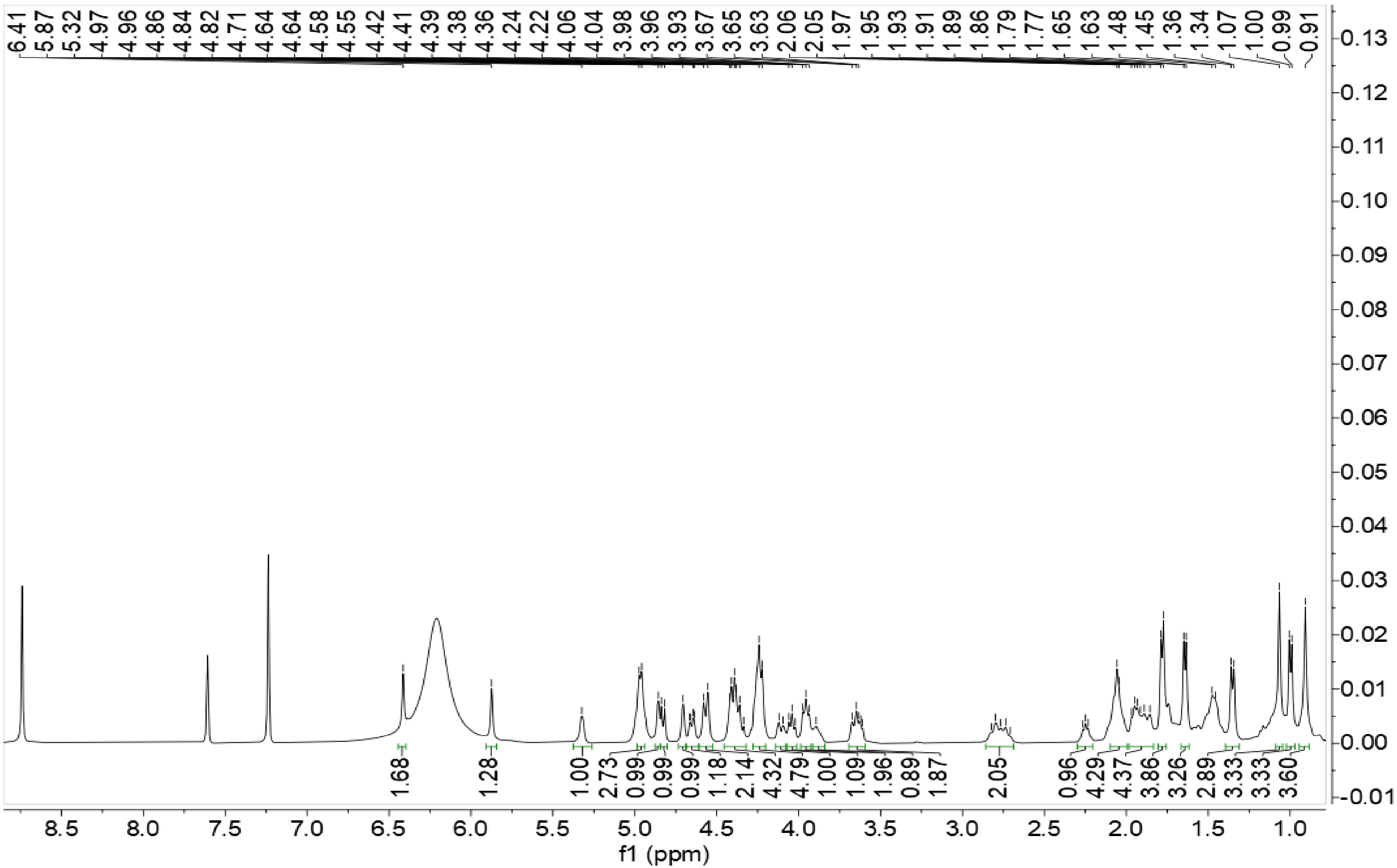

Figure S51. ^{13}C **NMR spectrum of compound 10 (125 MHz, pyridine-*d*$_5$)**

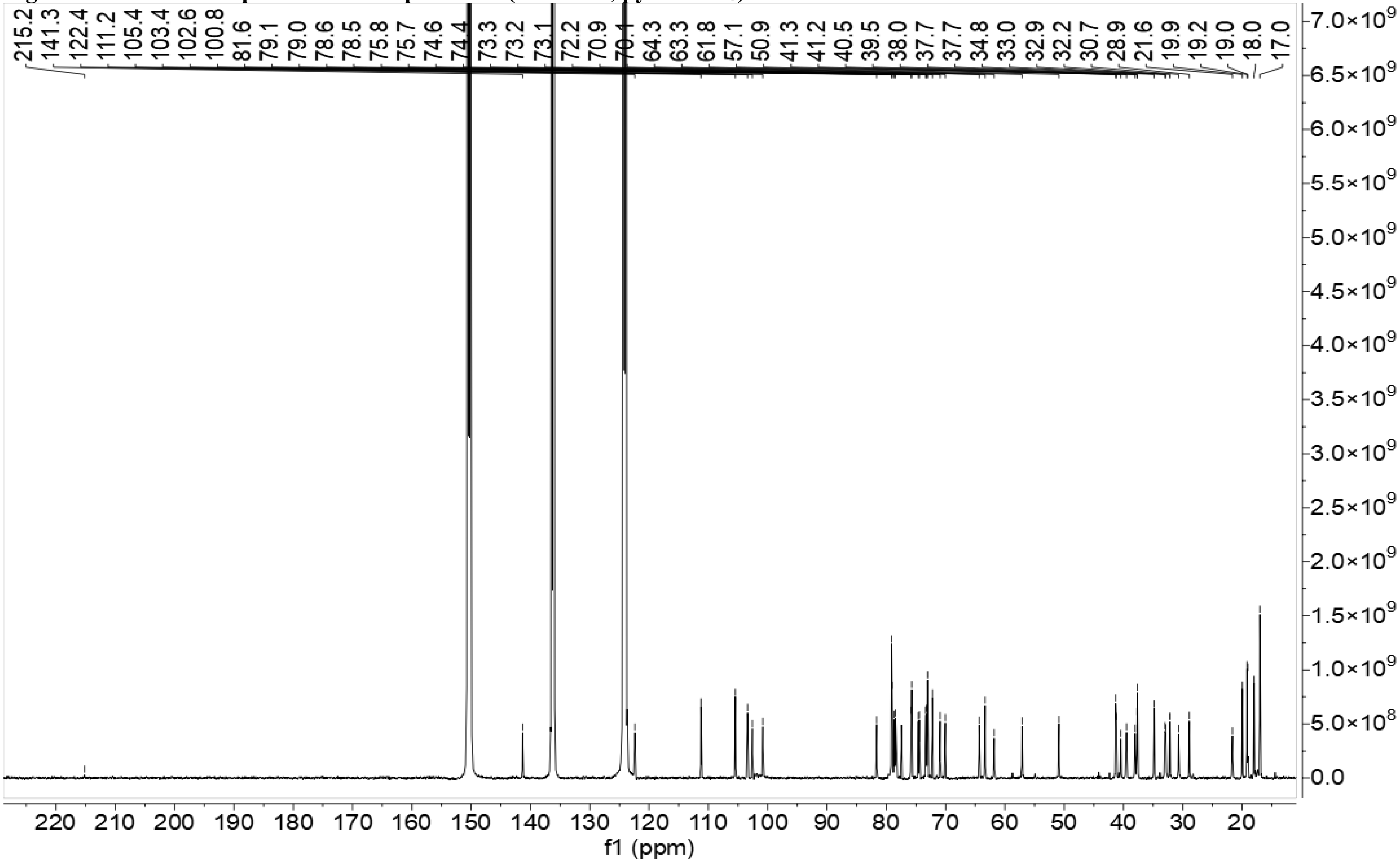

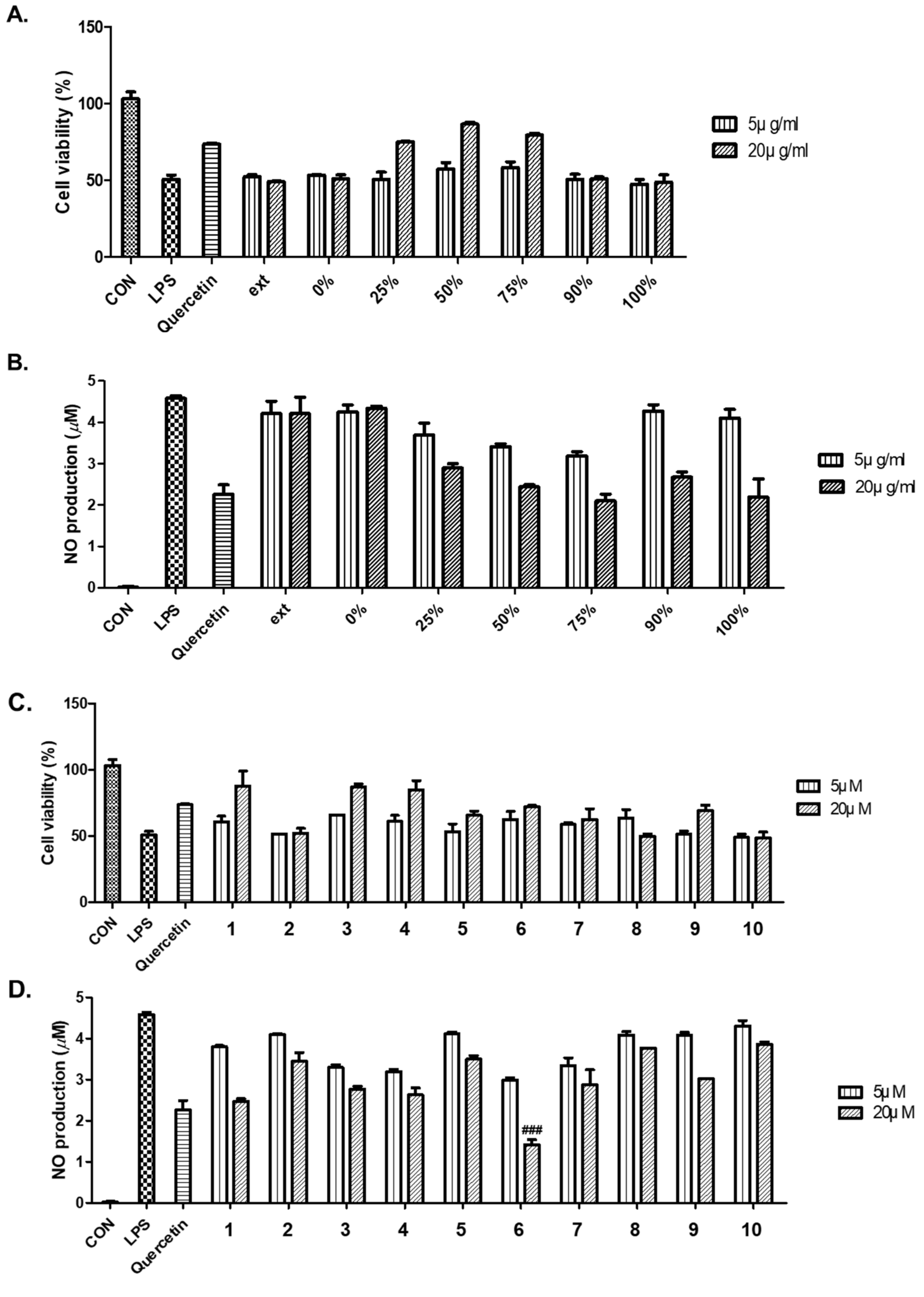

Figure S52. Inhibitory effects of fractions and single compounds from *A. hookeri* on BLM-induced senescence system in RAW 264.7 cells using nitric oxide (NO) assay kit.
(A) Cell viability of total extract and subfractions (0–100% of MeOH) of *A. hookeri* at 5 and 20 μg/mL. (**B**) NO production level of total extract and subfractions (0–100% of MeOH) of *A. hookeri* at 5 and 20 μg/mL. (**C**) Cell viability of single compounds (**1–10**) at 5 and 20 μM. (**D**) NO production level of single compounds (**1–10**) at 5 and 20 μM.

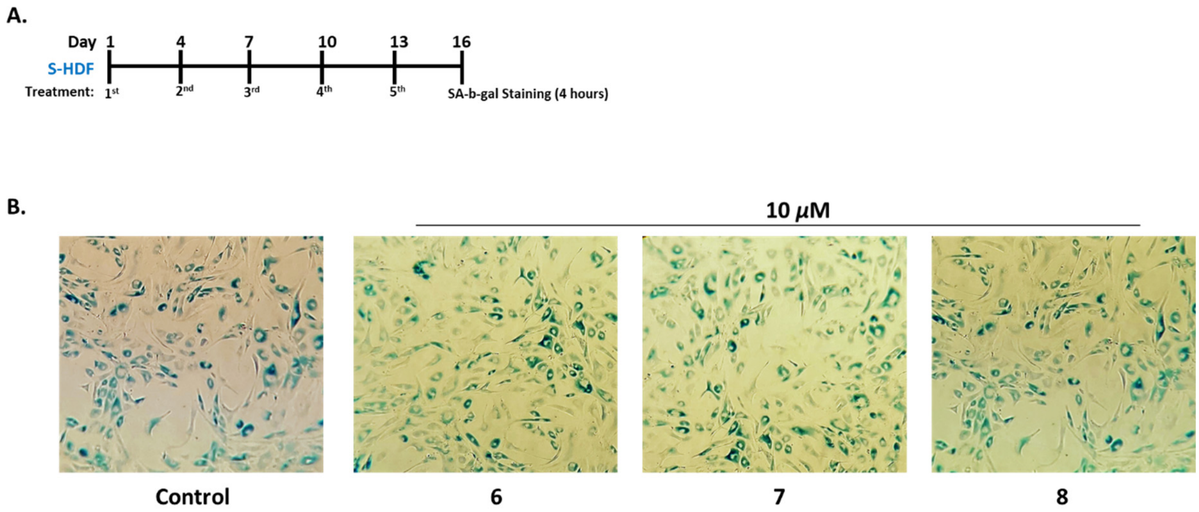

Figure S53. SA-*β*-gal-positive senescent cells on replicative senescent HDF cell. Tested compounds (6–8) were treated at 10 *μ*M of on replicative senescent HDF cell for 15 days by every 3 internal days. No effect on reducing SA-*β*-gal staining was detected.

Table S2. Putative identification of components from leaves of *Allium hookeri* using HRESI-qTOF-MS/MS-based molecular networking. HRESI-qTOF-MS/MS data recorded in positive mode selective for assignment with *m/z* error less than 10 ppm. Molecular networking was created at GNPS (https://gnps.ucsd.edu/ProteoSAFe/status.jsp?task=b6f262ecf9994b82bab562336b008eeb)

No.	Retention time (min)	*m/z*	Compound_Name	GNPSLibraryURL	*MZ* Error (ppm)
**1**	3.13	565.1531	5,7-dihydroxy-2-(4-hydroxyphenyl)-8-[3,4,5-trihydroxy-6-(hydroxymethyl)oxan-2-yl]-6-(3,4,5-trihydroxyoxan-2-yl)chromen-4-one	http://gnps.ucsd.edu/ProteoSAFe/gnpslibraryspectrum.jsp?SpectrumID=CCMSLIB00004709143	3.46
**2**	4.08	463.0861	(2S,3S,4S,5R,6S)-6-[2-(3,4-dihydroxyphenyl)-5-hydroxy-4-oxochromen-7-yl]oxy-3,4,5-trihydroxyoxane-2-carboxylic acid	http://gnps.ucsd.edu/ProteoSAFe/gnpslibraryspectrum.jsp?SpectrumID=CCMSLIB00004719166	1.98
**3**	4.58	271.0577	5,7-dihydroxy-2-(4-hydroxyphenyl)-4H-chromen-4-one	http://gnps.ucsd.edu/ProteoSAFe/gnpslibraryspectrum.jsp?SpectrumID=CCMSLIB00006576166	8.44
**4**	4.82	271.0592	Genistein	http://gnps.ucsd.edu/ProteoSAFe/gnpslibraryspectrum.jsp?SpectrumID=CCMSLIB00005767064	2.93
**5**	5.02	301.0684	Chrysoeriol	http://gnps.ucsd.edu/ProteoSAFe/gnpslibraryspectrum.jsp?SpectrumID=CCMSLIB00000205348	8.72
**6**	5.32	447.0930	apigenin-7-O-glucuronide	http://gnps.ucsd.edu/ProteoSAFe/gnpslibraryspectrum.jsp?SpectrumID=CCMSLIB00004720077	2.18
**7**	5.42	477.1043	(2S,3S,4S,5R,6S)-3,4,5-trihydroxy-6-[5-hydroxy-2-(4-hydroxyphenyl)-6-methoxy-4-oxochromen-7-yl]oxyoxane-2-carboxylic acid	http://gnps.ucsd.edu/ProteoSAFe/gnpslibraryspectrum.jsp?SpectrumID=CCMSLIB00004720062	2.75
**8**	6.41	1031.5452	beta-D-Glucopyranoside, (3beta,22beta,25R)-26-(beta-D-glucopyranosyloxy)-22-hydroxyfurost-5-en-3-yl O-6-deoxy-alpha-L-mannopyranosyl-(1->2)-O-[6-deoxy-alpha-L-mannopyranosyl-(1->4)]	http://gnps.ucsd.edu/ProteoSAFe/gnpslibraryspectrum.jsp?SpectrumID=CCMSLIB00000856131	4.97
**9**	6.96	685.2728	(1R,2S)-7-hydroxy-1-(4-hydroxy-3,5-dimethoxyphenyl)-2-N,3-N-bis[2-(4-hydroxyphenyl)ethyl]-6,8-dimethoxy-1,2-dihydronaphthalene-2,3-dicarboxamide	http://gnps.ucsd.edu/ProteoSAFe/gnpslibraryspectrum.jsp?SpectrumID=CCMSLIB00005722109	4.63
**10**	7.21	325.2239	Denatonium	http://gnps.ucsd.edu/ProteoSAFe/gnpslibraryspectrum.jsp?SpectrumID=CCMSLIB00005755211	9.48
**11**	7.31	271.0593	Aloe-emodin	http://gnps.ucsd.edu/ProteoSAFe/gnpslibraryspectrum.jsp?SpectrumID=CCMSLIB00005761593	2.59
**12**	12.13	415.2113	1-({[2-(2-furylmethyl)-5-methylpyrrolidinyl]amino}methylene)-7-[8-({[2-(2-furylmethyl)pyrrolidinyl]amino}methylene) -1,6-dihydroxy-3-methyl-5-(methylethyl)-7 -oxo(2-naphthyl)]-3,8-dihydroxy-6-methyl-4-(methylethyl)naphthalen-2-one	http://gnps.ucsd.edu/ProteoSAFe/gnpslibraryspectrum.jsp?SpectrumID=CCMSLIB00010124460	1.69
**13**	13.07	299.0907	7,4-Di-O-methylapigenin	http://gnps.ucsd.edu/ProteoSAFe/gnpslibraryspectrum.jsp?SpectrumID=CCMSLIB00006421404	2.35

**14**	13.37	415.3200	Diosgenin	http://gnps.ucsd.edu/ProteoSAFe/gnpslibraryspectrum.jsp?SpectrumID=CCMSLIB00005770460	2.42
**15**	14.11	520.3379	PR310844 LPC 18:2	http://gnps.ucsd.edu/ProteoSAFe/gnpslibraryspectrum.jsp?SpectrumID=CCMSLIB00005747189	5.98
**16**	14.91	518.3202	1-Hexadecanoyl-sn-glycero-3-phosphocholine	http://gnps.ucsd.edu/ProteoSAFe/gnpslibraryspectrum.jsp?SpectrumID=CCMSLIB00003134510	3.53
**17**	15.38	522.3552	1-(9Z-Octadecenoyl)-sn-glycero-3-phosphocholine	http://gnps.ucsd.edu/ProteoSAFe/gnpslibraryspectrum.jsp?SpectrumID=CCMSLIB00003139797	1.52
**18**	16.89	293.2449	14-(hydroxymethyl)-5,9-dimethyltetracyclo[11.2.1.0 , ?.0?,?]hexadecan-5-ol	http://gnps.ucsd.edu/ProteoSAFe/gnpslibraryspectrum.jsp?SpectrumID=CCMSLIB00004713902	7.18
**19**	16.94	524.3692	1-Stearoyl-2-hydroxy-sn-glycero-3-phosphocholine	http://gnps.ucsd.edu/ProteoSAFe/gnpslibraryspectrum.jsp?SpectrumID=CCMSLIB00003139075	4.19
**20**	17.64	439.3561	18-hydroxy-1,2,5,8,15,19,19-heptamethylpentacyclo[12.8.0.0<2,11>.0<5,10>.0<15, 20>]docos-11-ene-8-carboxylic acid	http://gnps.ucsd.edu/ProteoSAFe/gnpslibraryspectrum.jsp?SpectrumID=CCMSLIB00010125896	2.01
**21**	17.64	279.2317	linolenic acid	http://gnps.ucsd.edu/ProteoSAFe/gnpslibraryspectrum.jsp?SpectrumID=CCMSLIB00005738688	1.09
**22**	17.76	355.2836	monolinolein	http://gnps.ucsd.edu/ProteoSAFe/gnpslibraryspectrum.jsp?SpectrumID=CCMSLIB00004692320	1.12
**23**	18.78	282.2793	9 (Z)-Octadecenamide	http://gnps.ucsd.edu/ProteoSAFe/gnpslibraryspectrum.jsp?SpectrumID=CCMSLIB00003139405	1.08
**24**	19.28	357.2980	monoolein	http://gnps.ucsd.edu/ProteoSAFe/gnpslibraryspectrum.jsp?SpectrumID=CCMSLIB00004720009	5.55
**25**	19.48	593.2750	Phaeophorbide a	http://gnps.ucsd.edu/ProteoSAFe/gnpslibraryspectrum.jsp?SpectrumID=CCMSLIB00010128702	1.75
